# Effect of PHRs and PCPs on Microalgal Growth, Metabolism and Microalgae-Based Bioremediation Processes: A Review

**DOI:** 10.3390/ijms20102492

**Published:** 2019-05-20

**Authors:** Krystian Miazek, Beata Brozek-Pluska

**Affiliations:** Institute of Applied Radiation Chemistry, Faculty of Chemistry, Lodz University of Technology, Wroblewskiego 15, 93-590 Lodz, Poland; beata.brozek-pluska@p.lodz.pl

**Keywords:** pharmaceuticals (PHRs), personal care products (PCPs), environmental pollution, wastewaters, bioremediation, microalgal cultivation, biomass production, biomolecules, industrial applications

## Abstract

In this review, the effect of pharmaceuticals (PHRs) and personal care products (PCPs) on microalgal growth and metabolism is reported. Concentrations of various PHRs and PCPs that cause inhibition and toxicity to growths of different microalgal strains are summarized and compared. The effect of PHRs and PCPs on microalgal metabolism (oxidative stress, enzyme activity, pigments, proteins, lipids, carbohydrates, toxins), as well as on the cellular morphology, is discussed. Literature data concerning the removal of PHRs and PCPs from wastewaters by living microalgal cultures, with the emphasis on microalgal growth, are gathered and discussed. The potential of simultaneously bioremediating PHRs/PCPs-containing wastewaters and cultivating microalgae for biomass production in a single process is considered. In the light of reviewed data, the feasibility of post-bioremediation microalgal biomass is discussed in terms of its contamination, biosafety and further usage for production of value-added biomolecules (pigments, lipids, proteins) and biomass as a whole.

## 1. Introduction

Industrial, urban and agricultural activities result in the release of numerous contaminants such as metals, metalloids, solvents, pesticides and organic compounds of pharmaceutical and household origin. Contaminants present in wastewaters enter aquatic and terrestrial environments and cause toxic effects on (micro)organisms within ecosystems. Microalgae are photosynthetic microorganisms situated at the bottom of aquatic food webs. Eco-pollutants exert toxicity towards microalgae and consequently possess an adverse impact on all higher-level organisms within food chains, and further, human surroundings and human beings. Microalgal populations have been reported to be adversely affected by metals and metalloids [1,2], organic solvents [3], pesticides [4] and detergents [5], but also pharmaceuticals (PHRs) and personal care products (PCPs).

PHRs (antibiotics, nonsteroidal anti-inflammatory drugs, fever and pain treatment medicines, antidepressants, lipid regulators, anticancer agents, antiepileptic agents, beta-blockers, estrogens, antianxiety agents, etc.) and PCPs (antiseptic/preservative/disinfectant ingredients) released from hospitals and healthcare facilities, agricultural units, domestic and industrial sources and landfill leachates can enter ecosystems via effluents/wastewaters and cause harmful effects on micro- and higher organisms (algae, daphnids, fish) [6]. Indeed, pharmaceuticals were reported to negatively affect growth of microalgae and protozoa even at very low (ng/L) concentrations [7,8,9]. Therefore, the pollution of the environment by PHRs and PCPs present in wastewaters is of emerging concern. 

A range of different techniques was proposed to bioremediate wastewaters containing pharmaceuticals and personal care products. These techniques are based on chemical (advanced oxidation, photodegradation), physical–chemical (activated carbon adsorption, membrane filtration) and biological (biotransformation and biodegradation by microorganisms) methods. However, all these technologies face their specific limitations and drawbacks in terms of treatment efficiencies, exploitation costs and technical possibilities to be applied on the industrial scale [10,11,12]. Advanced oxidation treatment of pharmaceuticals and personal care products can generate toxic byproducts [11]. Degradation of pharmaceuticals via the photolysis process requires the application of lamps emitting light within specific wavelength ranges [13]. Activated carbon treatment is limited by the very high cost of production of activated carbon, which is additionally regarded as a non-environmentally friendly material [11]. The use of membranes provides low efficiency of pharmaceutical removal and improving their removal efficiency requires high investments and operational costs [11,12]. Performance of anaerobic wastewater treatment processes was reported to be negatively affected by antibiotics that possess inhibitory effects on microorganisms [14]. Moreover, biological treatment of pharmaceutical-containing wastewaters via an activated sludge process can lead to development of bacterial strains resistant, for example, to antibiotics that could pose a serious threat to humans [15]. Therefore, there is emerging necessity to develop new methods for cost-effective and “green” utilization of pharmaceuticals and personal care products.

Successful removal of pharmaceuticals by nonliving microalgae and cyanobacteria biomass has been reported [16,17], but PHRs and PCPs could be also bioremediated by living microalgal cells. Although sensitive to eco-toxicants, such as PHRs and PCPs, microalgae are able to remove these pollutants from wastewater streams as a bioremediation method. Microalgal strains were reported to be capable of biotransforming a range of different pharmaceuticals (antibiotics, anti-inflammatory drugs, antiepileptics, hormones, beta-blockers, pain killers) and personal care products (disinfectants, antiseptics) [10,18]. Apart from the bioremediation approach, microalgae are cultivated for the production of biomass containing natural biomolecules such as lipids, proteins and pigments that can find applications in many branches of industry [19,20].

Applications of microorganisms such as bacteria, microalgae and fungi in the bioremediation of wastewaters contaminated by PHRs and PCPs have been extensively summarized [10,18,21]. In this review, we focus on the effect of PHRs and PCPs on microalgal metabolism and cellular composition, on maximizing microalgal growth and biomass production during wastewater bioremediation, and on the possible application of post-remediation microalgal biomass.

## 2. Sources of the Release of Waste Streams Containing Pharmaceuticals and Personal Care Products

Nowadays, hundreds of pharmaceuticals from different groups are being used in everyday life. Such groups include antibiotics (phenicols, tetracyclines, aminoglycosides, sulphonamides, trimethoprim, β-lactams, quinolones, macrolides, glycopeptides, etc.), nonsteroidal anti-inflammatory drugs (ibuprofen, naproxen, diclofenac, etc.), fever and pain treatment medicines (acetaminophen), antidepressants (fluoxetine, clomipramine, etc.), lipid regulators (gemfibrozil, clofibrate, etc.), antineoplastic agents (tamoxifen, 5-fluorouracyl, cisplatin, etc.), antiepileptic agents (carbamazepine), β-blockers (propranolol, metoprolol), estrogens (ethynylestradiol, estradiol), anesthetic drugs, antianxiety agents, antiviral drugs, antiparasitic drugs and proton pump inhibitors. Besides pharmaceuticals, personal care products contain antiseptic/preservative/disinfectant ingredients such as triclosan, triclocarban, parabens and a few other chemicals. When disposed, numerous PHRs and PCPs enter wastewaters (Table S1).

Hospitals and healthcare facilities generate effluents containing numerous pharmaceuticals. Wastewaters from hospitals were reported to be composed of β-lactam, glycopeptide and trimethoprim antibiotic mixtures [22], mixtures of tetracycline, quinolone and sulphonamide antibiotics [23] or a mixture of quinolone antibiotic and anticancer drugs (tamoxifen, cyclophosphamide) [24]. Metronidazole and ciprofloxacin were detected in wastewaters of a rural and an urban hospital [25]. Agriculture is another source of wastewaters. The use of human and veterinary medicines as well as hormones resulted in the occurrence of antibiotics, nonsteroidal anti-inflammatory drugs, β-blockers and estrogens in agricultural effluents [26]. Indeed, swine wastewaters were reported to contain numerous antibiotics from sulfonamide, tetracycline and macrolide groups, as well as hormones (estradiol and ethynylestradiol) [27]. Municipal wastewaters from domestic and industrial sources can contain a range of different pharmaceuticals and personal care products. Such municipal effluents can be contaminated by the presence of acetaminophen [28]; diclofenac and carbamazepine [29]; ibuprofen, naproxen and triclosan [30]; and hormones [31]. Moreover, wastewater streams were reported to contain not only antibiotics from different classes (β-lactams, sulfonamides, quinolones, tetracyclines, macrolides, etc. [32] and phenicols, aminoglycosides [33]), but also numerous parabens [34]. Indeed, the occurrence of parabens, triclosan and triclocarban was confirmed in sewage streams [35]. Households and hospitals are sources of the release of antidepressant pharmaceuticals (fluoxetine, amitriptyline, sertraline, citalopram, paroxetine) into water streams [36]. Increased consumption of blood lipid regulators (gemfibrozil, bezafibrate, fenofibrate) resulted in the appearance of these compounds in wastewaters [37]. Pharmaceuticals stored in landfills can also contaminate water streams due to leakage [12]. Indeed, landfill leachates were reported to contain ibuprofen, naproxen or carbamazepine [38].

Wastewaters from municipality and industry constitute a serious threat to environment. PHRs and PCPs from wastewaters can enter aquatic and terrestrial environments and cause negative effects on microorganisms such as microalgae.

## 3. Inhibitory Effect of Pharmaceuticals and Personal Care Products on Microalgal Growth

This section presents the inhibitory effect of different pharmaceuticals (antibiotics, NSAIDs, acetaminophen, antidepressants, lipid regulators, antineoplastic agents, antiepileptic agents, beta-blockers, estrogens and other drugs) and components of personal care products on the growth of different cyanobacteria and eukaryotic microalgal strains. Inhibitory/toxicity concentrations of PHRs and PCPs are presented, and conclusions from literature data gathered are provided.

### 3.1. Antibiotics

#### 3.1.1. Phenicols

Phenicols are antibiotics generally possessing the 2,2-dichloro-*N*-[-1-hydroxy-1-(phenyl)propan-2-yl]acetamide structure, including those with additional groups (hydroxyl, nitro, methylsulfonyl, fluoro), and are represented by chloramphenicol, thiamphenicol and florfenicol.

Chloramphenicol, thiamphenicol and florfenicol were reported to inhibit growth of various diatom (*Skeletonema*, *Chaetoceros*), green microalgae (*Tetraselmis*, *Chlorella*, *Selenastrum*, *Scenedesmus*), haptophyte (*Isochrysis*) and cyanobacterial strains.

Chloramphenicol (CAP) at different concentrations caused 50% inhibition/toxicity to different *Scenedesmus*/*Desmodesmus* strains (within 0.47–2.28 mg/L) [39,40], *Pseudokirchneriella subcapitata* (at 2.7 mg/L) [40], *Tetraselmis* strains (within 4–11 mg/L) [41,42] and *Chlorella pyrenoidosa* (at 14 mg/L) [41]. For haptophytes, CAP at 12 mg/L slightly (22%) inhibited [43] and at 41 mg/L reduced by 50% [41] the growth of *Isochrysis galbana*. For diatoms, *Chaetoceros gracilis* growth was almost completely suppressed at 12 mg/L CAP [43]. For cyanobacteria, growth of *Nostoc flagelliforme* was almost completely suppressed in the presence of chloromycetin (chloramphenicol) at ≥25 mg/L [44].

Thiamphenicol (TAP) at different concentrations caused 50% growth inhibition to *Selenastrum capricornutum* (at 8.9 mg/L) [45], *Tetraselmis chuii* (at 38 mg/L) [41], the haptophyte *Isochrysis galbana* (at 158 mg/L) [41] and different *Chlorella* strains (within 522–1283 mg/L) [41,45].

Response to thiamphenicol can be very different amongst cyanobacteria strains. TAP caused 50% growth inhibition to *Microcystis flosaquae* (at ~0.1 mg/L) [46], *Microcystis aeruginosa* (at 0.32 mg/L), *Synechococcus leopoldensis* (at 0.36 mg/L), *Microcystis wesenbergii* (at 0.43 mg/L), *Synechococcus* sp. (at 0.67 mg/L), *Anabaena cylindrica* (at 1.3 mg/L), *Nostoc* sp. (at 3.5 mg/L), *Anabaena flosaquae* (at 13 mg/L) and *Anabaena variabilis* (at 14 mg/L) [47].

Florfenicol (FF) caused 50% growth inhibition/toxicity (Table 1, Table S2) to *Pseudokirchneriella subcapitata* [48], *Tetraselmis* strains [41,42,49], the haptophyte *Isochrysis galbana* [41], *Scenedesmus vacuolatus* [50], different *Chlorella* strains [41,51,52], the diatom *Skeletonema costatum* [53] and culture of the cyanobacterium *Microcystis flosaquae* [46].

#### 3.1.2. Tetracyclines

Tetracyclines are antibiotics generally possessing a 4-(dimethylamino)-1,10,11,12*a*-tetrahydroxy-3,12-dioxo-tetrahydrotetracene-2-carboxamide structure, including those with additional groups (chloro, methyl, hydroxyl, dimethylamino), and are represented by tetracycline, chlortetracycline, oxytetracycline, doxycycline and minocycline. Tetracycline and its derivatives were reported to negatively affect microalgal growth.

Tetracycline (TET) inhibited growth of two green microalgae, *Dictyosphaerium pulchellum* and *Micractinium pusillum*, within a range of 5–30 mg/L. *Dictyosphaerium* was more sensitive towards tetracycline, with complete growth inhibition in the presence of ≥10 mg/L of this antibiotic. *Micractinium* was more resistant to tetracycline, with a ~50% inhibition at 20 mg/L [54]. TET, within 1–3.3 mg/L, caused ~50% growth inhibition/toxicity to *Pseudokirchneriella subcapitata*/*Selenastrum capricornutum* [55,56,57,58]. For another green microalga, tetracycline at 0.28 mg/L (0.63 μM) caused 50% growth inhibition of *Scenedesmus obliquus* [59]. For the cyanobacterium *Microcystis aeruginosa*, TET caused caused ~50% growth inhibition/toxicity within 0.05–5 mg/L [56,57,60,61]. For other cyanobacteria, tetracycline caused inhibitory effects at a broad concentration range. TET was reported to significantly inhibit (at least by 50%) growth of the cyanobacteria *Aphanizomenon gracile*, *Planktotjrix agardhii* and *Chrisosporum berghii*, at 0.0015, 0.003 and 0.1–0.2 mg/L, respectively [61]. For other strains, TET caused 20% growth inhibition to *Synechocystis* (at 10–100 µg/L) [62], 50% toxicity to *Anabaena* (at 6.2 mg/L) [55] and ~50% growth inhibition to *Nostoc flagelliforme* (at 100 mg/L) [44].

Inhibitory effects of tetracycline can differ towards green microalgae and cyanobacteria. *Pseudokirchneriella subcapitata* was reported to be more sensitive to TET than *Anabaena* [55]. On the contrary, *Microcystis aeruginosa* was more sensitive to TET than *Selenastrum capricornutum* [56].

Chlortetracycline (CTC) at different concentrations caused 50% growth inhibition to *P. subcapitata/S. capricornutum* (at 1.2-3.1 mg/L) [56,63] and *Ankistrodesmus fusiformis* (at 3.2 mg/L) [63], and 50% toxicity to *Chlorella pyrenoidosa* (37.8 mg/L (73.4 µmol/L)) [64]. For the cyanobacterium *Microcystis aeruginosa*, CTC within the range of 1–10 mg/L slightly (up to ~10%) inhibited *Microcystis* growth [60], caused 50% toxicity at 15.2 mg/L (29.5 µmol/L) [64], and completely inhibited *Microcystis* growth at 20 mg/L [60]. However, CTC at 0.05 mg/L was also reported to cause 50% growth inhibition to *Microcystis aeruginosa* [56].

Oxytetracycline (OXY), within 0.17–4.5 mg/L, caused 50% growth inhibition/photosynthetic efficiency inhibition/toxicity to *Pseudokirchneriella subcapitata*/*Selenastrum capricornutum* [45,48,63,65,66,67,68]. For other green microalgae, different oxytetracycline concentrations inhibited by 50% the growth of *Ankistrodesmus fusiformis* (at 4.17 mg/L) [63], *Chlorella vulgaris* (at 7 mg/L) [45], *Tetraselmis chuii* (at 11 mg/L) [49], *Tetraselmis suecica* (at 17 mg/L) [42] and *Scenedesmus vacuolatus* (at 40 mg/L) [50].

Response to oxytetracycline can be very different amongst cyanobacteria strains and between different reports. For *Microcystis aeruginosa*, OXY within 0.207–10 mg/L caused 50% growth inhibition/photosynthetic efficiency inhibition/toxicity [47,60,66,68] and started negatively affecting *Microcystis* growth already at 0.01 mg/L [69]. For *Anabaena flosaquae*, 50% growth inhibition/toxicity was reported at 0.39 mg/L [47] and 2.7 mg/L [67]. For other cyanobacteria strains, OXY caused 50% growth inhibition to *Anabaena cylindrica* (at 0.032 mg/L), *Microcystis wesenbergii* (at 0.35 mg/L), *Anabaena variabilis* (at 0.36 mg/L), *Synechococcus leopoldensis* (at 1.1 mg/L), *Synechococcus* sp. (at 2 mg/L) and *Nostoc* sp. (at 7 mg/L) [47]. Amongst different reports, the green microalga *Pseudokirchneriella* was more sensitive to OXY than cyanobacteria *Microcystis* [66] or *Anabaena* [67]*,* but *Microcystis* was also reported to be more sensitive to OXY than *Selenastrum* [68]. 

For cryptomonads, OXY at 1.6 mg/L caused 50% toxicity to *Rhodomonas salina* culture [68].

Doxycycline (DOXY), at 22 mg/L, reduced growth of *Tetraselmis chuii* [70] by 50%, and at 0.33 mg/L, caused 50% toxicity to *Pseudokirchneriella subcapitata* growth [48]. For cyanobacterium, DOXY at 1 mg/L caused (up to 55%) inhibition to *Microcystis aeruginosa* growth [71]. 

Minocycline (MNC), at 0.45 mg/L (0.92 μM), inhibited growth of *Microcystis aeruginosa* by 50% [72].

#### 3.1.3. Aminoglycosides

Aminoglycosides are antibiotics possessing amino sugar structures and are represented by streptomycin, kanamycin, gentamycin and spectinomycin.

Streptomycin (STR), at a concentration of 2.4 mg/L, caused 40% growth inhibition of *Chlorella vulgaris* [73]. For *Selenastrum capricornutum*/*Pseudokirchneriella subcapitata*, STR at 0.133 mg/L caused 50% inhibition to *Selenastrum capricornutum* growth [56], and at 1.5 mg/L, inhibited photosynthetic efficiency by 50% in *Pseudokirchneriella* culture [66]. For *Microcystis aeruginosa*, STR at 0.007 mg/L caused 50% inhibition to *Microcystis* growth [56], and at 0.034 mg/L, inhibited photosynthetic efficiency by 50% in *Microcystis* culture [66]. In both these studies [56,66], the cyanobacterium strain was more sensitive to STR than the green microalga strain. In another study, streptomycin sulfate was tested towards many cyanobacteria and green microalgal strains [74] with the measurement of minimum inhibitory concentrations (MICs). That study also showed that STR-sulfate generally inhibited cyanobacteria to a higher extent than green microalgae [74]. In one more study for cyanobacteria, *Nostoc flagelliforme* was completely suppressed in the presence of STR-sulfate at 1 mg/L [44].

Other representatives of the aminoglycoside group (kanamycin, gentamycin, spectinomycin) can also inhibit microalgal growth. Kanamycin completely inhibited growth of *Dictyosphaerium pulchellum* and, to different (25–100%) extents, growth of *Micractinium pusillum*, within the range 5–30 mg/L [54]. Gentamicin started to negatively affect growth of *Pseudokirchneriella subcapitata* at a concentration of 1.5 mg/L and with 50% growth inhibition at 19.2 mg/L [75]. Kanamycin and gentamicin, at 0.1–1.6 mg/L, were reported to significantly (≥50%) inhibit growth of the cyanobacteria *Microcystis aeruginosa*, *Aphanizomenon gracile* and *Planktotjrix agardhii,* but rather at ≥0.2 mg/L in the case of *Chrisosporum berghii* [61]. Growth of *Nostoc flagelliforme* was completely suppressed in the presence of gentamicin sulfate at 25 mg/L [44], but only partially (up to 27%) inhibited when exposed to kanamycin sulfate at 1–100 mg/L [44]. Spectinomycin at 5 mg/L completely suppressed *Nostoc flagelliforme* growth [44].

#### 3.1.4. Sulphonamides

Sulphonamides are antibiotics possessing a para-amino-benzene-sulfonamide structure with different additional groups (isoxazolyl, pyrimidinyl, pyridinyl, acetyl, thiazolyl), and are represented by sulfamethoxazole, sulfadimethoxine, sulfamethazine, sulfadiazine, sulfapyridine, sulfamonomethoxine, sulfamerazine, sulfacetamide and sulfathiazole. 

Sulfamethoxazole (SMX) caused growth inhibition (Table 2, Table S2) to *Scenedesmus* strains [76,77], *Chlorella vulgaris* [78,79] and *Selenastrum capricornutum/Pseudokirchneriella subcapitata* [45,65,80,81,82]. For other strains, SMX caused 50% toxicity to the cyanobacterium *Synechococcus leopolensis* [82] and diatom *Cyclotella meneghiniana* [82]. In terms of the effect on photosynthesis, SMX inhibited photosynthetic activity in *Desmodesmus subspicatus* culture [83,84] and inhibited photosynthetic efficiency by 50% in *Microcystis aeruginosa* and *Pseudokirchneriella subcapitata* cultures [66].

Sulfadimethoxine (SDM) caused 50% growth inhibition to *Selenastrum capricornutum* (at 2.3 mg/L) [45], *Chlorella vulgaris* (5.19–11.2 mg/L) [45,78] and *Scenedesmus vacuolatus* (at 9.85 mg/L) [76]. Regarding the effect of SDM towards different cyanobacteria strains, 50% growth inhibition was reported for *Microcystis wesenbergii* (at 470 mg/L), *Anabaena cylindrica* (at 480 mg/L), *Microcystis aeruginosa* (at 500 mg/L), *Synechococcus* sp. (at 760 mg/L), *Synechococcus leopoldensis* (at 1100 mg/L), *Anabaena variabilis* (at 1500 mg/L), *Anabaena flosaquae* (>2000 mg/L) and *Nostoc* sp. (>2000 mg/L) [47]. 

Sulfamethazine (SMT) caused 50% growth inhibition to *Scenedesmus obliquus* (at 1.23 mg/L) [77], *Chlorella pyrenoidosa* (at ~8 mg/L) [85], *Pseudokirchneriella subcapitata* (at 8.7 mg/L) [81] and *Scenedesmus vacuolatus* (at 19.52 mg/L) [76]. For *Spirulina platensis*, 50% growth inhibition was reported at 6 mg/L of SMT [86].

Sulfadiazine (SDZ) caused 50% growth inhibition/toxicity to the diatom *Phaeodactylum tricornutum* (at 0.11 mg/L) [87], cyanobacterium *Microcystis aeruginosa* (at 0.135 mg/L) [68], haptophyte *Isochrysis galbana* (at 1.44 mg/L) [87], green microalgae *Selenastrum capricornutum* (within 2.2–7.8 mg/L) [45,68] and *Chlorella vulgaris* (1.33 mg/L) [79] and the cryptomonad *Rhodomonas salina* (at 403 mg/L) [68].

Sulfapyridine (SP) caused 50% growth inhibition to *Chlorella vulgaris* (at 1–1.93 mg/L) [78] and *Scenedesmus vacuolatus* (at 5.28 mg/L) [76].

Sulfamonomethoxine (SMM) caused 50% toxicity to *Pseudokirchneriella subcapitata* (at 1.34 mg/L) [48], *Chlorella vulgaris* (at 5.9 mg/L) [88] and *Isochrysis galbana* (at 9.7 mg/L) [88].

Sulfamerazine (SMR) at 11.9 mg/L caused 50% inhibition of *Scenedesmus vacuolatus* growth [76].

Sulfacetamide (SCA) at 14.6 mg/L caused 50% inhibition of *Chlorella vulgaris* growth [79].

Sulfathiazole (STZ) at 17.74 mg/L caused 50% inhibition of *Chlorella vulgaris* growth [79].

#### 3.1.5. Trimethoprim

Trimethoprim (TMP) is an antibiotic that possesses a 5-[(3,4,5-trimethoxyphenyl)methyl]pyrimidine-2,4-diamine structure and has been tested for its effect on many microalgal strains.

Trimethoprim, within 40–130 mg/L, caused 50% growth inhibition/toxicity to *Pseudokirchneriella subcapitata*/*Selenastrum capricornutum* [45,67,68,81,89]. For other green microalgae, TMP at 90–123 mg/L (48–72 h) caused 50% growth inhibition to *Chlorella vulgaris* [78], and within 7.8–125 μg/L, caused 34 ± 6% inhibition of photosynthetic activity in *Desmodesmus subspicatus* culture [83].

For diatoms, TMP inhibited by 50% the growth of *Phaeodactylum tricornutum* within 5.1–21.6 mg/L (74.6 µmol/L)) [90,91] and *Navicula pelliculosa* (at 2.13 mg/L (7.36 µmol/L)) [91].

For cryptomonads, TMP at 16 mg/L caused 50% toxicity to *Rhodomonas salina* culture [68].

Response to trimethoprim can be very different amongst cyanobacteria strains. Growth inhibition of 50% was reported for *Anabaena variabilis*, *Nostoc* sp. and *Microcystis aeruginosa* at 11, 53 and 150 mg/L, respectively, but *Anabaena cylindrica*, *Anabaena flosaquae*, *Microcystis wesenbergii*, *Synechococcus leopoldensis* and *Synechococcus* sp. growths were inhibited by 50% at concentrations higher than 200 mg/L [47]. In other studies with *Anabaena flosaquae*, TMP within 83 mg/L (286 µmol/L) and 253 mg/L caused 50% growth inhibition/toxicity [67,91]. For *Aphanizomenon gracile*, TMP at 0.003–1.6 mg/L exerted partial growth inhibition (up to 50%) [61].

Based on gathered literature data, it cannot be stated which group of microalgae is more susceptible to trimethoprim. TMP was twice as toxic to *Pseudokirchneriella subcapitata* than to *Anabaena flosaquae* [67]. On the contrary, TMP inhibited photosynthetic efficiency by 50% in *Pseudokirchneriella subcapitata* and *Microcystis aeruginosa* cultures at >9 mg/L and 6.9 mg/L, respectively [66]. In another study, TMP at 112 mg/L caused 50% toxicity to *Microcystis aeruginosa* culture, and *Microcystis* was only slightly more sensitive than *Selenastrum capricornutum* (at 130 mg/L) [68].

#### 3.1.6. β-lactams

β-lactam antibiotics possess a β-lactam ring in their structure and can be divided into penicillins and cephalosporins. In penicillins, β-lactam ring is fused to a (dimethyl)(carboxyl)thiazolidine ring. In cephalosporins, β-lactam ring is fused to a (carboxyl)dihydrothiazine ring.

##### Penicillins

Benzylpenicillin (penicillin G) contains a 6-[(2-phenylacetyl)amino] moiety in its penicillin structure. Penicillin G, at 0.006 mg/L, caused 50% inhibition of *Microcystis aeruginosa* growth [56], but at 7114 mg/L, caused 50% toxicity to *Pseudokirchneriella subcapitata* growth [58]. 

##### Aminopenicillins

Ampicillin (AMP) and amoxicillin (AMX) contain a 6-[(2-amino-2-phenylacetyl)amino] moiety or a 6-[[2-amino-2-(4-hydroxyphenyl)acetyl]amino] moiety in their penicillin structures, respectively. 

Eukaryotic microalgae seem to be less or even not susceptible to aminopenicillin antibiotics. Amoxicillin (at 1.5–2 g/L) and ampicillin (at 1–2 g/L) did not show any inhibition/toxicity to *Pseudokirchneriella subcapitata*/*Selenastrum capricornutum* growth [45,55,75], although in one study, 50% toxicity concentration of amoxicillin for *P. subcapitata* was ~0.5 g/L [48]. In other studies, ampicillin at 1 g/L did not negatively influence growth of *Chlorella vulgaris* [45], but amoxicillin at high concentration (3.1 g/L) caused 50% toxicity to *Rhodomonas salina* culture [68].

Response to aminopenicillins can be very diferent amongst cyanobacteria strains.

Ampicillin, at different concentrations, caused 50% growth inhibition to *Microcystis aeruginosa* (at 0.0002 mg/L), *Synechococcus* sp. (at 0.0069 mg/L), *Microcystis wesenbergii* (at 0.013 mg/L), *Synechococcus leopoldensis* (at 0.083 mg/L), *Anabaena cylindrica* (at 0.14 mg/L), *Anabaena variabilis* (at 2.2 mg/L), *Anabaena flosaquae* (at 3.3 mg/L) and *Nostoc* sp. (>200 mg/L) [47]. In another study, ampicillin at ≥25 mg/L significantly inhibited growth of *Nostoc flagelliforme* [44].

Amoxicillin was reported to inhibit growth of the cyanobacteria *Microcystis aeruginosa*, *Aphanizomenon gracile*, *Chrisosporum berghii* and *Planktotjrix agardhii*, generally from the concentration of 0.0015 mg/L upwards, although the rate of inhibition was dependent on the antibiotic and cyanobacterium strain tested, and in the case of *Microcystis*, inhibition at 0.0015 mg/L was not observed [61]. In other studies, amoxicillin caused 50% toxicity to *Anabaena* (at 56 mg/L) [55], *Synechococcus leopolensis* (at 2.2 μg/L) [92] and *Microcystis aeruginosa* (at 3.7 μg/L) [68] cultures.

##### Cephalosporins

Cephalosporins are represented by cephalothin, cefazolin, ceftiofur, cefotaxime, cefapirin, cefradine, ceftazidime and ceftriaxone, which possess different side groups.

Green microalgae seem to be less susceptible to cephalosporin antibiotics. Cephalothin started to negatively affect growth of *Pseudokirchneriella subcapitata* at a concentration of 76 mg/L and with a 48% growth inhibition at 600 mg/L [75], but cefazolin (at 1 g/L) did not negatively influence growth of *Selenastrum capricornutum* [45]. Cefotaxime at 430 mg/L caused 50% toxicity to *Pseudokirchneriella subcapitata* growth [48]. Ceftiofur and cefapirin, at concentrations up to 98 µM (53 and 43.6 mg/L, respectively), did not show any apparent effect on *Scenedesmus* species growth [93], but cefradine (at 12 mg/L) caused ~50% inhibition of *Scenedesmus obliquus* population growth [94].

For cyanobacteria, ceftazidime and ceftriaxone were reported to inhibit growth of *Microcystis aeruginosa*, *Aphanizomenon gracile*, *Chrisosporum berghii* and *Planktotjrix agardhii* within 13 days, generally from the concentration of 0.0015 mg/L upwards, although the rate of inhibition was dependent on the antibiotic and cyanobacterium strain tested, and in some cases for ceftazidime, inhibition at 0.0015 mg/L was not observed [61]. In another study, cefradine at ≥3 mg/L caused complete growth inhibition and death to *Microcystis aeruginosa* culture [94].

#### 3.1.7. Quinolones

Quinolone antibiotics (enrofloxacin, norfloxacin, levofloxacin, ciprofloxacin, sarafloxacin, oxolinic acid), possessing 4-quinolone in their structures and different additional groups (fluoro, piperazinyl, cyclopropyl, ethyl, fluorophenyl and others), and benzo quinolizine antibiotics (flumequine) were reported to negatively affect growth of various microalgae.

Enrofloxacin (ENR), at 100 mg/L, caused strong (73–87%) inhibition of various microalgal strains such as *Chlamydomonas mexicana*, *Scenedesmus obliquus*, *Chlorella vulgaris*, *Ourococcus multisporus* and *Micractinium resseri* [95]. In other studies, ENR caused 50% growth inhibition to *Scenedesmus obliquus* (within 38–88 mg/L) [96,97], *Ankistrodesmus fusiformis* (at 10.6 mg/L) [98] and *Pseudokirchneriella subcapitata* (5.18 mg/L) [98]. For cyanobacteria, ENR at 0.031–0.054 mg/L caused 50% inhibition to *Microcystis aeruginosa* growth [96].

Norfloxacin (NOR) caused 50% inhibition (at 18 mg/L) [81] and 40–50% toxicity (56.4–100 mg/L) [48,55] to *Pseudokirchneriella subcapitata* growth. NOR at 38.5 mg/L inhibited growth of *Scenedesmus obliquus* by 50% [99].

Response to norfloxacin can be very different amongst cyanobacteria strains. NOR at different concentrations caused 50% growth inhibition to *Microcystis wesenbergii* (at 0.038 mg/L), *Anabaena cylindrica* (at 0.053 mg/L), *Microcystis aeruginosa* (at 0.062 mg/L)*, Anabaena variabilis* (at 0.19 mg/L), *Anabaena flosaquae* (at 0.29 mg/L), *Synechococcus leopoldensis* (at 0.63 mg/L), *Synechococcus* sp. (at 0.63 mg/L) and *Nostoc* sp. (at 1.7 mg/L) [47]. In another study, NOR at different concentrations (0.006–1.6 mg/L) was reported to significantly (up to 90%, depending on the strain) inhibit growth of the cyanobacteria *Microcystis aeruginosa*, *Aphanizomenon gracile*, *Chrisosporum berghii* and *Planktotjrix agardhii*, with the *Microcystis* strain being the most sensitive (with 70% inhibition at 0.006 mg/L and 90% at 0.05 mg/L) [61]. In one more study, NOR at 5.6 mg/L caused 50% toxicity to *Anabaena* [55].

Levofloxacin (LEV) caused 50% inhibition to *Chlorella vulgaris* (58–140 mg/L) growth [100] and *Scenedesmus obliquus* (58–273 mg/L) growth [101] and 40% toxicity to *Pseudokirchneriella subcapitata* (80–100 mg/L) [55]. For cyanobacteria, LEV at 4.8 mg/L caused 50% toxicity to *Anabaena* [55], but even at ≥40 μg/L, totally blocked the growth of *Microcystis flosaquae* [102].

Ciprofloxacin (CPFX) caused 40–50% inhibition (2.5–11.3 mg/L) [75,80,81] and 50% toxicity (6.7–8.8 mg/L) [48,103] to *S. capricornutum*/*P. subcapitata*/*R. subcapitata* growth. For other green microalgae, CPFX (within 20–40 mg/L) inhibited *Chlorella vulgaris* by 50% [104,105,106]; at 100–150 mg/L, caused ≥30% inhibition to *Chlorella pyrenoidosa* biomass production/growth [107]; and within 55–111 mg/L, inhibited *Chlamydomonas mexicana* growth by 50% [108]; but at ~6 mg/L, caused 50% toxicity to *Scenedesmus obliquus* and *Desmodesmus quadricauda* growth [103]. 

For diatoms, CPFX inhibited by 50% the growth of *Cylindrotheca closterium* (at 55 mg/L) and *Navicula ramosissima* (at 72 mg/L) [109]. For cyanobacteria, CPFX caused 50% toxicity to *Microcystis aeruginosa* growth already at 0.02 mg/L [103].

Ofloxacin (OFX), within 1.44–5.3 mg/L, caused 50% toxicity to *Pseudokirchneriella subcapitata* growth [48,65,82]. For diatoms, OFX at 0.09 mg/L caused 50% toxicity to *Cyclotella meneghiniana* growth [82]. For cyanobacteria, OFX at 0.016 mg/L caused 50% toxicity to *Synechococcus leopolensis* growth [82]. 

From a series of other quinolones (sarafloxacin, flumequine, oxolinic acid) tested on *Selenastrum capricornutum*, *Rhodomonas salina* and *Microcystis aeruginosa* cultures [68], *Microcystis* showed higher sensitivity to all three antibiotics than the other microalgae, as 50% toxicity to *Microcystis* culture was reported for sarafloxacin (SF) (at 0.015 mg/L), flumequine (F) (at 0.159 mg/L) and oxolinic acid (OA) (at 0.18 mg/L). SF (at 24 mg/L), F (at 18 mg/L) and OA (at 10 mg/L) caused 50% toxicity to the cryptomonad *Rhodomonas salina*. SF (at 16 mg/L), F (at 5 mg/L) and OA (at 16 mg/L) caused 50% toxicity to *Selenastrum capricornutum* [68].

#### 3.1.8. Macrolides

Macrolide antibiotics (erythromycin, clarithromycin, spiramycin, roxithromycin, tylosin, nystatin), possessing a macrocyclic lactone ring to which one or more (amino)deoxysugars are attached, were tested in terms of their effect on microalgal growth.

Erythromycin (ERY), within 0.02–0.35 mg/L, caused 50–90% inhibition/50% toxicity to *Pseudokirchneriella subcapitata*/*Selenastrum capricornutum* growth [55,65,80,110]. For other green microalgae, ERY at 0.36 mg/L caused 50% inhibition of *Chlamydomonas reinhardtii* growth [111], but at 10–12 mg/L, exerted 50% toxicity to *Chlorella vulgaris* or *Ankestrodesmus falcatus* growth [112].

For diatoms, ERY at 1.31 mg/L exerted 50% inhibition of *Phaeodactylum tricornutum* growth [111], an effect which was similar to the study where the growth of *Chaetoceros gracilis* was inhibited by ≥50% in the presence of ERY at ≥1 mg/L [43].

Response to erythromycin can be very different amongst cyanobacteria strains. For instance, 50% growth inhibition was reported for *Microcystis aeruginosa*, *Microcystis wesenbergii*, *Anabaena cylindrica*, *Synechococcus leopoldensis*, *Nostoc* sp., *Synechococcus* sp., *Anabaena flosaquae* and *Anabaena variabilis* at 0.023, 0.023, 0.035, 0.16, 0.2, 0.23, 0.27 and 0.43 mg/L, respectively [47]. Other studies present quite similar results, as 50% toxicity was exerted on *Anabaena* in the presence of 0.022 mg/L ERY [55] and an 82% inhibition of *Microcystis flosaquae* growth was reported at 40 μg/L of erythromycin [113]. Apart from that, growth of *Pseudanabaena planctonica* was completely reduced in the presence of ERY at 10 mg/L [114].

Clarithromycin (CLR), within 0.002–0.23 mg/L, caused 50% inhibition of *Pseudokirchneriella subcapitata* growth [65,81,110,115,116]. For other strains, CLR inhibited 50% of growth of the green microalga *Desmodesmus subspicatus* and cyanobacterium *Anabaena flosaquae* at 32–37 and 5.6–12 µg/L, respectively [117].

Spiramycin (SPI), at 2.3 mg/L, caused 50% inhibition of *Selenastrum capricornutum* growth. The same study showed that *Microcystis aeruginosa* was 460 times more sensitive to SPI than *Selenastrum* [56]. High sensitivity of *Microcystis aeruginosa* was confirmed in another study, where SPI caused 50% inhibition at 1.1 μg/L [118].

Roxithromycin (ROX), at 0.047–0.73 mg/L, inhibited growth of *Pseudokirchneriella subcapitata* by 50% [40,81]. For *Scenedesmus* strains, growth of *Scenedesmus quadricauda*, *Scenedesmus obliquus* and *Scenedesmus acuminatus* was inhibited by 50% in the presence of ROX at 0.129, 0.077 and 2.87 mg/L, respectively [40].

Azithromycin (AZI), at 0.005 mg/L, caused 50% toxicity to *Pseudokirchneriella subcapitata* growth [48].

Tylosin (TYL), within 0.21–4.41 mg/L, inhibited growth of *Pseudokirchneriella subcapitata*/*Selenastrum capricornutum* by 50% [56,81,91], but in another study, TYL inhibited photosynthetic efficiency by 50% in *Pseudokirchneriella* already at 0.0089 mg/L [66].

For cyanobacteria strains tested, growth of *Anabaena flosaquae* and *Synechococcus leopoliensis* was inhibited by 50% in the presence of 0.098 mg/L and 0.096 mg/L TYL, respectively [91]. For *Microcystis aeruginosa*, TYL at 0.034 mg/L caused 50% growth inhibition [56], but at 0.29 mg/L, inhibited photosynthetic efficiency by 50% in the culture of this strain [66].

Based on gathered literature data, it cannot be stated which group of microalgae is more susceptible to tylosin. In one report, *M. aeruginosa* was 40 times more sensitive to TYL than *S. capricornutum* [56], but in another, *P. subcapitata* was 32 times more sensitive to TYL than *M. aeruginosa* [66].

For diatoms, TYL inhibited by 50% the growth of *Cylindrotheca closterium* (at 0.27 mg/L), *Navicula ramosissima* (at 0.99 mg/L), *Navicula pelliculosa* (1.42 mg/L) and *Phaeodactylum tricornutum* (6.07 mg/L) [91,109].

Nystatin, a polyene antimycotic agent from a subgroup of macrolides, was reported to inhibit growth of *Euglena gracilis*, *Chlorella vulgaris*, *Scenedesmus obliquus*, *Chlamydomonas reinhardtii*, *Ochromonas malhamensis* and *Plectonema boryanum*, with *Euglena* strains being more resistant, and with no effect on *Navicula pelliculosa* [119].

#### 3.1.9. Other Antibiotics

##### Glycopeptides

Vancomycin (VAN), a branched glycosylated peptide, caused 50% inhibition/toxicity to *Pseudokirchneriella subcapitata* growth within 371–724 mg/L [58,75].

##### Lincosamides

Lincosamide antibiotics are represented by lincomycin and clindamycin. Lincomycin is a pyrrolidinecarboxamide and a *S*-glycosyl compound. Clindamycin is a 7-chloro-7-deoxylincomycin. 

Lincomycin (LIN) caused 50% growth inhibition/toxicity (Table 3, Table S2) to *Desmodesmus subspicatus* [91], *Pseudokirchneriella subcapitata* [65,91,120], *Cyclotella meneghiniana* [120], *Cylindrotheca closterium* and *Navicula ramosissima* [109], *Anabaena flosaquae* [91] and *Synechococcus leopoliensis* [91,120].

Regarding other lincosamides, clindamycin (CLI) at 0.01 mg/L caused 50% inhibition of *Pseudokirchneriella subcapitata* growth [115].

##### Nitrofurans

Nitrofuran antibiotics possess a furan ring with a nitro group and an additional group such as 1-(methylideneamino)imidazolidine-2,4-dione (present in nitrofurantoin) or 3-(methylideneamino)-1,3-oxazolidin-2-one (present in furazolidone).

Nitrofurantoin (NIT), within 12–17 mg/L, caused 50% growth inhibition of *Desmodesmus subspicatus* [121], but in another study, at 500 μg/L, caused 70% inhibition of photosynthetic activity in *Desmodesmus subspicatus* culture [83]. Furazolidone, at 1.3 mg/L, caused 50% inhibition of *Chlorella pyrenoidosa* growth [122], but at 0.5 mg/L, completely inhibited growth of *Isochrysis galbana* and *Chaetoceros gracilis* [43].

##### Nitroimidazoles

Metronidazole, a 2-(2-methyl-5-nitroimidazol-1-yl)ethanol, is an antibiotic tested in terms of its inhibitory effect towards microalgae.

At 12.5–38.8 mg/L, metronidazole caused 50% toxicity to *Chlorella* sp. [123], and at 40.4–56.6 mg/L, caused 50% toxicity to *Pseudokirchneriella subcapitata*/*Selenastrum capricornutum* growth [48,123]. For *Desmodesmus*/*Scenedesmus* strains, metronidazole (within 7.8–125 μg/L) caused 50% inhibition of photosynthetic activity in *Desmodesmus subspicatus* culture [83], but in another study, at 705 mg/L, caused 50% inhibition to *Scenedesmus vacuolatus* [50]. For the cyanobacterium *Microcystis protocystis*, high resistance (50% inhibition at 117 mg/L) to metronidazole was reported [124].

##### Quinoxalines

Quinoxalines are antibiotics containing an additional 2-(*N*-2′-hydroxyethylcarbamoyl) (present in olaquindox) or 2-cinnamoyl (present in quinocetone) groups.

Olaquindox, at 5 mg/L, caused 50% inhibition of *Microcystis aeruginosa* growth, and at 40 mg/L, inhibited growth of *Selenastrum capricornutum* by 50% [56]. Quinocetone, at 1.72 mg/L, caused 50% toxicity to *Pseudokirchneriella subcapitata* growth [48].

##### Ansamycins

Ansamycin antibiotics, such as rifamycin and rifampicin, possess a macrocyclic structure because they contain a naphthalene-related core spanned by an aliphatic ansa chain.

Rifamycin B at 9 mg/L (12 µM), rifamycin S at 4.17 mg/L (6 µM) and rifampicin at 9.87 mg/L (12 µM) completely inhibited growth of *Anacystis montana*. On the other hand, these antibiotics at higher concentrations (24 µM) did not affect growth of *Chlorella pyrenoidosa*, although cell bleaching was observed at antibiotic concentrations of 12 µM [125]. In another study, rifampicin at 171 mg/L caused 50% toxicity to *Pseudokirchneriella subcapitata* growth [48].

### 3.2. Nonsteroidal Anti-Inflammatory Drugs (NSAIDs)

NSAIDs are a class of drugs that include 2-(4-isobutylphenyl)propanoic acid (ibuprofen), 2-(6-methoxynaphthalen-2-yl)propanoic acid (naproxen), 2-[2-(2,6-dichloroanilino)phenyl]acetic acid (diclofenac), 2-(3-benzoylphenyl)propanoic acid (ketoprofen), 2-(1,8-diethyl-4,9-dihydro-3*H*-pyrano[3,4-b]indol-1-yl)acetic acid (etodolac), 2-acetyloxybenzoic acid (acetylsalicylic acid), and 2-hydroxybenzoic acid (salicylic acid) molecules and exert inhibitory effects on microalgal growth.

#### 3.2.1. Ibuprofen

Ibuprofen (IBU) was reported to inhibit the growth of various green microalgae. IBU, at 71–89 mg/L, inhibited growth of *Chlorella vulgaris* by 50% (48–96 h) [104], and at ≥0.1 g/L (0.5 mM), caused death of almost 100% *Chlorella* sp. cells [126]. For *Desmodesmus subspicatus,* IBU at 92 mg/L caused 50% inhibition in photosynthetic activity [84], and at 0.34 g/L, caused 50% inhibition in growth [127]. Furthermore, IBU caused 50% toxicity to *Pseudokirchneriella subcapitata* [128], *Acutodesmus obliquus*, *Chlamydomonas reinhardtii* and *Nannochloropsis limnetica* [129] growth at 0.23 [128], 0.288, 0.622 and 0.965 g/L, respectively [129].

Ibuprofen can also affect the growth of diatoms. Growth of *Navicula* sp. could be even completely suppressed in the presence of IBU at ≥50 mg/L, although the inhibitory effect was dependent on exposure time (2–10 days) [130].

#### 3.2.2. Naproxen

*Pseudokirchneriella subcapitata* growth was inhibited by 50% in the presence of 32–44.4 mg/L naproxen (NPX) [115,131]. NPX at 40–42 mg/L exerted 50% toxicity on *Chlorella vulgaris* or *Ankestrodesmus falcatus* growth [112]. Naproxen, at 100 mg/L, caused complete (100%) inhibition of *Scenedesmus quadricauda* growth (within 24 h) [132], but at 0.62 g/L, inhibited growth of *Desmodesmus subspicatus* by 50% [127]. For cyanobacteria, 50% inhibition of *Anabaena flosaquae* biomass was reported in the presence of 12.3 mg/L NPX [133].

#### 3.2.3. Diclofenac

Diclofenac (DCF), at 60–72 mg/L, caused 50% inhibition of *Desmodesmus subspicatus* growth [127,134], but at 147 mg/L, caused 50% inhibition of photosynthetic activity in *D. subspicatus* culture [84]. In another study, diclofenac, within a concentration range of 29–53 mg/L, caused 50% inhibition of *Haematococcus pluvialis* vegetative cell culture, and a negative effect on growth was pronounced with elongation of cultivation (up to 14 days) [135]. DCF, within 10–65 mg/L, caused 50% inhibition of *Pseudokirchneriella subcapitata* growth [82,115,136,137]; at 134 mg/L, caused 50% inhibition of *Chlamydomonas reinhardtii* population density growth [138]; at 60–150 mg/L, caused ≥30% inhibition of *Chlorella pyrenoidosa* biomass production/growth [107]; and at 185 mg/L, caused 50% toxicity to *Dunaliella tertiolecta* [139]. For other strains, DCF at 19.2 mg/L caused 50% toxicity to diatom *Cyclotella meneghiniana* growth [82] and at 14.5 mg/L, caused 50% toxicity to cyanobacterium *Synechococcus leopolensis* growth [82].

#### 3.2.4. Ketoprofen and Etodolac

Ketoprofen (KP) at 24.6-49 mg/L [115,116] and etodolac (ETD) at 102 mg/L [116] caused 50% inhibition of *Pseudokirchneriella subcapitata* growth.

#### 3.2.5. Acetylsalicylic Acid

Acetylsalicylic acid (ASA), at 106 mg/L, inhibited growth of *Desmodesmus subspicatus* by 50% [127], and at 241 mg/L, caused 50% inhibition of *Pseudokirchneriella subcapitata* growth [140]. Salicylic acid (SA) at 23.7 mg/L inhibited growth of *Pseudokirchneriella subcapitata* by 50% [141] and at 255 mg/L, caused 50% inhibition of *Phaeodactylum tricornutum* growth [90].

### 3.3. Fever and Pain Treatment Medicines

Paracetamol (acetaminophen) is an *N*-(4-hydroxyphenyl)acetamide medicine that has also been tested for its effect towards microalgal growth.

Acetaminophen, at concentrations only higher than 240 mg/L, caused inhibition of *Pseudokirchneriella subcapitata*, *Scenedesmus dimorphus*, *Stichococcus bacillaris*, *Chlorella vulgaris* and *Chlamydomonas reinhardtii* growth [142]. Accordingly, acetaminophen at 88.7 mg/L did not cause any inhibitory effect on *Pseudokirchneriella subcapitata* growth [116]. However, in other studies, acetaminophen at 88 mg/L [106] and 134 mg/L [143] reduced growth of *Chlorella vulgaris* [106] and *Scenedesmus subspicatus* [143] by 50%. In testing with a diatom species, paracetamol at 266 mg/L inhibited growth of *Phaeodactylum tricornutum* by 50% [90].

### 3.4. Antidepressants

Antidepressants are represented by fluoxetine, sertraline, duloxetine, clomipramine, paroxetine, amitriptyline, fluvoxamine, mianserine, citalopram, venlafaxine, milnacipran, trimipramine and sulpiride, which differ considerably in their structures. For example, fluoxetine is an *N*-methyl-3-phenyl-3-[4-(trifluoromethyl)phenoxy]propan-1-amine. Another antidepressant, sertraline, is a (1*S*,4*S*)-4-(3,4-dichlorophenyl)-*N*-methyl-1,2,3,4-tetrahydronaphthalen-1-amine. Furthermore, paroxetine is a (3*S*,4*R*)-3-(1,3-benzodioxol-5-yloxymethyl)-4-(4-fluorophenyl)piperidine. Other antidepressants also possess different structures. All these compounds were tested in terms of their effect on microalgal growth.

Fluoxetine, commonly present in medicines used in the treatment of depression [144], caused 50% toxicity/inhibition (Table 4, Table S2) to *Pseudokirchneriella subcapitata*/*Raphidocelis subcapitata* growth [115,145,146,147,148,149,150], different *Chlorella* strains [112,150,151] and *Scenedesmus* strains [150,151,152], *Chlamydomonas* strains growth [129,151], *Ankestrodesmus falcatus* growth [112], *Acutodesmus obliquus* [129] and *Dunaliella* strains [139,151]. For diatoms, fluoxetine caused 50% inhibition of *Skeletonema pseudocostatum* [153] and *Skeletonema marinoi* growth [147,148].

Except for fluoxetine, many other antidepressants can be harmful to microalgae. If compared to fluoxetine (50% inhibition at 0.2 mg/L), sertraline was slightly more inhibitory (0.15 mg/L), although other compounds, such as duloxetine (0.37 mg/L), clomipramine (0.46 mg/L), paroxetine (0.63 mg/L), amitriptyline (0.72 mg/L), fluvoxamine (0.98 mg/L), mianserine (2.1 mg/L), citalopram (3.3 mg/L), venlafaxine (47.5 mg/L) and milnacipran (61.3 mg/L), were less inhibitory towards *Pseudokirchneriella subcapitata* growth [115]. Further referring to fluoxetine (50% toxicity at 0.027 mg/L), sertraline (0.043 mg/L), fluvoxamine (0.062 mg/L), paroxetine (0.14 mg/L) and citalopram (1.6 mg/L) caused a weaker toxicity towards *Pseudokirchneriella subcapitata* growth [149]. Further, in comparison with fluoxetine (50% inhibition at 48 µg/L), other tested antidepressants were stronger (duloxetine at 1.9 µg/L, clomipramine at 3.3 µg/L), similar (amitriptyline at 43.8 µg/L) or weaker/much weaker (sertraline at 61.8 µg/L, fluvoxamine at 114.5 µg/L, paroxetine at 121 µg/L, citalopram at 500 µg/L, venlafaxine at 6900 µg/L) inhibitors of *Skeletonema marinoi* growth [148]. As compared to fluoxetine in a study with *Pseudokirchneriella subcapitata* (IC_50_ = 45 µg/L), *Scenedesmus acutus* (IC_50_ = 91 µg/L), *Scenedesmus quadricauda* (IC_50_ = 213 µg/L) and *Chlorella vulgaris* (IC_50_ = 4339 µg/L) tested, sertraline was a stronger or weaker inhibitor depending on the strain (with IC_50_ = 12 µg/L for *P.s.*, IC_50_ = 99 µg/L for *S.a.*, IC_50_ = 317 µg/L for *S.q.* and IC_50_ = 764 µg/L for *C.v.*) and fluvoxamine was the weakest inhibitor (with IC_50_ = 4 mg/L for *P.s.*, IC_50_ = 3.6 mg/L for *S.a.*, IC_50_ = 3.5 mg/L for *S.q.* and IC_50_ = 10.2 mg/L for *C.v.*) [150]. When compared to fluoxetine (50% toxicity within 24–207 µg/L), trimipramine (738–21,730 µg/L (1.8–53 µM)) was a much weaker toxicant of *Scenedesmus vacuolatus* growth [152].

Inhibitory concentrations of antidepressants can vary greatly between different strains tested [150]. However, in a study referring to sertraline (Sert)-HCl, growth of *Microcystis aeruginosa* and *Chlorella vulgaris* experienced quite similar inhibition (69% and 57%) in the presence of 0.200 mg/L Sert-HCl [154], and this was very comparable with another study where Sert-HCl at 0.14 mg/L inhibited *P. subcapitata* growth by 50% [155].

Another antidepressive agent, (±)sulpiride, at 99.8 mg/L caused 50% inhibition of *Pseudokirchneriella subcapitata* growth [116].

Metabolites of antidepressants can also affect microalgal growth. Norfluoxetine, a human metabolite of fluoxetine, at 242 µg/L caused 50% inhibition of *Pseudokirchneriella subcapitata* growth [146] and within 66–531 µg/L (0.2–1.6 µM), depending on pH, caused 50% toxicity to *Scenedesmus vacuolatus* growth [152].

### 3.5. Lipid Regulators

Lipid-lowering drugs are a diverse group of PHRs that include fibrates and statins. Fibrates are represented by 5-(2,5-dimethylphenoxy)-2,2-dimethylpentanoic acid (gemfibrozil), 2-(4-(4-chlorobenzoyl)phenoxy)-2-methylpropanoic acid 1-methylethyl ester (fenofibrate), 2-[4-[2-[(4-chlorobenzoyl)amino]ethyl]phenoxy]-2-methylpropanoic acid (bezafibrate) and 2-(4-chlorophenoxy)-2-methylpropanoic acid ethyl ester (clofibrate). Statins are another class of lipid-lowering medications represented by simvastatin. Fibrates and statins were reported to inhibit microalgal growth.

Gemfibrozil, fenofibrate and bezafibrate were tested on growth of *Pseudokirchneriella subcapitata*. Growth of *P.s.* was inhibited by 50% in the presence of 15–49 mg/L gemfibrozil [136,156,157] and 19.8 mg/L fenofibrate [156]. Bezafibrate at 103 mg/L [157] caused 50% inhibition of *Pseudokirchneriella subcapitata* growth. On the contrary, in other studies [116,156], no growth inhibition of *P.s.* was reported, even in the presence of 60–100 mg/L bezafibrate. Effects of gemfibrozil and bezafibrate were also tested on a cyanobacteria strain. It was reported that gemfibrozil and bezafibrate exerted 50% toxicity to *Anabaena* sp. culture at 4–9 and 7–41 mg/L, respectively, within 1–24 h of exposure [158].

Another fibrate, clofibrate, at 39.7 mg/L inhibited growth of *Tetraselmis chuii* by 50% [159].

Simvastatin, a representative of statins, at 22.8 mg/L caused 50% toxicity to *Dunaliella tertiolecta* [139].

Active metabolites of lipid regulators were also tested in terms of their effect on microalgal growth [82,137,139,143,158,159,160,161]. Clofibric acid, a metabolite of clofibrate, caused 50% inhibition/toxicity (Table 5, Table S2) to *Tetraselmis chuii* growth [159], *Scenedesmus subspicatus*/*Desmodesmus subspicatus* [143,160], *Pseudokirchneriella subcapitata* [82,137], *Dunaliella tertiolecta* [139], *Chlorella pyrenoidosa* [107] and cyanobacteria *Anabaena* sp. [158] and *Synechococcus leopolensis* growth [82].

### 3.6. Antineoplastic Agents

Anticancer drugs are represented by 5-fluorouracil (5-FU), cisplatin (CDDP), etoposide (ET), imatinib mesylate (IM), tamoxifen (TAM), methotrexate (MET), imatinib (IMT), cyclophosphamide (CP), ifosfamide (IF), thioguanine (TG), cytarabine (CytR) and gemcitabine (GemC), possessing vastly different structures. Antineoplastic drugs were tested in terms of their effect on microalgal growth.

A range of different antineoplastic agents (5-FU, CDDP, ET, IM, TAM, MET, IMT, CP, IF) were tested in terms of their inhibitory/toxic activity against *Pseudokirchneriella subcapitata*/*Raphidocelis subcapitata* growth, with 5-FU being the strongest inhibitor/toxicant. 5-Fluorouracil (within 0.075–0.96 mg/L), tamoxifen (>0.2 or 0.98 mg/L), cisplatin (1.52 mg/L), imatinib mesylate (2.29 mg/L), imatinib (5 mg/L), methotrexate (9.5 mg/L), etoposide (30.4 mg/L), cyclophosphamide (>100 mg/L) and ifosfamide (>100 mg/L) caused 50% inhibition/toxicity to *Pseudokirchneriella*/*Raphidocelis* growth [103,162,163,164]. The *Pseudokirchneriella*/*Raphidocelis* strain can be more/less sensitive to antineoplastic drugs than other green microalgal strains. Tamoxifen was more harmful to *Chlorella vulgaris* (IC_50_ = 0.61 mg/L) and *Chlamydomonas reinhardti* (IC_50_ = 0.47 mg/L) than to *Pseudokirchneriella subcapitata* (IC_50_ = 0.98 mg/L) [164]. On the other hand, 5-fluorouracil was much more harmful to *Raphidocelis subcapitata* than to *Scenedesmus obliquus* and *Desmodesmus quadricauda*, as 5-FU caused 50% toxicity to *Scenedesmus*/*Desmodesmus* growth at 30.5 and 20.5 mg/L, respectively [103]. Stronger resistance of *Scenedesmus*/*Desmodesmus* strains to antineoplastic drugs was also reported in other studies, where 5-fluorouracil (5-FU), cytarabine (CytR) and gemcitabine (GemC) exerted 50% toxicity on *Desmodesmus subspicatus* growth within 45–53 mg/L [165], and methotrexate at 260 mg/L caused 50% inhibition of *Scenedesmus subspicatus* growth [143].

For diatoms, thioguanine at 14.2 µg/L (8.5 × 10^−8^ mol/L) inhibited *Skeletonema pseudocostatum* growth by 50% [153].

In the case of studying the effect of 5-fluorouracil (5-FU), cisplatin (CDDP), etoposide (ET) and imatinib mesylate (IM) on the cyanobacterium *Synechococcus leopoliensis*, the strongest inhibitor was CDDP (causing 50% growth reduction at 0.67 mg/L), followed by 5-FU (1.2 mg/L) and IM (5.36 mg/L), with inhibitory activity of ET being not detected up to 351 mg/L [162]. For the cyanobacterium *Microcystis aeruginosa*, 50% toxicity was exerted by 5-FU at 6 mg/L [103].

Not only anticancer drugs, but also their byproducts can affect microalgal growth. Although cyclophosphamide (CP), at concentrations up to 320 mg/L, did not show toxicity to *Synechococcus leopoliensis*, its metabolite/transformation product, namely carboxy-cyclophosphamide (CPCOOH), was a much stronger toxicant, showing 50% toxicity at 17 mg/L [166].

### 3.7. Antiepileptic Agents

Anticonvulsants with antialgal activity are represented by 5*H*-dibenz(b,f)azepine-5-carboxamide (carbamazepine) and 5,5-diphenylimidazolidine-2,4-dione (phenytoin).

Carbamazepine (CBZ) was reported to be inhibitory to various microalgal strains (Table 6, Table S2). Growth of *Chlamydomonas mexicana*, *Chlamydomonas pitschmannii*, *Micractinium reisseri* and *Scenedesmus obliquus* was inhibited respectively by 23%, 31%, 43% and 60% (all at 100 mg/L CBZ) [167]. In other studies, carbamazepine caused 50% inhibition/toxicity to the growth of *Desmodesmus subspicatus* [160], *Scenedesmus obliquus* [168], *Chlorella pyrenoidosa* [168], *Chlorella vulgaris* [169], *Dunaliella tertiolecta* [170], the diatoms *Cyclotella meneghiniana* [82] and *Phaeodactylum tricornutum* [90], and the cyanobacterium *Synechococcus leopolensis* [82]. CBZ at 0.1–1 mg/L inhibited *Neochloris pseudoalveolaris* growth by 29% [171].

Phenytoin (PHT), at 28.3 mg/L, caused 50% inhibition of *Pseudokirchneriella subcapitata* growth [116].

### 3.8. Beta-Blockers

β-blockers (β-adrenergic antagonists) are represented by 1-(isopropylamino)-3-(1-naphthyloxy)propan-2-ol (propranolol), 1-(isopropylamino)-3-[4-(2-methoxyethyl)phenoxy]propan-2-ol (metoprolol), 1-p-carbamoylmethylphenoxy-3-isopropylamino-2-propanol (atenolol) and 1-(tert-butylamino)-3-((5,6,7,8-tetrahydro-cis-6,7-dihydroxy-1-naphthyl)oxy)propan-2-ol (nadolol), which possess inhibitory effect towards microalgae.

Amongst tested beta-blockers, propranolol (PRO) is reported to be the strongest inhibitor of microalgal growth. Propranolol caused inhibition/toxicity (Table 7, Table S2) to *Pseudokirchneriella subcapitata* growth [82,115,172], *Scenedesmus*/*Desmodesmus* culture/growth [84,152,160,173], *Acutodesmus obliquus* and *Chlamydomonas reinhardtii* [129] and *Chlorella vulgaris* growth [174]. PRO also caused 50% growth inhibition/toxicity to the diatoms *Skeletonema pseudocostatum* [153] *Cyclotella meneghiniana* [82] and *Phaeodactylum tricornutum* [90] and the cyanobacterium *Synechococcus leopolensis* [82].

Other beta-blockers were weaker inhibitors. Metoprolol (MET) at 7.3 mg/L caused 50% inhibition of *Desmodesmus subspicatus* growth [160] and at ~74 mg/L, 50% toxicity to *Scenedesmus vacuolatus* [173] and 50% inhibition of *Pseudokirchneriella subcapitata* growth [115]. Atenolol (ATE) caused 50% toxicity to *Pseudokirchneriella subcapitata* within 143–190 mg/L [175] and at 312 mg/L, inhibited growth of *Phaeodactylum tricornutum* by 50% [90]. Nadolol (NAD) was toxic to *Scenedesmus vacuolatus* growth at >100 mg/L [173].

### 3.9. Estrogens

Ethynylestradiol (EE2) and estradiol (E2), common representatives of estrogens, can inhibit growth of microalgae.

EE2 was reported to inhibit growth of different microalgal strains (Table 8, Table S2). EE2 caused 50% toxicity/inhibition of *Pseudokirchneriella subcapitata*/*Raphidocelis subcapitata* growth [103,176], *Scenedesmus*/*Desmodesmus* strains [84,103,176,177,178], *Chlorella vulgaris* growth [178] and *Dunaliella salina* growth [179], the diatom *Navicula incerta*’s growth [180] and the cyanobacteria *Microcystis aeruginosa* [103] and *Anabaena variabilis* [181].

Estradiol (E2) exerted a negative effect (Table 9, Table S2) on *P. subcapitata*/*R. subcapitata,* showing rather variable results, with 50% toxicity to *Pseudokirchneriella* growth at 0.01–0.87 mg/L [176] and a slight (15%) inhibition of *Raphidocelis* growth at 3.2 mg/L [182]. E2 also caused 50% toxicity/inhibiton of *Desmodesmus subspicatus* growth [176], *Chlorella vulgaris* and *Scenedesmus armatus* growth [178] and cyanobacterium *Anabaena variabilis* growth [181].

### 3.10. Other Drugs

#### 3.10.1. Anaesthetic Drugs

Lidocaine (LIDO), a 2-(diethylamino)-*N*-(2,6-dimethylphenyl)acetamide, inhibited growth of *Scenedesmus vacuolatus* within 12–1491 mg/L (0.05–6.3 mM) [152].

#### 3.10.2. Antianxiety Agents

Diazepam (DZP), a 7-chloro-1-methyl-5-phenyl-3*H*-1,4-benzodiazepin-2-one, caused 50% inhibition of *Tetraselmis chuii* growth at 16.5 mg/L [159].

#### 3.10.3. Proton Pump Inhibitors

Omeprazole (OMP), a 6-methoxy-2-[(4-methoxy-3,5-dimethylpyridin-2-yl)methylsulfinyl]-1*H*-benzimidazole, was reported to cause 50% inhibition of *Tetraselmis suecica* growth at 114 mg/L [183].

#### 3.10.4. Antiviral Drugs

Oseltamivir ethylester (OE), an ethyl (3*R*,4*R*,5*S*)-4-acetamido-5-amino-3-pentan-3-yloxycyclohexene-1-carboxylate, was tested in terms of its effect on microalgae. OE at 6.4 g/L (15.5 mM) inhibited photosynthetic activity by 50% in *Desmodesmus subspicatus* [184]. In another study, growth of *Pseudokirchneriella subcapitata* was inhibited by 50% in the presence of OE at 210–352 mg/L [184,185].

#### 3.10.5. Antiparasitic Drugs

Antiparasitic drugs (anthelmintics) with antialgal activity are represented by different compounds, such as methyl *N*-[6-(4-fluorobenzoyl)-1*H*-benzimidazol-2-yl]carbamate (flubendazole), methyl *N*-(6-phenylsulfanyl-1*H*-benzimidazol-2-yl)carbamate (fenbendazole) and a macrocyclic lactone derivative (abamectin).

Flubendazole (FLU) or fenbendazole (FEN), at concentrations above 1 mg/L, were reported to exert some toxicity towards *Scenedesmus vacuolatus* [186]. Abamectin (ABM), at 4.4 mg/L, caused 50% toxicity to *Scenedesmus subspicatus* growth [187].

#### 3.10.6. Antiallergic Agents

Antiallergic (antihistamine) agents such as 2-diphenylmethoxy-*N*,*N*-dimethylethylamine (diphenhydramine) and 3-amino-9,13b-dihydro-1*H*-dibenz(c,f)imidazo(1,5-a)azepine (epinastine) have shown antialgal activities.

Diphenhydramine (DPH) and epinastine (EPN) caused 50% inhibition of *Pseudokirchneriella subcapitata* growth at 1.24 and 3.75 mg/L, respectively [116].

#### 3.10.7. Angiotensin-Converting Enzyme (ACE) Inhibitors

Captopril (CPT), a (2*S*)-1-[(2*S*)-2-methyl-3-sulfanylpropanoyl]pyrrolidine-2-carboxylic acid, caused 50% inhibition of *Desmodesmus subspicatus* growth at 168 mg/L [160].

#### 3.10.8. Antiarrhythmic Agents

Procainamide (PA), a 4-amino-*N*-[2-(diethylamino)ethyl]benzamide, reduced growth of *Tetraselmis chuii* by 50% at 104 mg/L [70].

#### 3.10.9. Diuretics

Furosemide (FUR), a 4-chloro-2-[(furan-2-ylmethyl)amino]-5-sulfamoylbenzoic acid, did not show any effect on *Pseudokirchneriella subcapitata* growth at the highest concentration tested, 70 mg/L [188].

#### 3.10.10. Quinazolines

2-(4-Chlorophenyl)-4-(4-methoxyphenyl) quinazoline (CMQ), inhibited growth of *Microcystis aeruginosa* by 50% at 1.9 mg/L [189].

#### 3.10.11. Pleuromutilin Derivatives

Tiamulin (TI) is a derivative of the tricyclic diterpene antibiotic pleuromutilin in which the hydroxyacetate group is replaced by a 2-{[2-(diethylamino)ethyl]sulfanyl}acetate group. Tiamulin at 0.003 mg/L caused 50% inhibition of *Microcystis aeruginosa*, and at 0.165 mg/L, caused 50% inhibition of *Selenastrum capricornutum* growth [56].

### 3.11. Antiseptic/Preservative/Disinfectant Ingredients

The most common antiseptic/preservative/disinfectant agents are 5-chloro-2-(2,4-dichlorophenoxy)phenol (triclosan), 1-(4-chlorophenyl)-3-(3,4-dichlorophenyl)urea (triclocarban), trichloroisocyanuric acid, 1-(4-chlorophenoxy)-1-imidazol-1-yl-3,3-dimethylbutan-2-one (climbazole), a bromide salt of cetyltrimethylammonium (cetrimonium bromide) and esters of *p*-hydroxybenzoic acid (parabens), which exert negative effects on microalgal growth.

Triclosan (TCS) was inhibitory against growth of numerous green microalgae (Table 10, Table S2). Triclosan caused inhibition/toxicity to *Pseudokirchneriella subcapitata/Raphidocelis subcapitata/Selenastrum capricornutum* growth [81,157,190,191,192,193], different *Chlamydomonas* strains [151,194,195], *Dunaliella* strains [139,151], *Tetraselmis suecica* [196], different *Scenedesmus*/*Desmodesmus* strains [151,197,198,199] and *Chlorella* strains [151,199,200]. Inhibitory/toxicity effects of TCS on growth of the diatoms *Skeletonema pseudocostatum* [153], *Navicula* sp. [201] and another diatom strain [202] and the cyanobacteria *Microcystis aeruginosa* [203] and *Anabaena flosaquae* [197] were also reported.

Other antiseptics can also inhibit, to different extents, the growth of microalgae. Triclocarban (TCC) at 5.7–17 µg/L caused 50% inhibition of *Pseudokirchneriella subcapitata* growth [81,190]. Trichloroisocyanuric acid (TCCA) at 0.12–0.313 mg/L inhibited growth of *Chlorella vulgaris* by 50% [105,106]. Climbazole, at 2 mg/L, caused 50% inhibition of *Scenedesmus obliquus* growth [204]. Cetrimonium bromide, at 1.64 mg/L (4.5 × 10^−6^ mol/L), caused 50% inhibition of *Skeletonema pseudocostatum* growth and was 60 times less inhibitory than triclosan [153].

Amongst tested parabens, benzylparaben was the strongest toxicant, causing 50% inhibition of *Pseudokirchneriella subcapitata* growth at 1.2 mg/L, followed by *i*-butylparaben (3.3-fold lower toxicity than benzylparaben) and *n*-butylparaben (7.9-fold lower toxicity than benzylparaben) [205]. In another study, different parabens inhibited growth of *P. subcapitata* by 50% already at 0.17 mg/L (propylparaben), 0.1 mg/L (butylparaben) and 0.4 mg/L (ethylparaben) [140]. In one more study, methylparaben at 35 mg/L caused 50% inhibition of *P. subcapitata* growth [191].

### 3.12. General Inhibitory/Toxicity Ranges of Pharmaceuticals (PHRs) and Personal Care Products (PCPs) towards Different Microalgal Groups

Table 11 shows that PHRs and PCPs can significantly inhibit growth of microalgae and cyanobacteria at concentrations from μg/L to g/L, although these concentrations can vary greatly depending on the strains and toxicants tested. The most remarkable difference was reported for β-lactams, which strongly inhibited most cyanobacterial strains tested (except for *Nostoc*) at very low concentrations (μg/L), but were much less or not harmful to green microalgae such as *Chlorella vulgaris* or *Pseudokirchneriella subcapitata* (even at up to 2 g/L for *Ps. sub.* in some reports), although *Scenedesmus* was reported to be sensitive to cephalosporin (cefradine) at mg/L concentrations. Cyanobacteria were also reported to be more sensitive to quinolones (enrofloxacin, norfloxacin, levofloxacin, ciprofloxacin, sarafloxacin, flumequine, oxolinic acid) than green microalgae. For phenicols, *Chlorella* strains were less sensitive to thiamphenicol than other microalgae and all cyanobacteria strains. Chloramphenicol and florphenicol were more inhibitory to the growth of eukaryotic microalgae than thiamphenicol. For tetracyclines, cyanobacteria strains can be inhibited at lower antibiotic concentrations than green microalgae. However, there are separate literature reports showing *Pseudokirchneriella subcapitata* to be more sensitive to tetracyclines (TET, OXY) than cyanobacteria, although contrary data (showing *Microcystis* to be more sensitive to OXY than *Selenastrum*) are also present. Aminoglycosides are more harmful to cyanobacteria (especially *Microcystis aeruginosa*) than green microalgae, and streptomycin is the strongest inhibitor. Many different sulphonamide antibiotics were tested mostly on green microalgae, with some reports on other microalgae and cyanobacteria. Based on data gathered, sulfadimethoxine could be more harmful to green microalgae than cyanobacteria. Regarding the negative effect of trimethoprim, green microalgae and cyanobacteria seem to be similarly affected, if different literature data are considered. Macrolides begin to inhibit the growth of both green microalgae and cyanobacteria at very low concentrations and within the same general range upwards. However, the difference in the inhibitory effect of clarithromycin (macrolide) on *Pseudokirchneriella subcapitata* growth can be as much as 100 times between different studies (see Section 3.1.8). Regarding lincosamide antibiotics, cyanobacteria were more sensitive to lincomycin than diatoms and/or green microalgae. However, clindamycin could be more inhibitory to *Pseudokirchneriella subcapitata* than lincomycin. Antibiotics such as olaquindox and ansamycins were more inhibitory to cyanobacteria than green microalgae. Metronidazole, a nitroimidazole antibiotic, showed inhibitory/toxic activity to various green microalgal strains within 7.8 μg/L–705 mg/L. Tiamulin was much more harmful to cyanobacteria than green microalgae.

Cyanobacteria are susceptible to antibiotics, but the strain susceptibity can be very specific. *Spirulina platensis* was reported to be sensitive to hygromycin (HM) and chloramphenicol, but highly resistant to kanamycin, neomycin, geneticin and ampicillin [206].

Amongst NSAIDs, growth of green microalgae was inhibited by ibuprofen within 71–622 mg/L, naproxen within 32–620 mg/L and diclofenac within 10–185 mg/L. Acetaminophen, a fever and pain treatment medicine, showed rather moderate inhibitory activity towards growth of green microalgal strains.

For antidepressants, fluoxetine significantly inhibited growth of green microalgae from 24 µg/L upwards. Other representatives of antidepressants were weaker or stronger inhibitors of green microalgal growth. Regarding lipid regulators, gemfibrozil and fenofibrate were more inhibitory to *Pseudokirchneriella subcapitata* growth than bezafibrate. Gemfibrozil was rather more inhibitory to cyanobacterium *Anabaena* sp. than bezafibrate. Clofibrate can be more inhibitory to green microalgae than its metabolite, clofibric acid.

Antineoplastic agents, depending on structure, inhibited growth of different microalgae at various concentrations. 5-fluorouracil was more inhibitory to *Pseudokirchneriella subcapitata/Raphidocelis subcapitata* than to other green microalgae or even cyanobacteria. Separate studies show that etoposide can be less inhibitory to *Pseudokirchneriella subcapitata* and cause no harm to *Synechococcus leopoliensis* growth. Amongst green microalgae, *Scenedesmus*/*Desmodesmus* strains seem to be less sensitive to antineoplastic drugs.

Carbamazepine, an antiepileptic agent, showed rather moderate inhibitory/toxic activity towards the growth of green microalgae, although *Neochloris pseudoalveolaris* could be more sensitive. Amongst beta-blockers tested on the growth of green microalgae, propranolol was the strongest inhibitor, followed by metoprolol and nadolol. Green microalgae can be more sensitive to estrogens than cyanobacteria or diatoms.

For disinfectants, triclosan was reported to be very toxic/inhibitory to some green microalgae (*Pseudokirchneriella subcapitata*/*Raphidocelis subcapitata*) as much as to cyanobacteria. Nevertheless, toxicity/inhibition concentrations can vary greatly (0.53 µg/L–4 mg/L) between different green microalgal strains. Other disinfectants were harmful to the growth of green microalgae within 5.7 µg/L–35 mg/L.

Regarding other pharmaceuticals, such as other antibiotics (glycopeptides, nitrofurans), anesthetic drugs, antianxiety agents, proton pump inhibitors, antiviral drugs, antiparasitic drugs, antiallergic agents, ACE inhibitors and antiarrhythmic agents, the inhibitory/toxic concentration data are rather too scarce to reach any detailed conclusions, but inhibitory/toxic concentrations ranged from 0.5 mg/L (nitrofurans) to 6.4 g/L (oseltamivir ethylester).

## 4. Stimulatory Effect of PHRs on Microalgal Growth

Despite the inhibitory/toxic activity of PHRs towards microalgae, several reports suggest that a stimulatory effect of PHRs on microalgal growth is also possible. The use of some pharmaceuticals (paracetamol, salicylic acid, diclofenac) as carbon sources by *Chlorella* strains (with growth improvement up to 43%) was reported to be possible [207,208]. *Phaeodactylum tricornutum* was reported to use bezafibrate (60 μg/L) as a carbon source to mixotrophically increase cell densitycompared to a photoautotrophic control [209]. Further, growth of cyanobacterium *Synechocystis* sp. was stimulated by even up to 72% in the presence of 10 µg/L–1 mg/L ibuprofen [62]. Some antibiotics at low concentrations were also reported to stimulate cyanobacterial growth. Amoxicillin, at 100–300 ng/L, had a stimulatory effect on *Microcystis aeruginosa* growth [210]. In another study, amoxicillin at 200–500 ng/L did not alter or slightly enhanced the growth of *Microcystis aeruginosa*, and the stimulatory effect was correlated with the increase in N concentration (from 0.05 to 5 mg/L) in the growth medium [211]. Moreover, growth of *Microcystis aeruginosa* was improved by up to 33% in the presence of 50–200 ng/L of ciprofloxacin [212] and by up to 25% in the presence of 100–200 ng/L of sulphamethoxazole [212]. Interestingly, the cyanobacterium *Phormidium valderianum* was able to utilize ampicillin as a nitrogen source via extracellular production of β-lactamase, the enzyme that breaks a β-lactam ring [213]. Degradation products of minocycline were reported to slightly (~20%) enchance *Microcystis aeruginosa* growth [72]. A stimulatory effect of antibiotics on the growth of green microalgae and diatoms has also been reported. Photodegradation products of sulfacetamide and sulfamethoxazole stimulated (even by ~90%) the growth of *Chlorella vulgaris* [79]. *Chlorella* biomass production, cultivated on tilmicosin-containing wastewater and with a supply of 15% CO_2_, was improved by up to 18% at a low (0.1 mg/L) tilmicosin concentration [214]. Growth of *Skeletonema costatum* was stimulated at lower (0.5 to 2 mg/L) florfenicol concentrations [53]. Estrogens, at small concentrations, were also mentioned to stimulate microalgal growth. Ethinylestradiol within 0.01–2 mg/L was reported to slightly enhance *Chlorella* biomass production, with maximal stimulation (~15%) at 0.01 mg/L [215]. Growth of *Dunaliella salina* was also slightly stimulated at 10 ng/L of EE2 [179]. Regarding anticancer agents, tamoxifen at 10 μg/L was reported to stimulate *Selenastrum capricornutum* growth [24].

## 5. Effect of Pharmaceuticals and Personal Care Products on Microalgal Metabolism

PHRs- and PCPs-related compounds can affect the growth of microalgae (eukaryotic microalgae and cyanobacteria) and induce changes in microalgal cellular metabolism. Upon exposure to PHRs and PCPs, oxidative stress appears in exposed microalgal cells, and reactive oxygen species (ROS) generated affect the synthesis of biomolecules such as pigments, lipids and proteins.

### 5.1. Antibacterial Activity of Antibiotics

Antibiotics are widely reported to act on the metabolism of prokaryotic cells in a very specific mode. Aminoglycosides interfere with bacterial protein synthesis by binding to the 30S subunit of the bacterial ribosome [56], thereby causing the production of mistranslated proteins and consequently membrane damage and death [216]. Tetracyclines bind to the bacterial 30S ribosomal subunit, thereby preventing the binding of aminoacyl tRNAs to the A site of the ribosome and consequetly inhibiting protein synthesis [42]. Quinolones interfere with DNA replication by inhibiting DNA gyrase [105,216] and topoisomerase IV [216] in *Prokaryota*. Phenicols bind to the bacterial 50S ribosomal subunit, thereby inhibiting the activity of peptidyl transferase and consequently inhibiting the synthesis of proteins [217]. Macrolides and lincosamides also bind to the 50S ribosomal subunit and block the path by which nascent peptides exit the ribosome [218]. Sulfonamides inhibit dihydropteroate synthase, thereby interfering with the conversion of *p*-aminobenzoic acid for folic acid synthesis and consequently inhibiting DNA synthesis [45,81]. Trimethoprim inhibits dihydrofolate reductase, thereby interfering with the conversion of dihydrofolic acid to tetrahydrofolic acid and consequently inhibiting DNA synthesis [45,81]. Glycopeptide antibiotics have an inhibitory mode of action on peptidoglycan synthesis in the bacterial cell wall [75]. Beta-lactams are reported to cause malfunctions of the machinery for peptidoglycan cell wall biosynthesis [216]. Besides having antibacterial activity, antibiotics can also exert adverse effects on eukaryotic aquatic microorganisms such as microalgae.

### 5.2. Lipid Peroxidation

Lipid peroxidation occurs during cellular oxidative stress, leading to the generation of malondialdehyde (MDA), a peroxidation product of fatty acids. Table 12 shows that PHRs such as amoxicillin [219,220,221], ciprofloxacin [108,222], enrofloxacin [95,97], norfloxacin [99,219], levofloxacin [102], tetracycline [57], doxycycline [71], chlortetracycline [64], florfenicol [46], thiamphenicol [46], erythromycin [113,222], spiramycin [220], sulfamethoxazole [222], carbamazepine [170] and ethinylestradiol [179] and PCPs such as triclosan [194,195] can cause increases in MDA content in different microalgal strains. The general pattern in mentioned reports is that MDA content increases up to the maximal concentration with the increase in PHRs/PCPs concentrations in the media. The maximal increase in MDA can range from an 1.18-fold to a 25.6-fold increase, depending on the report (Table 12). The concentrations of PHRs/PCPs, inducing MDA production, can differ from ng/L to mg/L in the cultivation media. Some reports also show that further increasing toxicant concentration can decrease MDA content in microalgae, if compared to the maximal MDA content [46,97]. Synthesis of MDA in microalgae exposed to different PHRs/PCPs can be additionally influenced by changing the exposure time [170,195,220], applying a second exposure step [57,64], modifying N [211] or P [221] concentrations in the media or adding an intracellular Ca^2+^ chelator into the culture [195]. The increase of MDA content is a universal marker for the occurrence of oxidative stress in microalgae exposed to PHRs and PCPs.

### 5.3. Antioxidant Enzymes

Appearance of oxidative stress affects production of antioxidant enzymes, such as superoxide dismutase (SOD), catalase (CAT) and peroxidase (POD), which serves as a protection mechanism against ROS.

Antibiotics were widely reported to modulate activities of antioxidant enzymes in microalgal cells (Table 13). Antioxidant response in *Microcystis* strains towards different antibiotics was a topic of many reports. For instance, erythromycin [113], florfenicol and thiamphenicol [46] increased activities of SOD and CAT in *Microcystis flosaquae* cells [46,113]. Further, the exposure of *Microcystis aeruginosa* to tetracycline [57] and chlortetracycline [64] resulted in the increased activity of SOD. CAT activity increased while SOD activity remained unchanged in *Microcystis aeruginosa* exposed to doxycycline [71]. Although the antioxidant activity of enzymes can increase, these activities have also been reported to decrease. In one report [69] where *Microcystis aeruginosa* cells were treated with chlortetracycline (0.05 mg/L), SOD activity decreased, but POD and CAT activities increased [69]. In another report [220], where *Microcystis aeruginosa* cells were treated with spiramycin, SOD activity increased, but POD and CAT activities decreased. The SOD activity also decreased in *Microcystis aeruginosa* exposed to norfloxacin [219]. Results for *Microcystis aeruginosa* exposed to chlortetracycline show that SOD activity can increase [64] or decrease [69]. Results for another antibiotic also show that SOD activity decreased in *Microcystis aeruginosa* exposed to amoxicillin [219], but in another report with amoxicillin treatment, all activities (SOD, POD, CAT) increased [220]. Such discrepancies could possibly occur due to several factors, such as different antibiotic concentrations, different exposure time and imbalance in antioxidant activity and ROS production.

Green microalgae were also tested in terms of their antioxidant response to antibiotic treatment (Table 13). The exposure of *Selenastrum capricornutum* to tetracycline [57], *Chlorella pyrenoidosa* to chlortetracycline [64] and *Chlamydomonas mexicana* to ciprofloxacin [108] resulted in the increased activity of SOD in cells of these strains. For another study, erythromycin decreased while ciprofloxacin or sulfamethoxazole increased the activities of CAT, SOD, GPX and glutathione-*S*-transferase (GST) in *Pseudokirchneriella subcapitata* cells at the highest antibiotic concentration tested [222].

Except for antibiotics, other PHRs (carbamazepine, fluoxetine and ethinylestradiol) and PCPs (triclosan) can also alter antioxidant enzyme activity (Table 13). Carbamazepine (CBZ) caused an increase in CAT activity and SOD activity in *Scenedesmus obliquus* and *Chlorella pyrenoidosa* cells [168]. In another study, the exposure of *Scenedesmus obliquus* to increasing CBZ concentrations resulted in a decrease in SOD activity and increase in CAT activity [167]. Moreover, the exposure to CBZ led to increased or decreased (at higher CBZ concentration) activity of SOD in *Chlamydomonas mexicana* cells [167]. In *Chlorella vulgaris* and *Ankestrodesmus falcatus* cells exposed to fluoxetine, activities of SOD and CAT decreased [112]. The exposure of *Navicula incerta* to ethynylestradiol (EE2) caused an increase in SOD activity and a decrease in POD activity in those cells [180]. Similarly, SOD activity increased while CAT and glutathione peroxidase (GPX) activity decreased in *Dunaliella salina* cells exposed to ethinylestradiol [179]. In *Microcystis aeruginosa* cells exposed to triclosan, SOD activity increased and subsequently sharply decreased, but POD activity remained unchanged compared to the control [203].

Alterations in SOD, CAT and POD activities can constitute biomarkers for oxidative stress in microalgae exposed to increasing concentrations of PHRs/PCPs.

### 5.4. Pigments

Content and composition of pigments in microalgal cells can change in the presence of PHRs and PCPs. Exposure to carbamazepine (CBZ) at 50 mg/L led to an increase in chlorophyll (by 19%) and carotenoid (by 25%) content in *Chlamydomonas mexicana* cells [167]. In another study, CBZ within 10–200 mg/L resulted in increased (up to 5-fold) content of carotenoids and increased/decreased (depending on cultivation time) content of chlorophyll in *Dunaliella tertiolecta* cells [170]. Studying the effect of enrofloxacin (ENR) at up to 100 mg/L on microalgal strains, it was shown that the exposure to ENR caused various changes (increase, decrease) in pigment (*Chl*, *Car*) content in cells of *Scenedesmus obliquus* (*Chl* decrease, *Car* increase), *Chlorella vulgaris* (*Chl* decrease, *Car* decrease), *Chlamydomonas mexicana* (*Chl* increase, *Car* increase), *Ourococcus multisporus* (*Chl* decrease, *Car* increase) and *Micractinium resseri* (*Chl* increase, *Car* increase) [95]. In another study, exposure of *Scenedesmus quadricauda* cells to 3.4 mg/L (25 µM) of salicylic acid resulted in a 100% increase in Chl *b* content in *Scenedesmus* cells [223]. On the other hand, Chl *b* content decreased (by 27%) and Car content increased (by 43%) in *Chlamydomonas reinhardtii* cells treated with diclofenac (at 134 mg/L) [138]. The exposure to amoxicillin resulted in a ~22% decrease in chlorophyll *a* and carotenoid content in *Microcystis aeruginosa* biomass [224]. The content of Chl *a* in *Selenastrum capricornutum* cells decreased upon treatment with erythromycin (up to 0.3 mg/L), ciprofloxacin (up to 2.5 mg/L) or sulfamethoxazole (up to 2.5 mg/L), with the biggest drop (by up to ~50%) for erythromycin treatment (at 0.3 mg/L) [80]. A long-term exposure (28 days) of *Scenedesmus rubescens* to 1 mg/L ibuprofen caused a change in *Scenedesmus* metabolism, with a decrease (by 60%) in Chl content and increase (by 22%) in Car content, although Car/Chl ratios varied depending on cultivation time (7 to 28 days) [225]. The exposure of *Dunaliella salina* to 1 μg/L ethinylestradiol (EE2) resulted in a ~6-fold drop in chlorophyll (*a* and *b*) content in *Dunaliella* cells [179]. Moreover, carotenoid content in *Dunaliella* cells initially increased and subsequently dropped with further increase (up to 1 μg/L) in EE2 concentration [179]. In other studies, exposure of diatom cells to triclosan (0.32 mg/L, 0.4 mg/L) resulted in a decrease in cellular chlorophyll *a* (by 66% and 75%) [201,202] and carotenoid content (by ~70%) [202]. Chlorophyll *a*/*b* ratio could be also decreased (20% increase in Chl *b*) in *Pseudokirchneriella subcapitata* cells treated with drugs [226]. Moreover, a decrease (by up to 50%) in carotenoid content and a drop to zero in astaxanthin content was observed in *Haematococcus pluvialis* cells exposed to diclofenac (at 75–100 mg/L) [135].

### 5.5. Toxins

Antibiotics were reported to possess various effects on cyanobacterial toxin synthesis. Amoxicillin (at 100 ng/L) increased the microcystin (MC) cell content in *Microcystis aeruginosa* and its extracellular release. Moreover, the exposure to amoxicillin induced changes in the protein pattern in *Microcystis* cells [210]. On the contrary, exposure to tetracycline (at 1–10 mg/L) resulted in the decrease of microcystin-LR (MC-LR) production by *M. aeruginosa* [60]. Further, the production of MC-LR in *Microcystis aeruginosa* was inhibited by spiramycin (at 800 ng/L), but stimulated in the presence of amoxicillin (at 800 ng/L) [227]. Furthermore, spiramycin was reported to stimulate or suppress microcystin production and release by *M. aeruginosa*, depending on spiramycin and nitrogen concentrations [228], via regulation of microcystin synthetases and ATP binding cassette (ABC) transporters [229].

### 5.6. Other Toxicity Endpoints

The exposure to pharmaceuticals and other related compounds can affect the whole microalgal cell. Ofloxacin was reported to inhibit photosynthetic activities of photosystem I (PSI) and photosystem II (PSII) in *Microcystis aeruginosa* cells [230], while diclofenac affected a fraction of the PSII reaction centers in *Chlamydomonas reinhardtii* cells [138] and β-estradiol affected energy fluxes at the photosystem II level in *Microcystis aeruginosa* [231]. Rifampicin was reported to block phycocyanin and chlorophyll *a* light acclimation in *Anacystis nidulans* [232]. Treatment of *Selenastrum capricornutum* with erythromycin resulted in the decrease in cyclic and noncyclic photophosphorylation activity, Mg^2+^-ATPase activity and ribulose-1,5-bisphosphate carboxylase (RuBPCase) activity in *Selenastrum* cells [233]. Exposure to antibiotics (sulfamethoxazole, clarithromycin, ciprofloxacin, erythromycin) caused an increase in energy consumption in *Raphidocelis subcapitata* cells, resulting in a decrease in cellular energy allocation [234]. Exposure of *Tetraselmis suecica* cells to omeprazole (OMP) caused intracellular ROS generation, intracellular acidification of cytoplasm, hyperpolarization of cytoplasmic and mitochondrial membranes, an increase in cell size and a decrease in cellular chlorophyll *a* content [183]. Nystatin caused the leakage of potassium ions from *Chlorella vulgaris* cells [119]. Exposure to 5-fluorouracil led to the decrease in ascorbic acid (AsA) content in *Scenedesmus quadricauda* cells [235]. Treatment of *Nostoc flagelliforme* with chloromycetin stimulated exopolysaccharide production [44]. Exposure to ethynylestradiol resulted in the decrease in polysaccharide and total protein content and increase in lipid content in *Navicula incerta* cells [180]. Carbamazepine was reported to affect phospholipid bilayer and protein structures in *Chlorococcum* sp. cells [236] and cause a reduction in sugar content in *Parachlorella* and *Neochloris* cells [171]. Changes in the composition and structure of lipids, proteins and DNA were also found in microalgal cells exposed to chloramphenicol and roxithromycin [40]. Carbenicillin and lincomycin caused the decomposition of nucleoids in exponentially growing *Synechocystis* cells [237]. Rifampicin and chloramphenicol inhibited the development of proheterocysts and mature heterocysts in *Anabaena cylindrica* [238]. The exposure to norfloxacin, amoxicillin or cefradine caused a decrease in *Microcystis aeruginosa* cell size to different extents [219]. On the contrary, exposure to florfenicol caused an increase in *Skeletonema costatum* cell size [53].

A range of alterations in microalgal cells was found upon exposure to triclosan. Triclosan induced changes in *Tetraselmis suecica* cells, with a decrease in esterase activity and chlorophyll *a* fluorescence and alterations in cytoplasmic membrane properties being detected [196]. Further modifications in the metabolism of *Chlamydomonas* cells exposed to triclosan were: alterations in intracellular pH, a change in protein pattern, inhibition of esterase activity and increase in activity of caspase 3/7, a protein involved in programmed cell death [195]. Moreover, structural alterations related to lipids, protein phosphorylation, amide I and carbohydrates were detected in *Chlamydomonas reinhardtii* cells cultivated in the presence of triclosan [194]. Further, the exposure to triclosan resulted in the disappearance of chloroplast membrane, the spilling out of the inner stroma, vacuolation of the cytoplasm and overall damage of the cell wall in *Chlorella pyrenoidosa* cells [239]. Furthermore, exposure to triclosan caused changes in fatty acids/lipids and protein structure in *Chlorococcum* sp. cells [236,240]. In *Microcystis aeruginosa* cells exposed to triclosan, the appearance of polyphosphate bodies and lipid bodies was observed [203].

## 6. Interactions between Microalgal Cells and PHRs/PCPs in the Presence of Organic/Inorganic Matter

The interactions between microalgal cells and PHRs/PCPs molecules can be altered in the presence of organic or inorganic matter. For instance, addition of folic acid alleviated the inhibitory effect of sulphonamide on microalgal growth [45]. For other organic matter, the presence of humic acid at relatively low concentrations (up to 20 mg/L) could nullify the negative effect of triclosan on diatom strain growth. However, at higher humic acid concentrations (30–50 mg/L), diatom species growth was significantly reduced, suggesting the synergistic negative effect of humic acid and triclosan on a tested diatom strain [202]. Another study reports that the inhibitory effect of erythromycin or clarithromycin on *Pseudokirchneriella subcapitata* growth was increased in the presence of fungicide ketoconazole, thereby showing synergism between antibiotics and fungicides [110]. Inorganics can also alter PHRs/PCPs–cell interactions. The combined effect of a metal ion (Cu^2+^) and antibiotic (chlortetracycline) on *Chlorella pyrenoidosa* and *Microcystis aeruginosa* growth and metabolism was different to their individual effects [64]. Salinity can be also a factor affecting algal cell–PHRs/PCPs interactions. It was reported that a 3-fold increase in salinity caused a 5.4-fold decrease in the toxicity of sulfadimethoxine (SDM) towards *Chlorella vulgaris*. The reason for this could be that monovalent cations from the surroundings bind to countercharged groups on microalgal surfaces, thereby creating a barrier to the contact between PHR molecules and the microalgal cell [78]. Furthermore, resistance of *Chlorococcum* sp. to triclosan could be improved if phosphorous concentration was elevated [236,240]. A phosphorous level is an important factor, as at limited P concentrations, the presence of pharmaceuticals (fluoxetine, ibuprofen or propranolol) caused an increase in the carotenoid/chlorophyll ratio in *Nannochloropsis limnetica* cells [241]. Additionally, under P-limited conditions, the sensitivity of microalgal strains (*Acutodesmus obliquus*, *Chlamydomonas reinhardtii*) to fluoxetine and propranolol was alleviated, but sensitivity to ibuprofen was enhanced [129]. Pollutants such as microplastics can influence the effect of pharmaceuticals on microalgae. It was reported that the presence of microplastics increased the sensitivity of *Tetraselmis* to doxycycline (DOXY) by 100% [70]. Nanoparticles can modulate the effect of pharmaceuticals on microalgal growth. It was reported that CeO_2_ nanoparticles (NPs) alleviated the toxic effect of erythromycin on *Chlamydomonas reinhardtii* and *Phaeodactylum tricornutum* growth [111], or the toxic effect of florfenicol on *Chlorella pyrenoidosa* growth [51], by limiting the contact/interaction between antibiotics and microalgal cells. Pharmaceuticals themselves can also influence interactions between microalgae and particles. The presence of tetracycline enhanced the inhibitory effect of TiO_2_ nanoparticles (NPs) on *Scenedesmus obliquus* growth and could also modulate the excretion of exopolymeric substances from *Scenedesmus* cells [59]. Anticancer drugs can also possess antialgal activity when combined in a form of nanotherapeutics. Paclitaxel, in a free form and at 0.2 mg/L, did not inhibit growth of *Raphidocelis subcapitata* and *Chlamydomonas reinhardtii*. However, paclitaxel-loaded nanomedicine inhibited growth of *Raphidocelis* and *Chlamydomonas* by 50% at 1.1 mg paclitaxel/L and 1.6 mg paclitaxel/L, respectively, probably due to the abilities of nanoparticles to increase pharmaceutics’ solubility and to enhance the contact between pharmaceutical molecules and microalgal cell surfaces [242].

## 7. Removal of PHRs/PCPs During Microalgal Cultivation

PHRs and PCPs, as pollutants, can cause a threat to the environment and methods for their removal are drawing increasing attention. The use of microorganisms, such as bacteria, fungi and microalgae, to remove pharmaceutical pollutants can be a green approach to preventing environmental pollution [10,18,21].

### 7.1. General Mechanisms of PHRs/PCPs Removal

Mechanism of pollutant removal can work through different ways. Firstly, pollutants can adsorb on microalgal biomass due to interactions between specific functional moieties in the pollutant structure and groups present on the microalgal cell surface [10]. Secondly, pollutants can be accumulated/absorbed inside cells through diffusion or active transport [85]. Bioaccumulated pollutants can undergo transformation or partial degradation via metabolic pathways with the generation of byproducts/intermediates [130,132,201,202]. Degradation of chemical pollutants by extracellular polymeric substances (EPSs) released from microalgal cells is also possible [18].

### 7.2. Microalgal Cultures for Successful PHRs/PCPs Removal

Successful removal of PHRs and PCPs from prepared wastewaters was reported in numerous studies. A microalgal consortium dominated by *Dictyosphaerium* was reported to efficiently remove a range of pharmaceuticals, including beta-blockers (atenolol, bisoprolol, metoprolol), antibiotics (clarithromycin), antidepressants (bupropion) and other drugs (terbutaline, diltiazem), during wastewater treatment [243]. A complete removal of ibuprofen was achieved with the use of microalgal cultures dominated by *Chlorella* and *Scenedesmus* strains [244], and a complete removal of tilmicosin from wastewater was achieved in a *Chlorella* culture [214]. Furthermore, an 88% removal of climbazole was achieved in a *Scenedesmus obliquus* culture [204], a 93% removal of ceftazidime in a *Chlorella pyrenoidosa* culture [245], a 100% removal of triclosan in *Nannochloris* sp. culture [246] and a 100% removal of triclosan in *Scenedesmus obliquus* and *Desmodesmus* cultures, respectively [199]. Despite reports on the successful removal of PHRs/PCPs by microalgal cultures, this approach faces a series of limitations, such as insufficient and selected PHRs/PCPs removal, inhibition of microalgal growth and feasibility of transferring a lab study into the industrial scale.

### 7.3. Limitations for PHRs/PCPs Removal: Insufficient and Specific Microalgal PHRs/PCPs Removal

Microalgal cultures are capable of bioremediating some PHRs/PCPs, but fail to remove others. Carbamazepine almost completely remained in the medium during treatment with a microalgal culture (with *Chlorella* and *Scenedesmus* strains) [244]. Low removal rates of levofloxacin in *Scenedesmus obliquus* (4.5%) [101] and *Chlorella vulgaris* (9.5%) [100] cultures, enrofloxacin in different microalgal cultures containing *Chlamydomonas mexicana*, *Scenedesmus obliquus*, *Chlorella vulgaris*, *Ourococcus multisporus* or *Micractinium resseri* (25%, 23%, 26%, 18% and 20%, respectively) [95] and sulfamethoxazole in *Scenedesmus obliquus* (up to 30%) [77] were also reported. Besides low removal rates, strain culture specificity for a particular PHR’s removal can also appear. From different microalgal strains (*Chlorella*, *Desmodesmus*, *Scenedesmus*, *Coelastrella*, *Coelastrum*) tested for the removal of 19 pharmaceuticals, it was shown that a vast difference in the removal of PHRs such as trihexyphenidyl (44–100%), amitriptyline (42–100%), ofloxacin (49–100%), trimethoprim (3–48%) and carbamazepine (19–75%) could be seen amongst tested cultures [247]. Accumulation of PHRs in biomass also differred greatly, as accumulation rates of trihexylphenidyl and clomipramine in biomass ranged from 0% to 100%, depending on the strain used [247]. It should be also expected that the lower removal rate can be obtained due to the desorption of the pollutant from biomass, which can depend on the pH value and ionic presence [248] and can also occur upon cell lysis [249]. Removal of contaminants can be achieved by adsorption and/or absorption, but also due to biotransformation outside microalgal cells. Although 88% of climbazole was reported to be removed in *Scenedesmus obliquus* culture, in fact, 99% of climbazole was biotransformed into a byproduct that remained in the culture medium [204].

### 7.4. Limitations for PHRs/PCPs Removal: Microalgal Growth Inhibition

Microalgal-based bioremediation approaches can be limited due to the toxicity of PHRs and PCPs that suppresses the growth of microalgae and can hinder the generation of microalgal biomass used for PHRs/PCPs removal. Indeed, pharmaceutical wastewater effluents were reported to be inhibitory to bacteria and microalgae [250]. The solution can be the cultivation of microalgae in the presence of lower concentrations of PHRs/PCPs.

Some literature data shows that the removal of smaller concentrations of pharmaceuticals can be achieved without decreasing microalgal biomass growth (Table 14). The complete [251] or nearly complete [252] removal of amoxicillin using *Chlorella pyrenoidosa* [252] or *Chlorella* sp. [251] cultures was associated with a partial increase in microalgal cell densities [251] or no effect on microalgal growth [252]. Further, it was reported that the removal of cefradine in *Chlamydomonas reinhardtii* culture was achieved without negative effect on *Chlamydomonas* culture growth rate [253]. Further, a complete removal of florfenicol in *Chlorella* culture without any significant growth inhibition was achieved, although results were dependent on the *Chlorella* strain and antibiotic concentration tested [52]. For estrogens, the nearly complete removal of ethinylestradiol (EE2) by *Chlorella* [215] and a high removal of estradiol (E2) by *Raphidocelis subcapitata* [182] were achieved without negative effect on microalgal growth. Further, triclosan could be completely removed by *Scenedesmus obliquus* without an inhibitory effect on the growth of this microalga [199]. Finally, a complete removal of 7-aminocephalosporanic acid (7-ACA) by *Chlorella* sp., *Mychonastes* sp. or *Chlamydomonas* sp. cultures without any significant negative effect on microalgal growth was reported, and in this case, the mode of 7-ACA removal was mainly via adsorption [254]. These reports show that it is possible to combine PHRs/PCPs removal with microalgal biomass production in one step, if PHRs/PCPs concentrations are maintained below the inhibitory threshold.

Some reports suggest that PHRs can serve as a source of organic carbon for microalgal metabolism to support cell growth. For instance, the removal of paracetamol and salicylic acid by the culture of *Chlorella sorokiniana* was coupled with a significant increase in *Chlorella* biomass productivities, suggesting that these PHRs could be used by microalgae as an additional source of carbon [207]. In another report, a major removal of diclofenac was achieved in *Chlorella sorokiniana*, *Chlorella vulgaris* or *Scenedesmus obliquus* cultures simultaneously to a substantial growth improvement in *Chlorella* biomass production. It was concluded that diclofenac could be also used as a carbon source by microalgae [208]. The use of PHRs as an organic carbon source can provide evidence for the efficient removal of PHRs as contaminants with the additional improvement in microalgal biomass production.

### 7.5. Limitations for PHRs/PCPs Removal: Scaling-Up of Bioremediation Processes

Literature reports referring to the use of microalgae for PHRs/PCPs bioremediation usually present results on a laboratory scale, with small operating volumes processed in flasks or small-scale photobioreactors where media composition is strictly defined, sterility is maintained, and cultivation parameters (light intensity, temperature, pH, agitation, gas transfer) are controlled in real time. However, industrial applications of microalgae-based PHRs/PCPs bioremediation processes will require higher volumes of PHRs/PCPs-containing wastewaters for biotreatment. High wastewater volumes can be processed in large-scale photobioreactors and open systems. Although closed-system photobioreactors are evaluated as a useful tool for the bioremediation of industrial wastewaters [255], open systems (open ponds) are nowadays considered as a realistic scaled-up system due to the simplicity of usage, larger operating volumes and lower processing costs [256]. However, open systems also face several challenges in terms of efficient PHRs/PCPs biotreatments. Microalgal cultivation in open systems is subject to changes in temperature and light intensity that can affect the PHRs/PCPs bioremediation process. For instance, the change in light intensity was reported to greatly affect the efficiency of cefradine and amoxicillin removal in *Microcystis aeruginosa* or *Chlorella pyrenoidosa* cultures [224]. Moreover, the presence of light can cause photodegradation (photo-oxidation, photolysis) of the structure of PHRs and PCPs, depending on light availability and the presence of inorganic/organic molecules [257,258,259]. Such photodegradation products can be more toxic than a parent compound itself [131,188,260]. Large-volume microalgal cultures can require high amounts of nitrogen and phosphorous in wastewaters as sources of nutrients [261,262,263,264] for growth. The concentrations of N and P should be monitored during PHRs/PCPs’ biotreatment because different N and P concentrations can influence microalgal cell–PHRs/PCPs interactions [129,228,240,241]. The change in salinity (↑ NaCl%) could also greatly affect levofloxacin removal in *Scenedesmus obliquus* [101] and *Chlorella vulgaris* [100] cultures. Microalgal open-system cultures are also prone to contamination by wild (micro)organisms, the presence of which can be beneficial [265] or adverse to the microalgae-based PHRs/PCPs bioremediation process. If wastewaters containing numerous PHRs/PCPs can be only partially cleaned due to the inability of a specific microalgal strain to bioremediate some of the PHRs/PCPs, a more advanced strategy is necessary. In such a case, the cocultivation of different strains [265] in one bioremediation system or application of different pre/post-treatment techniques (Section 1) integrated [256] with the microalgal cultivation system should be applied for the successful cleaning of wastewaters. Finally, further disposal of microalgal biomass generated during large-scale PHRs/PCPs bioremediation processes should be considered.

## 8. Applications of Post-Remediation Microalgal Biomass

Microalgae show potential to be used for the bioremediation of industrial and municipal wastewaters contaminated by pharmaceuticals and personal care products (PCPs). Moreover, microalgae contain lipids, pigments and proteins that can become valuable bioproducts in many branches of industry. The ‘whole-cell’ biomass can be used in the form of tablets, pills and powder for human nutrition and animal feed [266]. Bioproducts extracted from microalgal biomass can be further used as nutraceuticals/pharmaceuticals (pigments, fatty acids) or for biofuel production (lipids, fatty acids). Regarding whether microalgal biomass generated during bioremediation process could potentially serve as a source of valuable bioproducts, the negative effect of PHRs and PCPs on microalgal cells should be evaluated. There is no strict literature data evaluating the application of microalgal biomass generated during cultivation in PHRs/PCPs-containing wastewaters. Hence, in this review, the applicational values of biomass generated during PHRs/PCPs bioremediation are evaluated, relying on the literature data concerning the effect of PHRs/PCPs on microalgal metabolism.

### 8.1. Accumulation and Biotransformation of PHRs and PCPs in Microalgal Biomass

Microalgal biomass used in wastewater treatment can contain PHRs/PCPs and their photoproducts adsorbed on the surface of microalgal cells. Microalgal strains can also accumulate PHRs or PCPs inside their cells. For instance, PHRs (tamoxifen, 5-fluorouracil, naproxen, sulfamethazine, clomipramine) or PCP (climbazole and triclosan) can be (bio)accumulated in microalgal cells [85,201,202,204,235,247,267]. Upon cell uptake, PHRs and PCPs can be further (bio)degraded or partially (bio)transformed into byproducts. For instance, naproxen was reported to undergo demethylation, hydroxylation or transformation into conjugate products in microalgal cells [132]. Triclosan underwent degradation via the cleavage of the ether bond, with subsequent transformation into a series of conjugate metabolites, in the diatom *Navicula* sp. [201]. The mode of action of levofloxacin removal was suggested to be bioaccumulation in microalgal cells and biodegradation, involving metabolic steps such as decarboxylation, side-chain cleavage and demethylation, dehydroxylation, ring cleavage and aromatization [100,101]. These generated metabolites might possess unidentified structures and unknown biological activity towards animals and humans. Moreover, bioaccumulated pollutants can affect the content/composition of lipids and pigments in microalgal cells.

### 8.2. Effect of PHRs and PCPs on the Content/Composition of Lipids in Post-Bioremediation Microalgal Biomass

Microalgae are a source of lipids and fatty acids (FAs) that can be used for the production of biodiesel and as food additives. Microalgal biomass generated from cultivation in PHRs/PCPs-containing wastewaters could be used as a source of lipids and fatty acids, although the effect of PHRs/PCPs present in wastewaters on the composition of lipids and FAs in microalgal cells should be considered. PHRs/PCPs effect on microalgal metabolism depends on the specific PHR/PCP tested, which may possess lower or higher toxicity. Moreover, it is crucial whether the removal of the PHRs/PCPs is only due to adsorption on the cell wall or also due to subsequent absorption inside the cell. It is widely reported (see Section 5.2) that the exposure of microalgal cells to PHRs/PCPs causes the degradation of lipids and production of malondialdehyde (MDA). Triclosan (TCS) was also suggested to inhibit fatty acid synthesis and caused irreversible alterations in fatty acids/lipids in *Chlorococcum* sp. [236,240]. On the contrary, cycloheximide (glutarimide antibiotic) increased lipid levels in *Phaeodactylum tricornutum* [268]. Ethynylestradiol (EE2) was also reported to increase lipid content in diatoms. *Navicula incerta* cells affected by ethynylestradiol were reported to contain increased lipid content [180]. In another study, the exposure of *Phaeodactylum tricornutum* to EE2 induced the accumulation of triacylglycerols (TAGs), although this was accompanied by the impairment of eicosapentaenoic acid (EPA, 20:5) synthesis and destabilization of galactolipid vs. phospholipid balance in *Phaeodactylum* cells [269]. In case of green microalgae, exposure of *Dunaliella salina* to ethinylestradiol (EE2) resulted in a decrease in polyunsaturated fatty acids (PUFAs) and increase in saturated fatty acids (SFA) in the total fatty acid composition of *Dunaliella* cells [179]. Literature data shows that PHRs and/or PCPs can alter lipid content and fatty acid composition (saturated/unsaturated, number of C atoms in FA molecule) in microalgal biomass. Besides possessing ‘direct’ effects on lipids, PHRs and PCPs (and/or their metabolites) can be accumulated within the intracellular matrix. Consequently, lipid fractions extracted from biomass with the use of organic solvents can also contain PHRs and PCPs (and/or their metabolites) as contaminants.

### 8.3. Effect of PHRs and PCPs on the Content/Composition of Pigments in Post-Bioremediation Microalgal Biomass

Pigments (carotenoids, chlorophylls) from microalgae can find industrial applications as antioxidants and natural colorants, as well as anti-inflammatory and wound-healing agents. Although stimulation in pigment production in microalgal cells exposed to certain pharmaceuticals was mentioned in some reports (see Section 5.4), many other literature results show decreases in pigment content. For instance, upon being treated with different antibiotics, the decrease in Ch *a* content in *Nostoc* [44], decrease in Chl *a* and *b* in *Selenastrum capricornutum* biomass [80] and alterations (decrease/increase) in the xanthophyll cycle components in *Pseudokirchneriella subcapitata* [222] were reported. Main factors include the microalgal strains tested, but also the pharmaceuticals’ specificity and concentration. Moreover, the problem of PHRs and PCPs being accumulated in biomass and released together with pigments in a form of extracts should be considered.

### 8.4. Application of Post-Bioremediation Living Microalgal Biomass

Microalgal cultures grown in pharmaceutical-contaminated water can be also used as an inoculum with increased resistance for the next bioremediation process. It was reported that microalgae can obtain resistance to contaminants, such as antibiotics, as a result of rare spontaneous mutations [114] and physiological acclimatisation [100]. Indeed, it was reported that pre-exposure of *Microcystis aeruginosa* and *Selenastrum capricornutum* to tetracycline increased their resistance towards this antibiotic [57]. Acclimation of *Chlorella* strain with levofloxacin resulted in the improvement of levofloxacin removal [100]. Isolation of a cyanobacterial strain of *Anacystis nidulans* with resistance to the pyrimidine analogue 5-fluorouracil was reported [270]. Application of PHR-resistant microalgae could possibly improve the rate of pharmaceutical-contained wastewater biotreatment.

## 9. Discussion

In this review, the effect of pharmaceuticals (PHRs) and personal care products (PCPs) on microalgal growth and metabolism and the microalgae-based bioremediation of PHRs and PCPs-containing wastewaters is discussed. Moreover, the possible industrial applications of post-remediation microalgal biomass are evaluated. PHRs and PCPs present in wastewaters can affect the growth of microalgae.

It was shown in the previous section that PHRs and PCPs can effectively inhibit the growth of microalgae and cyanobacteria at concentrations from μg/L to g/L, and these inhibitory concentrations can greatly depend on the strains and toxicants tested. Some strains of green microalgae such as *Chlorella*, *Scenedesmus/Desmodesmus* and *Dunaliella* are commonly considered as a source of biomass for lipid, pigment and protein production [20]. Literature data gathered show that specific PHRs, such as sulfamethoxazole, trimethoprim, clarithromycin, fluoxetine, propranolol, estradiol and ethinylestradiol, as well as PCPs such as triclosan, can inhibit growth of these strains at μg/L concentrations and upward (Table 15). Hence, concentrations of these PHRs/PCPs should be monitored during microalgal growth on PHRs/PCPs-containing wastewaters. Many other green microalgal (mainly *Pseudokirchneriella subcapitata*), diatom and cyanobacterial strains were also reported to be inhibited by numerous PHPs and PCPs (Table 11). However, all literature data gathered for the effect of PHPs and PCPs towards different microalgal strains face in many cases difficulty in interpretation and evaluation regarding which microalgal group is more/less sensitive/resistant or which PHPs and/or PCPs are more or less inhibitory/toxic. Even studies related to the same strain and the same toxicant can vastly vary depending on a particular literature report. Some explanations for such differences in inhibitory/toxic concentrations can be provided. Growth inhibitory/toxicity tests can be carried out in Erlenmeyer flasks, multiwell (micro)plates [39] and test tubes [74], and also containing algal biofilms [271]. Growth inhibition rates in microalgal cultures are calculated based on measuring cell number, biomass concentration, optical density (OD_520_, OD_730_, OD_680_), oxygen evolution, fluorescence and chlorophyll concentration. Using different measurement methods can result in differences in conclusions regarding inhibitory ranges of tested toxicants/inhibitors. For example, chlorophyll content in cells exposed to toxicants can change during cultivation, thereby providing different results to cell number counting. Moreover, the difference between growth inhibitory concentration and photosynthesis inhibitory concentration can be as much as 7-fold, with the growth being more sensitive to the toxicant than the photosynthesis [203]. Moreover, the inhibitory/toxic effect can be exposure time-dependent, as exposure times from 1 h to 15 days are used in different literature reports (Table S2). Toxic/inhibitory activity of PHRs can also depend on pH [152], and pH value can change during microalgal cultivation [56]. pH-dependent toxicity of PHRs should be taken into consideration, as different microalgal strains can be grown within a pH range of 2 [272] to 11 [273]. Toxicity of PHRs towards microalgae can also depend on the enantiomeric form of the pharmaceutical tested [175].

Exposure of microalgal cells to PHRs and/or PCPs affects intracellular structure (proteins, pigments, lipids, carbohydrates) and machinery (enzymes) and causes the release of substances (toxins, extracellular matrix) from cells.

Pharmaceuticals were reported to cause chlorophyll biosynthesis inhibition [80] and photosynthesis inhibition [230] in microalgal cells/cultures, and the chloroplast was concluded to be the main target of pharmaceuticals [226]. Moreover, antibiotics modulated (increased or decreased) the xanthophyll cycle pool size and xanthophyll cycle conversion state in *Pseudokirchneriella subcapitata* [222]. Streptomycin interfered with the synthesis of proteins involved in the photosynthetic mechanism in *Chlorella vulgaris* cells [73]. Antibiotics were reported to completely inhibit protein synthesis in cyanobacteria [274], blocked the induction of the expression of desaturases (*des*) in *Synechocystis* [275] and the synthesis of phycoerythrin in *Fremyella diplosiphon* [276] and phycocyanin in *Anacystis nidulans* [232]. Treatment of *Euglena gracilis* with antibiotic netilmicin caused inhibition of chlorophyll synthesis, alteration in plastid structure and bleaching of green *Euglena* cells [277].

Accumulation of PHRs and/or PCPs inside cells results in oxidative stress with the generation of reactive oxygen species (ROS) that can have effects on synthesis of pigments and cause the degradation of polyunsaturated fatty acids (PUFAs). Oxidative stress metabolites are biomarkers of microalgal cells exposed to numerous eco-toxicants [278]. PHRs and PCPs-induced oxidative stress exerts an impact on pigments such as chlorophylls and carotenoids. It was previously reported that the exposure to metals caused a decrease in chlorophyll content [279,280,281], with the formation of chlorophyll derivatives [281] and increase in carotenoid content [279,282,283] in microalgal biomass, although the stimulation of chlorophyll synthesis at low metal concentrations was also reported [283]. Literature data gathered regarding the influence of PHRs and PCPs show the decreasing/increasing effects on both chlorophylls and carotenoids. Lipids are another target for oxidative stress. Exposure to metals resulted in lipid peroxidation and the increase in malondialdehyde (MDA), a product of lipid degradation, inside microalgal cells [1,283,284]. Oxidative stress caused by PHRs or PCPs also resulted in the generation of MDA, with its increase by as much as an order of magnitude in microalgae (Table 12). As a result of exposure to PHRs, the activity of antioxidant enzymes (SOD, CAT, POD) in microalgal cells is increased or decreased (Table 13). Exposure to PHRs can cause the accumulation of proline [97,223] and many other free amino acids [223] in microalgal cells.

Although PHRs and PCPs can inhibit microalgal growth and adversely affect microalgal metabolism, living microalgae can be harnessed for the removal and biodegradation of PHRs or PCPs present in wastewaters. Microalgal cultures are considered as a “green” technology for the remediation of wastewaters contaminated by PHRs and PCPs. Removal of PHRs and PCPs from wastewaters can be achieved via adsorption on the surface of microalgal cell biomass and absorption (accumulation) inside microalgal cells, as well as via intracellular and/or extracellular biotransformation/biodegradation [18]. Efficiency of pollutant removal is dependent on microalgal strain adaptability and specificity towards a target pollutant, as well as initial microalgal cell densities, initial pollutant loadings, enantiomeric form of the pollutant [261], contact time and cultivation parameters (pH, salinity, light intensity). Removal of amoxicillin and cefradine by *Microcystis aeruginosa* was also greatly improved in the presence of NaAc or glucose [285].

It was stated that successful microalgae-based bioremediation processes should not involve the generation of toxic transformation products [286]. However, microalgae are cultivated in the presence of light that can cause photodegradation [257,258,259] and generation of toxic byproducts [131,188,260]. Besides the generation of photoproducts, some of the human metabolites of the parent (original) pharmaceutics can also be more toxic to microalgae [166]. Furthermore, pharmaceuticals at ng/L concentrations can already modulate synthesis and release of toxins (such as microcystin) in *Microcystis* culture. The presence of hepatotoxic and neurotoxic secondary metabolites [287] released from cyanobacteria (such as *Microcystis*) during wastewater bioremediation should be taken into consideration and monitored.

Wastewaters can contain different pharmaceuticals and other organic pollutants that in the mixture can cause synergistic and/or additive effects on the growth inhibition of microalgae [55,75,104,109,153]. Synthetic and natural organic molecules, as well as inorganic (metals, NPs) compounds, can affect microalgae–PHRs/PCPs interactions (Section 6) and alter PHRs/PCPs bioremediation by microalgal cultures.

Microalgal cells can find applications for PHRs and PCPs-containing wastewater bioremediation. Microalgal biomass can also find applications as a source of lipids, proteins and pigments for different branches of industry. Merging these two approaches could lead to the development of a new type of advanced sustainable processes where valuable microalgal biomass is generated and PHRs and PCPs-containing wastewaters are cleaned in a single step. However, three limitations for the successful development of such a process can appear. Firstly, the inhibitory effect of PHRs and PCPs present in wastewaters can reduce microalgal growth and biomass production and can also decrease the removal rate of PHRs and PCPs; secondly, low efficiency of PHRs and PCPs removal in microalgal cultures can occur; thirdly, potential problems can arise regarding applications of microalgal biomass containing adsorbed/absorbed pollutants.

Although microalgal growth is limited by PHRs and PCPs at concentrations of μg/L–mg/L and PHRs and PCPs in wastewaters (Table S1) are present at much smaller concentrations (ng/L–μg/L), some reports suggest that PHRs can negatively affect microalgal growth at μg/L and even ng/L concentrations. Moreover, wastewaters can contain mixtures of different PHRs and/or PCPs that can act simultaneously to trigger/increase inhibitory effects on microalgal cultures. The obvious solution would be maintaining PHRs and/or PCPs concentrations in wastewaters below inhibitory thresholds. Another possibility could be the selection of strains (screening) [288,289] with higher resistance towards specific pollutants present in wastewaters subjected to a microalgae-based bioremediation process. Moreover, it should be noted that the inhibitory effect of pharmaceuticals can greatly differ between PHRs and microalgae/cyanobacteria tested. Some PHRs possess high toxicity, but others are weak toxicants. Low toxicity may be due to the fact that some PHRs molecules do not enter the microalgal cell interior, instead staying outside cells and hence not causing negative effect on microalgal metabolism and growth. It was reported that different PHRs are accumulated to very different extents (from 0% to 100%) by different microalgae [247]. It was also concluded that lipophilic compounds are accumulated by microalgae more efficiently than hydrophilic ones [247]. The toxicity of PHRs to microalgal cells can be also greatly affected by pH level, which determines the charge of the PHR molecule and its ‘accumulation potential’, as well as by the presence of inorganic/organic matter.

Although the majority of literature data show the inhibitory effect of PHRs on microalgal growth and metabolism, some reports suggest that stimulatory effect of PHRs on microalgal growth is also possible. Some PHRs at ng/L (ethinylestradiol, ciprofloxacin, sulphamethoxazole, amoxicillin), μg/L (ethinylestradiol, tamoxifen, bezafibrate, tilmicosin) or mg/L (florfenicol, NSAIDs) concentrations were reported to stimulate microalgal growth (Section 4). When compared with data for other organic toxicants used in algal tests, the stimulatory effect of petroleum hydrocarbons at lower concentrations on diatom growth was attributed to the ability of microalgae to use contaminants as a carbon source [290]. Moreover, the stimulation of metabolism in microalgal cells at smaller concentrations of toxicants was reported to be possible due to hormesis [291].

Besides avoiding growth inhibition, the highest rate of PHRs and/or PCPs removal should be achievable. High (50–100%) removal rates were reported to be achieved in many experimental works (see Section 7). However, low removal rates were also mentioned. Low removal rates could be greatly improved by modifying cultivation media, for example, increasing salinity [100,101] or adding exogenous organic carbon [285], although alterations in cultivation media can negatively affect microalgal growth [100,101].

Microbial contamination can pose a problem for microalgal cultivation [292], and pharmaceuticals can be also used to eliminate bacterial [293,294,295,296] and fungal contamination [293,295] from microalgal cultures.

Microalgal strains can bioremediate contaminants such as PHRs and PCPs during cultivation in different systems ranging from flasks to scaled-up closed-system photobioreactors and open ponds. For future advancements, microalgal cultures could be maintained in microfluidic photobioreactors (droplet, digital, flow-based) [297,298] for possible PHRs and PCPs removal and for studying interactions between a single cell and PHRs/PCPs molecules at the microscale. Alternatively, microalgal cells could be integrated with a microfluidic chip to form a sensor for real-time monitoring [299] of the PHRs/PCPs contamination in water streams/bodies.

Besides proper cultivation systems, microalgae require microelements and macroelements (nitrates, ammonium, phosphates) [261,262,263], which can be found at different concentrations in wastewaters.

Microalgal biomass generated during cultivation in wastewaters could find applications in industry. However, PHRs and PCPs removed from wastewaters by microalgae might have negative effects on industrial applications of post-remediation microalgal biomass. PHRs/PCPs can modify cellular composition (lipids, pigments, proteins, carbohydrates) in microalgal cells (see Section 5), thereby altering and deteriorating the nutritional value of microalgal biomass as food/feed supplements. Microalgal biomass, upon pharmaceutical wastewater treatment, can become ‘degenerated’ due to toxic compounds attached to the cell surface or accumulated inside cells and composed of partially decomposed cellular macromolecules. Lipid and pigment extracts obtained from contaminated microalgal biomass can also contain these contaminants that are ‘transferred’ outside from the cell surface and interior during organic solvent extraction. Fractions extracted from microalgal biomass could be purified by means of chromatographic/sorption–desorption methods to remove PHRs and PCPs, as undesirable contaminants, and to obtain a clean extract as a potential product.

Microalgal biomass containing absorbed/adsorbed toxic PHRs and PCPs should be stored in sealed containments to prevent the release of toxic substances from decomposing biomass into the environment. Microalgal biomass can be used as a feedstock for microorganisms (yeast, bacteria) producing biofuels (ethanol, bioethanol, biodiesel, biogas), but the presence of PHRs and PCPs can have a detrimental effect on yeast and bacteria. A promising alternative could be the thermal treatment of microalgal PHRs/PCPs-containing biomass via pyrolysis techology. Pyrolysis is a process where biomass is treated at high temperature and in the absence of oxygen to produce py-oil, py-gas and biochar, with a broad spectrum of industrial applications [300,301]. Production of bio-oil, gases and biochar from microalgal biomass pyrolysed at 300–750 °C has been reported [302,303]. Moreover, pyrolysis enabled the volatilisation and degradation of PHRs (antibiotics, antihistamine and antiepileptic agents) [304] and PCPs (triclosan, triclocarban) [300] already at 200–300 °C [300,304]. Potentially, the pyrolysis process could convert PHRs and PCPs present in microalgal biomass into volatile degradation products, with simultaneous transformation of microalgal biomass into biochar and bio-oil.

## 10. Conclusions

This review discusses the potentials and limitations of combining microalgae-based bioremediation of PHRs and PCPs-containing wastewaters with the production of microalgal biomass for high value-added compounds. Integrating the use of microalgal cultures to remove PHRs and PCPs from wastewaters with microalgal cultivation for biomass generation can create an attractive green technology process where the utilisation of problematic wastewaters and production of microalgal cells containing biomolecules of market value can be achieved in one step.

However, this interesting approach faces a series of possible limitations to overcome. Sensitivity and resistance of different eukaryotic microalgal and cyanobacterial species strictly depends on strains and PHRs and PCPs molecules used. Therefore, selection of a strain for cultivation has to be done in accordance with the composition of PHRs/PCPs wastewaters. Additionally, multiple effects of different components in wastewaters on microalgal growth should be taken into consideration. Inhibitory concentrations of PHRs or PCPs should be avoided to achieve unperturbed microalgal growth and maximise microalgal biomass production. Concentrations of PHRs and PCPs in wastewaters should also be set at such concentrations to achieve the removal of the highest amount of pollutants without negative effect on microalgal growth. Mechanisms of PHRs or PCPs removal, including extracellular biodegradation, adsorption on the cell surface, absorption in the cell and/or intracellular biodegradation, depend on the species used and the pollutant molecule structure. The process of pollutant removal can also change with the alteration of cultivation parameters (pH, light intensity) and media composition (inorganic/organic matter). Exposure of microalgae to PHRs/PCPs can cause the stress in microalgal cells, thereby altering cellular composition, potentially including the degradation of cellular compounds that were initially planned to be produced for industry. The most convienient mode seems to be if PHRs/PCPs only adsorb on the surface of living microalgae without being ‘transferred’ inside cells, thereby avoiding detrimental effects on metabolism and growth. However, many PHRs and PCPs are absorbed/accumulated by different microalgal strains.

The solution to surpass existing limitations could be the development of microalgal strains that are capable of simultaneously tolerating the presence of different PHRs and PCPs and delivering high growth rate, efficiently removing PHRs and PCPs from wastewater media, detoxifying (biodegrading) absorbed toxicants into harmless byproducts and containing high-quality (nondegraded) target industrial bioproducts that are to be obtained from the microalgal biomass. Such an ‘ideal’ cellular model has not been developed so far and remains a challenge for future research.

## Figures and Tables

**Table 1:** Summary of the 50% growth inhibitory/toxicity ranges of florfenicol (FF) towards different microalgae.

Microalgae	Concentrations of FF (mg/L) Causing 50% Growth Inhibition/Toxicity	Ref.
*Pseudokirchneriella subcapitata*	2.9	[48]
*Tetraselmis*	1.3–11	[41,42,49]
*Isochrysis galbana*	8	[41]
*Scenedesmus vacuolatus*	18	[50]
*Chlorella*	14–215	[41,51,52]
*Skeletonema costatum*	5	[53]
*Microcystis flosaquae*	0.05	[46]

**Table 2:** Summary of the 50% inhibitory/toxicity ranges of sulfamethoxazole (SMX) towards different microalgae.

Microalgae	Concentrations of SMX (mg/L) Causing 50% Growth Inhibition/Toxicity	Ref.
*Scenedesmus*	0.12–1.54	[76,77]
*Chlorella vulgaris*	0.98–1.57	[78,79]
*Selenastrum capricornutum*/ *Pseudokirchneriella subcapitata*	0.146–2.5 ^(25–50% GI)^	[45,65,80,81,82]
*Pseudokirchneriella subcapitata*	>9 ^PI^	[66]
*Desmodesmus subspicatus*	0.25–210 ^PI^	[83,84]
*Microcystis aeruginosa*	0.55 ^PI^	[66]
*Synechococcus leopolensis*	0.027	[82]
*Cyclotella meneghiniana*	2.4	[82]

GI: growth inhibition; PI: photosynthesis inhibition.

**Table 3:** Summary of the 50% inhibitory/toxicity ranges of lincomycin (LIN) towards different microalgae.

Microalgae	Concentrations of LIN (mg/L) Causing 50% Growth Inhibition/Toxicity	Ref.
*Pseudokirchneriella subcapitata*	0.07–3.26	[65,91,120]
*Desmodesmus subspicatus*	7.08	[91]
*Cyclotella meneghiniana*	1.6	[120]
*Cylindrotheca closterium*	14	[109]
*Navicula ramosissima*	11	[109]
*Anabaena flosaquae*	0.057	[91]
*Synechococcus leopoliensis*	0.042–0.195	[91]

**Table 4:** Summary of the 50% inhibitory/toxicity ranges of fluoxetine towards different microalgae.

Microalgae	Concentrations of Fluoxetine (mg/L) Causing 50% Growth Inhibition/Toxicity	Ref.
*Pseudokirchneriella subcapitata/Raphidocelis subcapitata*	0.024–0.2	[115,145,146,147,148,149,150]
*Chlorella*	0.036–4.3	[112,150,151]
*Scenedesmus*	0.024–0.207	[150,151,152]
*Acutodesmus obliquus*	5	[129]
*Chlamydomonas*	0.25–1.6	[129,151]
*Ankestrodesmus falcatus*	0.04	[112]
*Dunaliella*	0.05–0.169	[139,151]
*Skeletonema*	0.018–0.043	[147,148,153]

**Table 5:** Summary of the 50% inhibitory/toxicity ranges of clofibric acid towards different microalgae.

Microalgae	Concentrations of Clofibric Acid (mg/L) Causing 50% Growth Inhibition/Toxicity	Ref.
*Pseudokirchneriella subcapitata*	75–94	[82,137]
*Scenedesmus/Desmodesmus subspicatus*	89–115	[143,160]
*Tetraselmis chuii*	318	[159]
*Dunaliella tertiolecta*	224	[139]
*Chlorella pyrenoidosa*	100–150	[107]
*Anabaena sp.*	30–48	[158]
*Synechococcus leopolensis*	40.2	[82]

**Table 6:** Summary of the 50% inhibitory/toxicity ranges of carbamazepine (CBZ) towards different microalgae.

Microalgae	Concentrations of CBZ (mg/L) Causing 50% Growth Inhibition/Toxicity	Ref.
*Micractinium reisseri*	100	[167]
*Scenedesmus/Desmodesmus*	54–201	[160,167,168]
*Chlorella*	33–1339	[168,169]
*Dunaliella tertiolecta*	53–296	[170]
*Cyclotella meneghiniana*	31.6	[82]
*Phaeodactylum tricornutum*	62	[90]
*Synechococcus leopolensis*	33.6	[82]

**Table 7:** Summary of the 50% inhibitory/toxicity ranges of propranolol (PRO) towards different microalgae.

Microalgae	Concentrations of PRO (mg/L) Causing 50% Growth Inhibition/Toxicity	Ref.
*Pseudokirchneriella subcapitata*	0.77–7.4	[82,115,172]
*Scenedesmus/Desmodesmus*	0.118–24	[84,152,160,173]
*Acutodesmus obliquus*	19	[129]
*Chlamydomonas reinhardtii*	3	[129]
*Chlorella vulgaris*	0.259 ^(F = 21%)^	[174]
*Skeletonema pseudocostatum*	0.236	[153]
*Cyclotella meneghiniana*	0.24	[82]
*Phaeodactylum tricornutum*	0.29	[90]
*Synechococcus leopolensis*	0.67	[82]

F: growth inhibition factor.

**Table 8:** Summary of the 50% inhibitory/toxicity ranges of ethynylestradiol (EE2) towards different microalgae.

Microalgae	Concentrations of EE2 (mg/L) Causing 50% Growth Inhibition/Toxicity	Ref.
*Pseudokirchneriella subcapitata/Raphidocelis subcapitata*	0.01–0.8	[103,176]
*Scenedesmus/Desmodesmus*	0.04–144	[84,103,176,177,178]
*Chlorella vulgaris*	70–121	[178]
*Dunaliella salina*	0.001	[179]
*Navicula incerta*	3.2	[180]
*Microcystis aeruginosa*	1.48	[103]
*Anabaena variabilis*	71–100	[181]

**Table 9:** Summary of the 50% inhibitory/toxicity ranges of estradiol (E2) towards different microalgae.

Microalgae	Concentrations of E2 (mg/L) Causing 50% Growth Inhibition/Toxicity	Ref.
*Pseudokirchneriella subcapitata/Raphidocelis subcapitata*	0.01–0.87	[176]
*Desmodesmus subspicatus*	0.08–1.07	[176]
*Scenedesmus armatus*	206–522	[178]
*Chlorella vulgaris*	105–242	[178]
*Anabaena variabilis*	438–2002	[181]

**Table 10:** Summary of the 50% inhibitory/toxicity ranges of triclosan (TCS) towards different microalgae.

Microalgae	Concentrations of TCS (mg/L) Causing 50% Growth Inhibition/Toxicity	Ref.
*Pseudokirchneriella subcapitata/Raphidocelis subcapitata/Selenastrum capricornutum*	0.00053–0.037	[81,157,190,191,192,193]
*Chlamydomonas*	0.4^(36% inhibition)^–4	[151,194,195]
*Dunaliella*	0.0035–0.16	[139,151]
*Tetraselmis suecica*	0.8 ^(80% inhibition)^	[196]
*Scenedesmus/Desmodesmus*	0.0014–0.4	[151,197,198,199]
*Chlorella*	0.4–1.44	[151,199,200]
*Skeletonema pseudocostatum*	0.027	[153]
*Navicula* sp.	0.145–0.173	[201]
*Microcystis aeruginosa*	0.0092	[203]
*Anabaena flosaquae*	0.0016	[197]

**Table 11:** General inhibitory/toxicity ranges of pharmaceuticals (PHRs) and personal care products (PCPs) towards different microalgal groups.

General Inhibitory/Toxicity Ranges of PHPs and PCPs towards Different Microalgal Groups *							
PHPs or PCPs	Green Microalgae (All Strains)	Green Microalgae (Pseudokirchneriella subcapitata **)	Green Microalgae (*Chlorella* strains)	Diatoms (All Strains)	Other Microalgae	Cyanobacteria (All Strains)	Cyanobacteria (*Microcystis* Strains)
Phenicols	0.47–1283 mg/L	2.7–8.9 mg/L	14–1283 mg/L	5–12 mg/L	8–158 mg/L	0.05 to ≥25 mg/L	0.05–0.43 mg/L
Tetracyclines	0.17–40 mg/L	0.17–4.5 mg/L	7–37.8 mg/L	n.d.	1.6 mg/L	≥0.0015–100 mg/L	≥0.05–15.2 mg/L (20 mg/L)
Aminoglycosides	0.133–30 mg/L (66 mg/L ^MIC^)	0.133–19.2 mg/L	2.4–66 mg/L ^MIC^	6.6 mg/L ^MIC^	n.d.	0.007–100 mg/L	0.007–1.6 mg/L
Sulphonamides	0.146–210 mg/L	0.146 to >9 mg/L	0.98–17.74 mg/L	0.11–2.4 mg/L	1.44–403 mg/L	0.027 to >2000 mg/L	0.135–500 mg/L
Trimethoprim	0.0078–130 mg/L	>9–130 mg/L	90–123 mg/L	2.13–21.6 mg/L	16 mg/L	0.003–253 mg/L (>200 mg/L)	6.9 to >200 mg/L
β-lactams	≥12 mg/L	≥400 mg/L	>1000 mg/L	n.d.	3100 mg/L	0.0002 to >200 mg/L	0.0002 to ≥0.2 mg/L (≥3 mg/L)
Quinolones	1.44–150 mg/L (>120 mg/L)	1.44–>120 mg/L	12.2–150 mg/L	0.09–72 mg/L	10–24 mg/L	>0.006–5.6 mg/L	>0.006–0.18 mg/L
Macrolides	0.002–13 mg/L	0.002–2.3 mg/L	3–12 mg/L	0.27 to >60 mg/L ^MIC^	1–30 mg/L ^MIC^	0.001–10 mg/L	0.001–0.29 mg/L
Glycopeptides	371–724 mg/L	371–724 mg/L	n.d.	n.d.	n.d.	n.d.	n.d.
Lincosamides	0.01–7 mg/L	0.01–3.26 mg/L	n.d.	1.6–14 mg/L	n.d.	0.042–0.195 mg/L	n.d.
Nitrofurans	0.5– 17 mg/L	n.d.	1.3 mg/L	0.5 mg/L	0.5 mg/L	n.d.	n.d.
Nitroimidazoles	0.0078–705 mg/L	40.4–56.6 mg/L	12.5–38.8 mg/L	n.d.	n.d.	117 mg/L	117 mg/L
Quinoxalines	1.72–40 mg/L	1.72–40 mg/L	n.d.	n.d.	n.d.	5 mg/L	5 mg/L
Ansamycins	171 mg/L	171 mg/L	n.d.	n.d.	n.d.	4–10 mg/L	n.d.
NSAIDs	10–620 mg/L	10–240 mg/L	40–150 mg/L	19.2–255 mg/L	965 mg/L	12.3–14.5 mg/L	n.d.
Acetaminophen	88 to >240 mg/L	>240 mg/L	88 to >240 mg/L	266 mg/L	n.d.	n.d.	n.d.
Antidepressants	0.012–99.8 mg/L	0.012–99.8 mg/L	0.036–10.2 mg/L	0.0019–6.9 mg/L	n.d.	0.200 mg/L	0.200 mg/L
Lipid regulators	15–318 mg/L	15–103 mg/L	100–150 mg/L	n.d.	n.d.	4–48 mg/L	n.d.
Antineoplastic agents	0.075–260 mg/L	0.075 to >100 mg/L	0.61 mg/L	0.014 mg/L	n.d.	0.67–17 mg/L	6 mg/L
Antiepileptic agents	0.1–1339 mg/L	28.3 to >100 mg/L	33–1339 mg/L	31.6–62 mg/L	n.d.	33.6 mg/L	n.d.
Beta-blockers	0.118 to >100 mg/L	0.77–190 mg/L	0.259–25.9 mg/L	0.24–312 mg/L	n.d.	0.67 mg/L	n.d.
Estrogens	0.001–522 mg/L	0.01–0.87 mg/L	70–242 mg/L	3.2 to >10 mg/L	n.d.	1.48–2002 mg/L	1.48 mg/L
Triclosan	0.00053–4 mg/L	0.00053–0.037 mg/L	0.4–1.44 mg/L	0.027–0.173 mg/L	n.d.	0.0016–0.0092 mg/L	0.0092 mg/L

* Data mostly based on concentrations causing 50% growth inhibition or toxicity, although 30–100% inhibitory concentrations or the MIC were also included in some cases; ** *Selenastrum capricornutum* = *Pseudokirchneriella subcapitata* = *Raphidocelis subcapitata.* MIC: minimal inhibitory concentration; n.d.: no data available to the best of the authors’ knowledge.

**Table 12:** Effect of PHPs and PCPs on MDA production in different microalgae.

PHRs/PCPs	Concentration in Medium * (mg/L)	Strain	Maximal MDA Increase **	Ref.
Amoxicillin	20	*Microcystis aeruginosa*	1.18-fold	[219]
Amoxicillin	0.001	*Microcystis aeruginosa*	~2.6-fold	[220]
Amoxicillin	0.0005	*Microcystis aeruginosa*	~1.7-fold	[221]
Amoxicillin	0.0005	*Microcystis aeruginosa*	~1.5-fold	[211]
Ciprofloxacin	100	*Chlamydomonas mexicana*	~4-fold	[108]
Ciprofloxacin	2.5	*Pseudokirchneriella subcapitata*	~2.2-fold	[222]
Enrofloxacin	100	*Chlamydomonas mexicana*	~1.8-fold	[95]
		*Micractinium resseri*	~9.3-fold	
Enrofloxacin	80	*Scenedesmus obliquus*	2.4-fold	[97]
Norfloxacin	20	*Microcystis aeruginosa*	1.48-fold	[219]
Norfloxacin	60	*Scenedesmus obliquus*	1.86-fold	[99]
Levofloxacin	0.1	*Microcystis flosaquae*	3.84-fold	[102]
Tetracycline	0.5	*Microcystis aeruginosa*	1.75-fold	[57]
	5	*Selenastrum capricornutum*	~6-fold	
Doxycycline	5	*Microcystis aeruginosa*	1.8-fold	[71]
Chlortetracycline	60	*Chlorella pyrenoidosa*	2.5-fold	[64]
	48	*Microcystis aeruginosa*	2.7-fold	
Florfenicol	0.025	*Microcystis flosaquae*	2.06-fold	[46]
Florfenicol	46 → 160	*Chlorella* sp.	25.6-fold ^F^	[52]
Thiamphenicol	0.07	*Microcystis flosaquae*	~2.3-fold	[46]
Erythromycin	0.03	*Microcystis flosaquae*	~5-fold	[113]
Erythromycin	0.3	*Pseudokirchneriella subcapitata*	~3.2-fold	[222]
Spiramycin	0.001	*Microcystis aeruginosa*	~3.2-fold	[220]
Sulfamethoxazole	2.5	*Pseudokirchneriella subcapitata*	~1.9-fold	[222]
Carbamazepine	200	*Dunaliella tertiolecta*	~9-fold	[170]
Ethinylestradiol	0.001	*Dunaliella salina*	~2.37-fold	[179]
Triclosan	0.405	*Chlamydomonas reinhardtii*	1.33-fold	[194]
Triclosan	4	*Chlamydomonas reinhardtii*	~6.5-fold	[195]

*: PHRs/PCPs concentration corresponding to the maximal uplift of MDA; **: in comparison to the control (no PHRs/PCPs); ^F^: in comparison to florfenicol at 46 mg/L; →: increase in concentration.

**Table 13:** Effect of PHPs and PCPs on antioxidant enzyme activity in different microalgae.

Strain	PHR/PCP	Concentration in Medium (mg/L)	SOD Activity *	CAT Activity *	POD Activity *	Ref.
*Microcystis flosaquae*	ERY	0.04	3.86-fold ↑	4.8-fold ↑	-	[113]
*Microcystis flosaquae*	FF TAP	0.05 0.1	2.7-fold ↑ 1.7-fold ↑	5.3-fold ↑ 3.4-fold ↑	- -	[46]
*Microcystis aeruginosa*	TET	0.5	~2.9-fold ↑	-	-	[57]
*Microcystis aeruginosa*	CTC	48	3-fold ↑	-	-	[64]
*Microcystis aeruginosa*	DOX	10	No change	3-fold ↑	-	[71]
*Microcystis aeruginosa*	CTC	0.05	~25% ↓	~10% ↑	~90% ↑	[69]
*Microcystis aeruginosa*	SPI	0.001	2.1-fold ↑	75% ↓	71% ↓	[220]
*Microcystis aeruginosa*	NOR	20	36% ↓	-	-	[219]
*Microcystis aeruginosa*	AMO	20	57% ↓	-	-	[219]
*Microcystis aeruginosa*	AMO	0.001	1.6-fold ↑	2.2-fold ↑	1.3-fold ↑	[220]
*Selenastrum capricornutum*	TET	2	1.6-fold ↑	-	-	[57]
*Chlorella pyrenoidosa*	CTC	60	3.5-fold ↑	-	-	[64]
*Chlamydomonas mexicana*	CPX	100	8.5-fold ↑	-	-	[108]
*Pseudokirchneriella subcapitata*	ERY CPX SMX	0.3 2.5 2.5	15% ↓ 1.3-fold ↑ 1.2-fold ↑	6% ↓ 1.1-fold ↑ 1.06-fold ↑	- - -	[222]
*Scenedesmus obliquus*	CBZ	10	1.78-fold ↑	2.68-fold ↑	-	[168]
*Chlorella pyrenoidosa*	CBZ	10	3.5-fold ↑	2.46-fold ↑	-	[168]
*Scenedesmus obliquus*	CBZ	50 200	60% ↓ ~100% ↓	~1.25-fold ↑ ~10-fold ↑	- -	[167]
*Chlamydomonas mexicana*	CBZ	50 200	1.3-fold ↑ 62% ↓	- -	- -	[167]
*Chlorella vulgaris*	Fluoxetine	0.04	54% ↓	46% ↓	-	[112]
*Ankestrodesmus falcatus*	Fluoxetine	0.036	40% ↓	52% ↓	-	[112]
*Navicula incerta*	EE2	4	1.6-fold ↑	-	25% ↓	[180]
*Dunaliella salina*	EE2	0.001	1.4-fold ↑	25% ↓	-	[179]
*Microcystis aeruginosa*	TCS	0.00025 0.002	1.3-fold ↑ 55% ↓	- -	No change No change	[203]

*: The change in comparison to the control (no PHR/PCP); ↑: increase; ↓: decrease; -: no data.

**Table 14:** Removal rate of PHRs/PCPs by microalgal cultures with noninhibitory effect on microalgal growth/biomass production.

Pharmaceutical Type	Pharmaceutical Concentration	Pharmaceutical Removal	Microalgal Strain	Effect on Growth/Biomass Production	Ref.
Amoxicillin (AMX)	10–150 mg/L	99% within 2 h	*Chlorella* sp.	Up to 20% stimulation after 12 h	[251]
Amoxicillin (AMX)	100–500 mg/L	85–97% after 48 h	*Chlorella pyrenoidosa*	No effect on growth	[252]
Cefradine	0.5–10 mg/L	Up to 50% ^v^ within 2 h	*Chlamydomonas reinhardtii*	Slight growth stimulation possible (within 7 days)	[253]
Florfenicol	46 mg/L	97% (20 days)	*Chlorella* sp.	Slight inhibition	[52]
Ethinylestradiol (EE2)	5 mg/L	94% (9 days)	*Chlorella*	No effect on growth (9 days)	[215]
Estradiol (E2)	0.1–0.5 mg/L	~80% (4 days)	*Raphidocelis subcapitata*	No effect on growth (4 days)	[182]
Triclosan	0.4 mg/L	~100% after 1 day	*Scenedesmus obliquus*	No effect on growth after 4 days	[199]
7-Aminocephalosporanic acid (7-ACA)	100 mg/L	99% within 125 h ^H&P^	*Chlorella* sp. *Mychonastes* sp. *Chlamydomonas* sp.	Slight inhibition (up to 12%) after 200 h	[254]
Paracetamol	25 mg/L 250 mg/L	67% (7 days) 48% (7 days)	*Chlorella sorokiniana*	42% increase (8 days) 28% increase (7 days)	[207]
Salicylic acid	25 mg/L 250 mg/L	73% (4 days) 94% (4 days)		28% increase (8 days) No increase (7 days) ^C^	
Diclofenac	25 mg/L	65% (9 days)	*Chlorella sorokiniana*	53% improvement (9 days)	[208]
		69% (9 days)	*Chlorella vulgaris*	43% improvement (9 days)	
		98% (9 days)	*Scenedesmus obliquus*	10% improvement (9 days)	

^V^: depending on various parameters: cell density, pH, temperature; ^H&P^: hydrolysis and photolysis also reported; ^C^: prolongation of growth between day 7 and 12 of cultivation with a 2-fold increase in biomass density.

**Table 15:** Sensitivity of microalgae (*Chlorella*, *Scenedesmus*/*Desmodesmus*, *Dunaliella*) to specific PHRs/PCPs concentrations in wastewaters for monitoring.

Microalgae	Specific PHR/PCP	Orientative Concentration (μg/L) in Wastewaters to Monitor
*Scenedesmus*/*Desmodesmus*	Sulfamethoxazole	≥120
*Desmodesmus*	Trimethoprim	≥7.8
*Desmodesmus*	Clarithromycin	32
*Chlorella* *Scenedesmus* *Dunaliella*	Fluoxetine	≥36 ≥24 ≥50
*Scenedesmus* *Chlorella*	Propranolol	≥118 ≥259
*Desmodesmus*	Estradiol	≥80
*Desmodesmus* *Dunaliella*	Ethinylestradiol	≥40 ≥1
*Scenedesmus* *Dunaliella* *Chlorella*	Triclosan	≥1.4 ≥3.5 ≥400

## Supplementary Materials

Supplementary materials can be found online https://www.mdpi.com/1422-0067/20/10/2492/s1.
