# Supplementary material for: Effect of PHRs and PCPs on Microalgal Growth, Metabolism and Microalgae-Based Bioremediation Processes: A Review
Source: Int J Mol Sci. 2019 May 20;20(10):2492. doi: 10.3390/ijms20102492 (PMC6567089; doi:10.3390/ijms20102492)
Supplement: Supplementary file 1 [file ijms-20-02492-s001.pdf]

Krystian Miazek and Beata Brozek-Pluska

Effect of PHRs and PCPs on Microalgal Growth, Metabolism and Microalgae-Based Bioremediation Processes: a Review

Table S1. Examples for the occurrence of PHRs and PCPs in different types of wastewaters.

Wastewater type	PHRs or PCPs	Class	Exemplary concentrations [μg/L]	Ref.
Hospital wastewaters	Ampicillin	β-lactams	53	[22]
	Vancomycin	Glycopeptides	14	
	Trimethoprim	Trimethoprim	30	
Hospital wastewaters	Tetracyclines	Tetracyclines	0.36-0.75	[23]
	Sulphonamides	Sulphonamides	0.28-0.63	
	Quinolones	Quinolones	1.35-1.92	
Hospital wastewaters	Ciprofloxacin	Quinolones	0.01-100	[24]
	Tamoxifen	Anti-neoplastic agents	0.01-10	
	Cyclophosphamide	Anti-neoplastic agents	0.01-10	
Hospital wastewaters	Metronidazole	Nitroimidazoles	36.5	[25]
	Ciprofloxacin	Quinolones	42.8	
Swine wastewaters	Sulfamethazine	Sulphonamides	0.44-324	[27]
	Tetracycline	Tetracyclines	1.45-388	
	Tylosin	Macrolides	8.6-72	
	17β-Estradiol	Hormones	0.008-0.54	
	17α-Ethynylestradiol	Hormones	0.18-0.36	
Municipal wastewaters	Acetaminophen	Fever and pain treatment medicines	3.6-99.6	[28]
Municipal wastewaters	Diclofenac	NSAIDs	0.88-4	[29]
	Carbamazepine	Anti-epileptic agents	~0.2	
Municipal wastewaters	Ibuprofen	NSAIDs	23	[30]
	Naproxen	NSAIDs	14	
	Triclosan	Disinfectants	1	
Municipal wastewaters	17β-Estradiol	Hormones	0.5	[31]
Sewage	Amoxicillin	β-lactams	≤6.9	[32]
	Sulfamethoxazole	Sulphonamides	≤5.5	
	Ciprofloxacin	Quinolones	≤4.6	
	Doxycycline	Tetracycline	≤2.2	
	Erythromycin	Macrolides	≤10	
Wastewater	Chloramphenicol	Phenicols	3.3	[33]
	Thiamphenicol	Phenicols	1.2	
	Florfenicol	Phenicols	3.3	
	Neomycin	Aminoglycosides	12	
Sewage	Methylparaben	Antiseptic/preservative/disinfectant ingredients	15	[35]
	Isobutylparaben		0.13	
	Butylparaben		0.87	
	Triclosan		3.4	
	Triclocarban		0.11	

Sewage	Amitriptyline	Antidepressants	≤5.1	[36]
	Fluoxetine		≤3.6	
	Sertraline		≤0.11	
	Citalopram		≤0.65	
	Paroxetine		<32.2	
Wastewater	Gemfibrozil	Lipid regulators	0.003	[37]
	Bezafibrate		0.004	
	Fenofibrate		0.0005	
Landfill leachate	Ibuprofen	NSAIDs	167	[38]
	Naproxen	NSAIDs	520	
	Carbamazepine	Anti-epileptic agents	1000	

Table S2. The effect of toxicants (PHRs and PCPs) on the growth of different microalgae and cyanobacteria strains.

Strain	Toxicant	Concentration	Exposure time	Effect on growth	Effect on metabolism	Ref.
			Antibiotics-phenicols			
Pseudokirchneriella subcapitata<br>*Scenedesmus quadricauda*<br>*Scenedesmus obliquus*<br>*Scenedesmus acuminatus*	Chloramphenicol	2.7 mg/L<br>0.89 mg/L<br>1.19 mg/L<br>2.28 mg/L	96 h	A 50% inhibition	Changes in compositions of lipids, proteins and DNA.	[40]
Chlorella pyrenoidosa	Chloramphenicol<br>Florphenicol<br>Thiamphenicol	14 mg/L<br>215 mg/L<br>1283 mg/L	96 h	A 50% reduction	n.d.	[41]
Chlorella pyrenoidosa	Florphenicol	14.2 mg/L (39.7 µM)	96 h	A 50% inhibition	n.d.	[51]
Chlorella sp.	Florfenicol	≥86 mg/L	16-20 days	A 45-100% inhibition	Increase in MDA content and SOD activity.	[52]
Chlorella vulgaris<br>*Selenastrum capricornutum*	Thiamphenicol	522 mg/L<br>8.9 mg/L	72 h	A 50% inhibition	n.d.	[45]
Pseudokirchneriella subcapitata	Florphenicol	2.9 mg/L	72 h	A 50% toxicity	n.d.	[48]
Scenedesmus vacuolatus	Florphenicol	18 mg/L	24 h	A 50% inhibition	n.d.	[50]
Isochrysis galbana	Florphenicol<br>Chloramphenicol<br>Thiamphenicol	8 mg/L<br>41 mg/L<br>158 mg/L	96 h	A 50% reduction	n.d.	[41]
Desmodesmus subspicatus	Chloramphenicol	0.47 mg/L – 1 mg/L	72 h	A 50% toxicity	n.d.	[39]
Isochrysis galbana	Chloramphenicol	12 mg/L	15 days	A 22% inhibition	n.d.	[43]
Tetraselmis chuii	Florphenicol<br>Chloramphenicol<br>Thiamphenicol	1.3 mg/L<br>4 mg/L<br>38 mg/L	96 h	A 50% reduction	n.d.	[41]
Tetraselmis suecica	Chloramphenicol<br>Florphenicol	11 mg/L<br>9 mg/L	96 h	A 50% inhibition	Decrease in Chl *a* content (24 h, at ≥2.5 mg/L)	[42]
Tetraselmis chuii	Florphenicol	6-11 mg/L	72h-96 h	A 50% inhibition	n.d.	[49]

Skeletonema costatum	Florfenicol	5 mg/L	96 h	A 50% inhibition	n.d.	[53]
Chaetoceros gracilis	Chloramphenicol	12 mg/L	15 days	Almost complete growth suppression	n.d.	[43]
Nostoc flagelliforme	Chloromycetin (Chloramphenicol)	≥25 mg/L	12 days	Almost complete growth suppression	Decrease in Chl *a* content	[44]
Microcystis flosaquae	Florfenicol<br>Thiamphenicol	0.05 mg/L (50 µg/L)<br>0.1 mg/L (100 µg/L)	7 days<br>7 days	~50% reduction in chlorophyll concentration	Increase in MDA content. Increase in CAT and SOD activity.	[46]
Anabaena cylindrica<br>*Anabaena flosaquae*<br>*Anabaena variabilis*<br>*Microcystis aeruginosa*<br>*Microcystis wesenbergii*<br>*Nostoc* sp.<br>*Synechococcus leopoldensis*<br>*Synechococcus* sp.	Thiamphenicol	1.3 mg/L<br>13 mg/L<br>14 mg/L<br>0.32 mg/L<br>0.43 mg/L<br>3.5 mg/L<br>0.36 mg/L<br>0.67 mg/L	144 h	A 50% inhibition	n.d.	[47]
			Antibiotics-tetracyclines			
Dictyosphaerium pulchellum<br>*Micractinium pusillum*	Tetracycline	≥10 mg/L<br>10 mg/L<br>20 mg/L	11 days	A 100% inhibition<br>Up to 35% inhibition<br>A ~50% inhibition	n.d.	[54]
Scenedesmus obliquus	Tetracycline	0.28 mg/L (0.63 µM)	72 h	A 50% inhibition	n.d.	[59]
Pseudokirchneriella subcapitata	Tetracycline	1.82 mg/L	72 h	A 50% toxicity	n.d.	[58]
Pseudokirchneriella subcapitata<br>*Anabaena*	Tetracycline	3.3 mg/L<br>6.2 mg/L	72 h	A 50% toxicity	n.d.	[55]
Selenastrum capricornutum<br>*Microcystis aeruginosa*	Tetracycline	2.2 mg/L<br>0.09 mg/L	3 days<br>7 days	A 50% toxicity	n.d.	[56]
Selenastrum capricornutum<br>*Microcystis aeruginosa*	Tetracycline	1 mg/L	7 days	A 43% inhibition<br>A 35% inhibition	Increase in MDA content, increase in SOD activity	[57]
Synechocystis	Tetracycline	0.01-0.1 mg/L (10-100 µg/L)	5 days	A 20% inhibition	n.d.	[62]
Microcystis aeruginosa	Tetracycline	5 mg/L	6 days	A ~45% inhibition	Inhibition of MC-LR production	[60]
Nostoc flagelliforme	Tetracycline	100 mg/L	12 days	A ~50% inhibition	Decrease in Chl a content, increase in exopolysaccharide production	[44]
Microcystis aeruginosa<br>*Aphanizomenon gracile*	Tetracycline	≥0.05 mg/L<br>≥0.0015 mg/L	13 days	A ≥50% inhibition	n.d.	[61]

Chrisosporum berghii<br>*Planktotjrix agardhii*		>0.1-0.2 mg/L<br>0.003 mg/L		A ≥50% inhibition<br>A >50% inhibition<br>A 50% inhibition		
Pseudokirchneriella subcapitata<br>*Ankistrodesmus fusiformis*	Chlortetracycline	1.2 mg/L<br>3.2 mg/L	96 h	A 50% reduction	n.d.	[63]
Selenastrum capricornutum<br>*Microcystis aeruginosa*	Chlortetracycline	3.1 mg/L<br>0.05 mg/L	3 days<br>7 days	A 50% toxicity	n.d.	[56]
Chlorella pyrenoidosa<br>*Microcystis aeruginosa*	Chlortetracycline	37.8 mg/L (73.4 μmol/L)<br>15.2 mg/L (29.5 μmol/L)	96 h	A 50% toxicity	Increase in MDA content, increase in SOD activity.	[64]
Microcystis aeruginosa	Chlortetracycline	20 mg/L	6 days	A 100% inhibition	Inhibition of MC-LR intracellular production	[60]
Tetraselmis suecica	Oxytetracycline	17 mg/L	96 h	A 50% inhibition	Whole cell (at 10 mg/L)	[42]
Tetraselmis chuii	Oxytetracycline	11 mg/L	96 h	A 50% inhibition	n.d.	[49]
Scenedesmus vacuolatus	Oxytetracycline	40 mg/L	24 h	A 50% inhibition	n.d.	[50]
Chlorella vulgaris<br>*Selenastrum capricornutum*	Oxytetracycline	7 mg/L<br>0.34 mg/L	72 h	A 50% inhibition	n.d.	[45]
Pseudokirchneriella subcapitata	Oxytetracycline	2.54 mg/L	72 h	A 50% toxicity	n.d.	[48]
Pseudokirchneriella subcapitata	Oxytetracycline	0.17 mg/L	72 h	A 50% toxicity	n.d.	[65]
Pseudokirchneriella subcapitata<br>*Ankistrodesmus fusiformis*	Oxytetracycline	0.64 mg/L<br>4.17 mg/L	96 h	A 50% reduction	n.d.	[63]
Pseudokirchneriella subcapitata<br>*Microcystis aeruginosa*	Oxytetracycline	0.6 mg/L<br>5.4 mg/L	1 day	A 50% inhibition in photosynthetic efficiency	n.d.	[66]
Pseudokirchneriella subcapitata Anabaena flosaquae	Oxytetracycline	1 mg/L<br>2.7 mg/L	72 h	A 50% toxicity	n.d.	[67]
Selenastrum capricornutum<br>*Rhodomonas salina*<br>*Microcystis aeruginosa*	Oxytetracycline	4.5 mg/L<br>1.6 mg/L<br>0.207 mg/L	-	A 50% toxicity	-	[68]
Microcystis aeruginosa	Oxytetracycline	0.01-1 mg/L	9 days	Up to 19% inhibition	Change in antioxidant activity (SOD, POD, CAT).	[69]
Microcystis aeruginosa	Oxytetracycline	1-10 mg/L	6 days	Up to ~57% inhibition	Interference with toxin production	[60]

Anabaena cylindrica<br>*Anabaena flosaquae*<br>*Anabaena variabilis*<br>*Microcystis aeruginosa*<br>*Microcystis wesenbergii*<br>*Nostoc* sp.<br>*Synechococcus leopoldensis*<br>*Synechococcus* sp.	Oxytetracycline	0.032 mg/L<br>0.39 mg/L<br>0.36 mg/L<br>0.23 mg/L<br>0.35 mg/L<br>7 mg/L<br>1.1 mg/L<br>2 mg/L	144 h	A 50% inhibition	n.d.	[47]
Tetraselmis chuii	Doxycycline	22 mg/L	96 h	A 50% reduction	n.d.	[70]
Pseudokirchneriella subcapitata	Doxycycline	0.33 mg/L	72 h	A 50% toxicity	n.d.	[48]
Microcystis aeruginosa	Doxycycline	1 mg/L<br>1 mg/L	24 h<br>96 h	A 8% inhibition<br>A 55% inhibition	Increase in MDA content, increase in CAT activity<br>n.d	[71]
Microcystis aeruginosa	Minocycline	0.45 mg/L (0.92 μM)	6-10 days	A 50% inhibition	n.d.	[72]
		Antibiotics-aminoglycosides				
Chlorella vulgaris	Streptomycin	2.4 mg/L	72 h	A 40% inhibition	Decrease in D1 protein	[73]
Selenastrum capricornutum<br>*Microcystis aeruginosa*	Streptomycin	0.133 mg/L<br>0.007 mg/L	3 days<br>7 days	A 50% inhibition	n.d.	[56]
Pseudokirchneriella subcapitata<br>*Microcystis aeruginosa*	Streptomycin	1.5 mg/L<br>0.034 mg/L	24 h	50% inhibition in photosynthetic efficiency	n.d.	[66]
Dictyosphaerium pulchellum<br>*Micractinium pusillum*	Kanamycin	5-30 mg/L	11 days	Complete inhibition<br>A 25%(at 5 mg/L)-100%(at 30 mg/L) inhibition	n.d.	[54]
Microcystis aeruginosa<br>*Aphanizomenon gracile*<br>*Planktotjrix agardhii*<br>*Chrisosporum berghii*	Kanamycin	0.1-1.6 mg/L<br>≥0.2 mg/L	13 days	≥50% growth inhibitions	n.d.	[61]
Pseudokirchneriella subcapitata	Gentamicin	19.2 mg/L	72 h	A 50% inhibition	n.d.	[75]
Microcystis aeruginosa<br>*Aphanizomenon gracile*<br>*Planktotjrix agardhii*	Gentamicin	0.1-1.6 mg/L	13 days	≥50% growth inhibitions	n.d.	[61]

Chrisosporum berghii		≥0.2 mg/L				
Nostoc flagelliforme	Gentamicin (sulfate)	≥25 mg/L	12 days	Complete growth suppression	Increase in exopolysaccharide production	[44]
	Spectinomycin	≥5 mg/L			Decrease in Chl *a* content	
	Streptomycin (sulfate)	≥1 mg/L			Decrease in Chl *a* content and increase in exopolysaccharide production	
	Kanamycin (sulfate)	1-100 mg/L		Up to 27% growth inhibition	Increase in exopolysaccharide production	
Microcystis aeruginosa<br>*Lyngbya* sp.<br>*Oscillatoria tenuis*<br>*Anabaena cylindrica*<br>*Anabaena flosaquae*<br>*Aphanizomenon flosaquae*<br>*Chlamydomonas reinhardtii*<br>*Pediastrum* sp.<br>*Chlorella vulgaris*<br>*Ankistrodesmus* sp.<br>*Selenastrum capricornutum*<br>*Scenedesmus obliquus*<br>*Ulothrix* sp.<br>*Stigeoclonium* sp.<br>*Navicula* sp.	Streptomycin sulfate	0.28 mg/L<br>0.09 mg/L<br>0.28 mg/L<br>0.28 mg/L<br>0.28 mg/L<br>0.86 mg/L<br>0.66 mg/L<br>2.1 mg/L<br>66 mg/L<br>6.6 mg/L<br>2.1 mg/L<br>21 mg/L<br>21 mg/L<br>6.6 mg/L<br>6.6 mg/L	-	MIC (Minimum Inhibitory Concentration)	n.d.	[74]
		Antibiotics-sulphonamides				
Chlorella vulgaris	Sulfamethoxazole<br>Sulfapyridine<br>Sulfadimethoxine	0.98-1.51 mg/L<br>1-1.93 mg/L<br>5.19-7.65 mg/L	48-72 h	A 50% inhibition	n.d.	[78]
Chlorella vulgaris	Sulfadimethoxine	11.2 mg/L	72 h	A 50% inhibition	n.d.	[45]
Chlorella vulgaris	Sulfacetamide	14.6 mg/L (0.062 mM)	48 h	A 50% inhibition	n.d.	[79]
	Sulfamethoxazole	1.57 mg/L (0.0062 mM)				
	Sulfadiazine	1.33 mg/L (0.0049 mM)				
	Sulfathiazole	17.74 mg/L (0.064 mM)				
Scenedesmus vacuolatus	Sulfamethoxazole<br>Sulfapyridine<br>Sulfadimethoxine<br>Sulfamerazine	1.54 mg/L<br>5.28 mg/L<br>9.85 mg/L<br>11.9 mg/L	24 h	A 50% inhibition	n.d.	[76]

	Sulfadimidine (sulfamethazine)	19.52 mg/L				
Scenedesmus obliquus	Sulfamethoxazole<br>Sulfamethazine	0.12 mg/L<br>1.23 mg/L	96 h	A 50% inhibition	n.d.	[77]
Desmodesmus subspicatus	Sulfamethoxazole	0.25 mg/L (250 μg/L)	-	Complete inhibition in photosynthetic activity	n.d.	[83]
Desmodesmus subspicatus	Sulfamethoxazole	210 mg/L	-	A 50% inhibition in photosynthetic activity	n.d.	[84]
Chlorella pyrenoidosa	Sulfamethazine	8 mg/L	5 days	A 45% inhibition	Increase in SOD, CAT and GST activity	[85]
Selenastrum capricornutum	Sulfamethoxazole	2.5 mg/L	96 h	A ~25% growth inhibition	Decrease in Chl *a* content	[80]
Selenastrum capricornutum	Sulfadimethoxine<br>Sulfamethoxazole<br>Sulfadiazine	2.3 mg/L<br>1.5 mg/L<br>2.2 mg/L	72 h	A 50% inhibition	n.d.	[45]
Phaeodactylum tricornutum<br>*Isochrysis galbana*	Sulfadiazine	0.11 mg/L<br>1.44 mg/L	96 h	A 50% inhibition	n.d.	[87]
Selenastrum capricornutum<br>*Rhodomonas salina*<br>*Microcystis aeruginosa*	Sulfadiazine	7.8 mg/L<br>403 mg/L<br>0.135 mg/L	-	A 50% toxicity	-	[68]
Chlorella vulgaris<br>*Isochrysis galbana*	Sulfamonomethoxine	5.9 mg/L<br>9.7 mg/L	72 h	A 50% toxicity	n.d.	[88]
Pseudokirchneriella subcapitata	Sulfamonomethoxine	1.34 mg/L	72 h	A 50% toxicity	n.d.	[48]
Pseudokirchneriella subcapitata	Sulfamethoxazole	0.52 mg/L	72 h	A 50% toxicity	n.d.	[65]
Pseudokirchneriella subcapitata	Sulfamethoxazole<br>Sulfamethazine	1.9 mg/L<br>8.7 mg/L	72 h	A 50% inhibition	n.d.	[81]
Microcystis aeruginosa<br>*Pseudokirchneriella subcapitata*	Sulfamethoxazole	0.55 mg/L<br>>9 mg/L	24 h	50% inhibition in photosynthetic efficiency	n.d.	[66]
Pseudokirchneriella subcapitata<br>*Cyclotella meneghiniana*<br>*Synechococcus leopolensis*	Sulfamethoxazole	0.146 mg/L<br>2.4 mg/L<br>0.027 mg/L	96 h	A 50% toxicity	n.d.	[82]
Anabaena cylindrica<br>*Anabaena flosaquae*<br>*Anabaena variabilis*<br>*Microcystis aeruginosa*<br>*Microcystis wesenbergii*	Sulfadimethoxine	480 mg/L<br>>2000 mg/L<br>1500 mg/L<br>500 mg/L<br>470 mg/L	144 h	A 50% inhibition	n.d.	[47]

Nostoc sp.<br>*Synechococcus leopoldensis*<br>*Synechococcus* sp.		>2000 mg/L<br>1100 mg/L<br>760 mg/L				
Spirulina platensis	Sulfamethazine	6 mg/L	48 h	A 50% inhibition	n.d.	[86]
		Antibiotics-trimethoprim				
Pseudokirchneriella subcapitata	Trimethoprim	40 mg/L	72 h	A 50% inhibition	n.d.	[81]
Pseudokirchneriella subcapitata	Trimethoprim	84 mg/L	96 h	A 50% inhibition	n.d.	[89]
Selenastrum capricornutum	Trimethoprim	80 mg/L	72 h	A 50% inhibition	n.d.	[45]
Chlorella vulgaris	Trimethoprim	90 mg/L<br>123 mg/L	72 h<br>48 h	A 50% inhibition	n.d.	[78]
Navicula pelliculosa<br>*Phaeodactylum tricornutum*<br>*Anabaena flosaquae*	Trimethoprim	2.13 mg/L [7.36 μmol/L]<br>21.6 mg/L [74.6 μmol/L]<br>83 mg/L [285 μmol/L]	96 h	A 50% inhibition	n.d.	[91]
Phaeodactylum tricornutum	Trimethoprim	5.1 mg/L	72 h	A 50% inhibition	n.d.	[90]
Aphanizomenon gracile	Trimethoprim	0.003-1.6 mg/L	13 days	Up to 50% inhibition	n.d.	[61]
Anabaena cylindrica<br>*Anabaena flosaquae*<br>*Anabaena variabilis*<br>*Microcystis aeruginosa*<br>*Microcystis wesenbergii*<br>*Nostoc* sp.<br>*Synechococcus leopoldensis*<br>*Synechococcus* sp.	Trimethoprim	>200 mg/L<br>>200 mg/L<br>11 mg/L<br>150 mg/L<br>>200 mg/L<br>53 mg/L<br>>200 mg/L<br>>200 mg/L	144 h	A 50% inhibition	n.d.	[47]
Pseudokirchneriella subcapitata<br>*Anabaena flosaquae*	Trimethoprim	129 mg/L<br>253 mg/L	72 h	A 50% toxicity	n.d.	[67]
Pseudokirchneriella subcapitata<br>*Microcystis aeruginosa*	Trimethoprim	>9 mg/L<br>6.9 mg/L	24 h	A 50% inhibition in photosynthetic efficiency	n.d.	[66]
Selenastrum capricornutum<br>*Rhodomonas salina*<br>*Microcystis aeruginosa*	Trimethoprim	130 mg/L<br>16 mg/L<br>112 mg/L	-	A 50% toxicity	-	[68]
Desmodesmus subspicatus	Trimethoprim	0.0078-0.125 mg/L<br>(7.8-125 μg/L)	-	A 34%±6% inhibition in photosynthetic efficiency	n.d.	[83]
		Antibiotics-β-lactams				
		Antibiotics-β-lactams-penicillins				

Microcystis aeruginosa<br>*Selenastrum capricornutum*	Benzylpenicillin (Penicillin G)	0.006 mg/L<br>100 mg/L	7 days<br>3 days	A 50% inhibition<br>No inhibition	n.d.	[56]
Pseudokirchneriella subcapitata	Penicillin G	7114 mg/L	72 h	A 50% toxicity	n.d.	[58]
Antibiotics-β-lactams-aminopenicillins						
Anabaena cylindrica<br>*Anabaena flosaquae*<br>*Anabaena variabilis*<br>*Microcystis aeruginosa*<br>*Microcystis wesenbergii*<br>*Nostoc* sp.<br>*Synechococcus leopoldensis*<br>*Synechococcus* sp.	Ampicillin	0.14 mg/L<br>3.3 mg/L<br>2.2 mg/L<br>0.0002 mg/L<br>0.013 mg/L<br>>200 mg/L<br>0.083 mg/L<br>0.0069 mg/L	144 h	A 50% inhibition	n.d.	[47]
Microcystis aeruginosa<br>*Aphanizomenon gracile*<br>*Chrisosporum berghii*<br>*Planktotjrix agardhii*	Amoxicillin	≥0.006 mg/L<br>≥0.0015 mg/L<br>≥0.0015 mg/L<br>≥0.006 mg/L	13 days	A ≥50% inhibition	n.d.	[61]
Anabaena sp.	Amoxicillin	56 mg/L	72 h	A 50% toxicity	n.d.	[55]
Synechococcus leopolensis	Amoxicillin	2.2 μg/L	96 h	A 50% toxicity	n.d.	[92]
Nostoc flagelliforme	Ampicillin	25-50 mg/L<br>100 mg/L	12 days	A 55% inhibition<br>100% inhibition	Stimulation in exopolysaccharides production (at ≥25 mg/L)	[44]
Selenastrum capricornutum<br>*Rhodomonas salina*<br>*Microcystis aeruginosa*	Amoxicillin	NOEC >250 mg/L<br>3108 mg/L<br>0.0037 mg/L	-	A 50% toxicity	-	[68]
Pseudokirchneriella subcapitata	Amoxicillin Ampicillin	2000 mg/L (2 g/L)	72 h	No inhibition	n.d.	[75]
Pseudokirchneriella subcapitata	Amoxicillin	1500 mg/L (1.5 g/L)	72 h	No toxicity	n.d.	[55]
Pseudokirchneriella subcapitata	Amoxicillin	~500 mg/L	48 h	A 50% toxicity	n.d.	[48]
Selenastrum capricornutum<br>*Chlorella vulgaris*	Ampicillin	1000 mg/L (1 g/L)	72 h	No negative influence	n.d.	[45]
Antibiotics-β-lactams-cephalosporins						
Microcystis aeruginosa<br>*Aphanizomenon gracile*<br>*Chrisosporum berghii*<br>*Planktotjrix*	Ceftazidime	≥0.05 mg/L<br>≥0.006 mg/L<br>≥0.05 mg/L	13 days	A ≥50% inhibition	n.d.	[61]

agardhii		≥0.1-0.2 mg/L				
Microcystis aeruginosa<br>*Aphanizomenon gracile*<br>*Chrisosporum berghii*<br>*Planktotjrix agardhii*	Ceftriaxone	≥0.1-0.2 mg/L<br>≥0.003 mg/L<br>≥0.012 mg/L<br>≥0.05-0.1 mg/L	13 days	A ≥50% inhibition	n.d.	[61]
Microcystis aeruginosa	Cefradine	≥3 mg/L	6 days	Complete growth inhibition	n.d.	[94]
Scenedesmus obliquus	Cefradine	12 mg/L	12 days	A ~50% inhibition	n.d.	[94]
Scenedesmus sp.	Ceftiofur<br>Cefapirin	53.4 mg/L<br>43.6 mg/L	72 h	No effect	n.d.	[93]
Pseudokirchneriella subcapitata	Cefotaxime	430 mg/L	72 h	A 50% toxicity	n.d.	[48]
Pseudokirchneriella subcapitata	Cephalothin	600 mg/L	72 h	A 48% growth inhibition	n.d.	[75]
Selenastrum capricornutum	Cefazolin	1000 mg/L (1 g/L)	72 h	No negative influence	n.d.	[45]
			Antibiotics-quinolones			
Chlamydomonas mexicana<br>*Scenedesmus obliquus*<br>*Chlorella vulgaris*<br>*Ourococcus multisporus*<br>*Micractinium resseri*	Enrofloxacin	10.8 mg/L<br>9.86 mg/L<br>12.2 mg/L<br>15 mg/L<br>12 mg/L	96 h	A 50% inhibition	Changes in pigment content. Increase in MDA content.	[95]
Scenedesmus obliquus	Enrofloxacin	38-53 mg/L (different strains)	72 h	A 50% inhibition	n.d.	[96]
Scenedesmus obliquus	Enrofloxacin	45-88 mg/L	24-96 h	A 50% inhibition	For 96 h exposure: increase in protein content (at 24-96 mg/L), increase in MDA and proline content (at 80-120 mg/L).	[97]
Pseudokirchneriella subcapitata<br>*Ankistrodesmus fusiformis*	Enrofloxacin	5.18 mg/L<br>10.6 mg/L	96 h	A 50% inhibition	n.d.	[98]
Microcystis aeruginosa	Enrofloxacin	0.031-0.054 mg/L (different strains)	72 h	A 50% inhibition	n.d.	[96]
Scenedesmus obliquus	Norfloxacin	38.5 mg/L	96 h	A 50% inhibition	Changed catalase (CAT) activity, increased MDA content.	[99]
Pseudokirchneriella subcapitata	Norfloxacin	18 mg/L	72 h	A 50% inhibition	n.d.	[81]
Pseudokirchneriella subcapitata	Norfloxacin	56.4 mg/L	72 h	A 50% toxicity	n.d.	[48]
Pseudokirchneriella subcapitata<br>*Anabaena*	Norfloxacin	>80 mg/L<br>5.6 mg/L	72 h	A 50% toxicity	n.d.	[55]
Anabaena						

cylindrica<br>*Anabaena flosaquae*<br>*Anabaena variabilis*<br>*Microcystis aeruginosa*<br>*Microcystis wesenbergii*<br>*Nostoc* sp.<br>*Synechococcus leopoldensis*<br>*Synechococcus* sp.	Norfloxacin	0.053 mg/L<br>0.29 mg/L<br>0.19 mg/L<br>0.062 mg/L<br>0.038 mg/L<br>1.7 mg/L<br>0.63 mg/L<br>0.63 mg/L	144 h	A 50% inhibition	n.d.	[47]
Microcystis aeruginosa<br>*Aphanizomenon gracile*<br>*Chrisosporum berghii*<br>*Planktotjrix agardhii*	Norfloxacin	>0.006 mg/L<br>≥0.025 mg/L<br>≥0.1-0.2 mg/L<br>≥0.8 mg/L	13 days	A ≥50% inhibition	n.d.	[61]
Chlorella vulgaris	Levofloxacin	58.6 mg/L	96 h	A 50% inhibition	n.d.	[100]
Scenedesmus obliquus	Levofloxacin	65 mg/L	96 h	A 50% inhibition	n.d.	[101]
Pseudokirchneriella subcapitata<br>*Anabaena*	Levofloxacin	> 120 mg/L<br>4.8 mg/L	72 h	A 50% toxicity	n.d.	[55]
Microcystis flosaquae	Levofloxacin	≥0.04 mg/L (≥40 μg/L)	7 days	Complete growth inhibition	Increase in MDA content. Increase in CAT and SOD activity.	[102]
Chlorella vulgaris	Ciprofloxacin	27-40 mg/L	48-96h	A 50% inhibition	n.d.	[104]
Chlorella vulgaris	Ciprofloxacin	20.6 mg/L	96h	A 50% growth reduction	Decrease in total glutathione (GSH), increase in catalase (CAT) activity	[105]
Chlorella vulgaris	Ciprofloxacin	31 mg/L	72/96h	A 50% inhibition	n.d.	[106]
Chlorella pyrenoidosa	Ciprofloxacin	100 mg/L - 150 mg/L	8 days	A ≥30% inhibition	The whole cell	[107]
Chlamydomonas mexicana	Ciprofloxacin	65 mg/L	96 h	A 50% inhibition	Increase in total *Chl*, total *Car*, MDA content and SOD activity (at 60 mg/L)	[108]
Selenastrum capricornutum	Ciprofloxacin	2.5 mg/L	96 h	A 40% inhibition	Decrease in Chl *a*, Chl *b* and Total *Car* content.	[80]
Pseudokirchneriella subcapitata	Ciprofloxacin	6.7 mg/L	72 h	A 50% inhibition	n.d.	[81]
Pseudokirchneriella subcapitata	Ciprofloxacin	11.3 mg/L	72 h	A 50% inhibition	n.d.	[75]
Pseudokirchneriella subcapitata	Ciprofloxacin	8.8 mg/L	72 h	A 50% toxicity	n.d.	[48]
Scenedesmus obliquus<br>*Raphidocelis subcapitata*<br>*Desmodesmus quadricauda*	Ciprofloxacin	6.8 mg/L<br>6.7 mg/L<br>5.2 mg/L	72 h	A 50% toxicity	n.d.	[103]

Microcystis aeruginosa		0.02 mg/L				
Cylindrotheca closterium *Navicula ramosissima*	Ciprofloxacin	55 mg/L 72 mg/L	-	A 50% inhibition	n.d.	[109]
Selenastrum capricornutum *Rhodomonas salina* *Microcystis aeruginosa*	Flumequine	5 mg/L 18 mg/L 0.159 mg/L	-	A 50% toxicity	-	[68]
Selenastrum capricornutum *Rhodomonas salina* *Microcystis aeruginosa*	Oxolinic acid	16 mg/L 10 mg/L 0.18 mg/L	-	A 50% toxicity	-	[68]
Selenastrum capricornutum *Rhodomonas salina* *Microcystis aeruginosa*	Sarafloxacin	16 mg/L 24 mg/L 0.015 mg/L	-	A 50% toxicity	-	[68]
Pseudokirchneriella subcapitata	Ofloxacin	5.3 mg/L	72 h	A 50% toxicity	n.d.	[48]
Pseudokirchneriella subcapitata	Ofloxacin	1.44 mg/L	72 h	A 50% toxicity	n.d.	[65]
Pseudokirchneriella subcapitata *Cyclotella meneghiniana* *Synechococcus leopolensis*	Ofloxacin	4.7 mg/L 0.09 mg/L 0.016 mg/L	96 h	A 50% toxicity	n.d.	[82]
			Antibiotics-macrolides			
Chlorella vulgaris *Ankestrodesmus falcatus*	Erythromycin	12 mg/L 10 mg/L	24 h	A 50% toxicity	Decrease in SOD and CAT activity	[112]
Selenastrum capricornutum	Erythromycin	0.3 mg/L	96 h	A 90% inhibition	Decrease in Chl *a*, Chl *b*	[80]
Pseudokirchneriella subcapitata	Erythromycin	0.124 mg/L (170 nM)	72 h	A 50% inhibition	n.d.	[110]
Pseudokirchneriella subcapitata	Erythromycin	0.02 mg/L	72 h	A 50% toxicity	n.d.	[65]
Pseudokirchneriella subcapitata *Anabaena*	Erythromycin	0.35 mg/L 0.022 mg/L	72 h	A 50% toxicity	n.d.	[55]
Chlamydomonas reinhardtii *Phaeodactylum tricornutum*	Erythromycin	0.36 mg/L 1.31 mg/L	72 h	A 50% inhibition	Whole cell	[111]
Chaetoceros gracilis	Erythromycin	1 mg/L 9 mg/L	15 days	A ~50% inhibition A 100% inhibition	n.d.	[43]
Pseudanabaena planctonica (wild type)	Erythromycin	10 mg/L (10 μg/mL)	3 days	No growth detected	n.d.	[114]

Microcystis flosaquae	Erythromycin	0.04 mg/L (40 μg/L)	8 days	An 82% inhibition	Increase in MDA content. Increase in SOD and CAT activity.	[113]
Anabaena cylindrica<br>*Anabaena flosaquae*<br>*Anabaena variabilis*<br>*Microcystis aeruginosa*<br>*Microcystis wesenbergii*<br>*Nostoc* sp.<br>*Synechococcus leopoldensis*<br>*Synechococcus* sp.	Erythromycin	0.035 mg/L<br>0.27 mg/L<br>0.43 mg/L<br>0.023 mg/L<br>0.023 mg/L<br>0.2 mg/L<br>0.16 mg/L<br>0.23 mg/L	144 h	A 50% inhibition	n.d.	[47]
Pseudokirchneriella subcapitata	Clarithromycin	0.002 mg/L	72 h	A 50% toxicity	n.d.	[65]
Pseudokirchneriella subcapitata	Clarithromycin	0.046 mg/L	72 h	A 50% inhibition	n.d.	[81]
Pseudokirchneriella subcapitata	Clarithromycin	0.23 mg/L	72 h	A 50% inhibition	n.d.	[115]
Pseudokirchneriella subcapitata	Clarithromycin	0.018 mg/L (24 nM	72 h	A 50% inhibition	n.d.	[110]
Pseudokirchneriella subcapitata	Clarithromycin	0.0069 mg/L	72 h	A 50% inhibition	n.d.	[116]
Desmodesmus subspicatus<br>*Anabaena flosaquae*	Clarithromycin	0.03 mg/L (32-37 μg/L)<br>0.005-0.012 mg/L (5.6-12 μg/L)	72 h	A 50% inhibition	n.d.	[117]
Selenastrum capricornutum<br>*Microcystis aeruginosa*	Spiramycin	2.3 mg/L<br>0.005 mg/L	3 days<br>7 days	A 50% inhibition	n.d.	[56]
Microcystis aeruginosa	Spiramycin	0.0011 mg/L	7 days	A 50% inhibition	n.d.	[118]
Pseudokirchneriella subcapitata	Roxithromycin	0.047 mg/L	72 h	A 50% inhibition	n.d.	[81]
Pseudokirchneriella subcapitata<br>*Scenedesmus quadricauda*<br>*Scenedesmus obliquus*<br>*Scenedesmus acuminatus*	Roxithromycin	0.73 mg/L<br>0.129 mg/L<br>0.077 mg/L<br>2.87 mg/L	96 h	A 50% inhibition	Changes in compositions of lipids, proteins and DNA.	[40]
Pseudokirchneriella subcapitata	Azithromycin	0.005 mg/L	72 h	A 50% toxicity	n.d.	[48]
Pseudokirchneriella subcapitata	Tylosin	0.21 mg/L	72 h	A 50% inhibition	n.d.	[81]
Selenastrum capricornutum<br>*Microcystis aeruginosa*	Tylosin	1.38 mg/L<br>0.034 mg/L	3 days<br>7 days	A 50% inhibition	n.d.	[56]
Pseudokirchneriella subcapitata<br>*Microcystis aeruginosa*	Tylosin	0.0089 mg/L<br>0.29 mg/L	24 h	A 50% inhibition in photosynthetic efficiency	n.d.	[66]

Pseudokirchneriella subcapitata<br>*Desmodesmus subspicatus*<br>*Navicula pelliculosa*<br>*Phaeodactylum tricornutum*<br>*Anabaena flosaquae*<br>*Synechococcus leopoliensis*	Tylosin	4.41 mg/L (4.14 μmol/L)<br>13 mg/L (12.19 μmol/L)<br>1.42 mg/L (1.33 μmol/L)<br>6.07 mg/L (5.7 μmol/L)<br>0.098 mg/L (0.092 μmol/L)<br>0.096 mg/L (0.09 μmol/L)	4 days	A 50% inhibition	n.d.	[91]
Cylindrotheca closterium<br>*Navicula ramosissima*	Tylosin	0.27 mg/L<br>0.99 mg/L	-	A 50% inhibition	n.d.	[109]
Euglena gracilis<br>*Chlorella vulgaris*<br>*Scenedesmus obliquus,*<br>*Chlamydomonas reinhardtii*<br>*Ochromonas malhamensis*<br>*Plectonema boryanum*<br>*Navicula pelliculosa*	Nystatin	30 mg/L (30 μg/mL)<br>3 mg/L (3 μg/mL)<br>1 mg/L (1 μg/mL)<br>1 mg/L (1 μg/mL)<br>1 mg/L (1 μg/mL)<br>1 mg/L (1 μg/mL)<br>>60 mg/L (>60 μg/mL)	72 h	Minimal inhibitory concentration	K^+ leakage from *Chlorella vulgaris* cells.	[119]
		Antibiotics-glycopeptides				
Pseudokirchneriella subcapitata	Vancomycin	724 mg/L	72 h	A 50% inhibition	n.d.	[75]
Pseudokirchneriella subcapitata	Vancomycin	371 mg/L	72 h	A 50% toxicity	n.d.	[58]
		Antibiotics-lincosamides				
Cylindrotheca closterium<br>*Navicula ramosissima*	Lincomycin	14 mg/L<br>11 mg/L	-	A 50% inhibition	n.d.	[109]
Cyclotella meneghiniana<br>*Pseudokirchneriella subcapitata*<br>*Synechococcus leopoliensis*	Lincomycin	1.6 mg/L<br>1.5 mg/L<br>0.195 mg/L	96 h	A 50% toxicity	n.d.	[120]
Desmodesmus subspicatus<br>*Pseudokirchneriella subcapitata*<br>*Anabaena flosaquae*<br>*Synechococcus leopoliensis*	Lincomycin	7.08 mg/L (16 μmol/L)<br>3.26 mg/L (7.36 μmol/L)<br>0.057 mg/L (0.13 μmol/L)<br>0.042 mg/L (0.095 μmol/L)	4 days	A 50% inhibition	n.d.	[91]
Pseudokirchneriella subcapitata	Lincomycin	0.07 mg/L	72 h	A 50% toxicity	n.d.	[65]
Pseudokirchneriella subcapitata	Clindamycin	0.01mg/L	72 h	A 50% inhibition	n.d.	[115]
		Antibiotics-nitrofurans				

Desmodesmus subspicatus	Nitrofurantoin	12-17 mg/L	24-72 h	A 50% inhibition	n.d.	[121]
Desmodesmus subspicatus	Nitrofurantoin	0.5 mg/L (500 μg/L)	-	A 70% inhibition in photosynthetic activity	n.d.	[83]
Isochrysis galbana *Chaetoceros gracilis*	Furazolidone	0.5 mg/L	15 days	Complete growth inhibition	n.d.	[43]
Chlorella pyrenoidosa	Furazolidone	1.3 mg/L	2 days	A 50% inhibition	n.d.	[122]
			Antibiotics-nitroimidazoles			
Desmodesmus subspicatus	Metronidazole	0.0078-0.125 mg/L (7.8-125 μg/L)	-	A 50%±4% inhibition of photosynthetic activity	n.d.	[83]
Scenedesmus vacuolatus	Metronidazole	705 mg/L	24 h	A 50% inhibition	n.d.	[50]
Chlorella sp.<br>*Selenastrum capricornutum*	Metronidazole	12.5 mg/L-38.8 mg/L<br>40.4 mg/L	72 h	A 50% toxicity	n.d.	[123]
Pseudokirchneriella subcapitata	Metronidazole	56.6 mg/L	72 h	A 50% toxicity	n.d.	[48]
Microcystis protocystis	Metronidazole	117 mg/L	96 h	A 50% inhibition	n.d.	[124]
			Antibiotics-quinoxalines			
Selenastrum capricornutum<br>*Microcystis aeruginosa*	Olaquindox	40 mg/L<br>5 mg/L	3 days<br>7 days	A 50% inhibition	n.d.	[56]
Pseudokirchneriella subcapitata	Quinocetone	1.72 mg/L	72 h	A 50% toxicity	n.d.	[48]
			Antibiotics-ansamycins			
Pseudokirchneriella subcapitata	Rifampicin	171 mg/L	72 h	A 50% toxicity	n.d.	[48]
Anacystis montana	Rifamycin B<br>Rifamycin S<br>Rifampicin	9 mg/L (12 μM)<br>4.17 mg/L (6 μM)<br>9.87 mg/L (12 μM)	Up to 72 h	Complete growth inhibition	Inhibition of nucleoside incorporation into RNA, and aminoacid incorporation into protein fractions	[125]
Chlorella pyrenoidosa	Rifamycin B<br>Rifamycin S<br>Rifampicin	18 mg/L(24 μM)<br>16.7 mg/L (24 μM)<br>19.75 mg/L (24 μM)		No growth affected	Cell bleaching (at 12 μM)	
			Antibiotics-pleuromutilin derivatives			
Selenastrum capricornutum<br>*Microcystis aeruginosa*	Tiamulin	0.165 mg/L<br>0.003 mg/L	3 days<br>7 days	A 50% inhibition	n.d.	[56]
			Non-steroidal anti-inflammatory drugs (NSAIDs)			
Chlorella vulgaris	Ibuprofen	71-89 mg/L	96 h	A 50% inhibition	n.d.	[104]
Chlorella sp.	Ibuprofen	≥100 mg/L (≥0.1 g/L)	48 h	100% death cells	n.d.	[126]

Desmodesmus subspicatus	Ibuprofen	340 mg/L (0.34 g/L)	n.d.	A 50% inhibition	n.d.	[127]
Desmodesmus subspicatus	Ibuprofen	92 mg/L	-	A 50% inhibition in photosynthetic activity	n.d.	[84]
Pseudokirchneriella subcapitata	Ibuprofen	230 mg/L (0.23 g/L)	3-4 days	A 50% toxicity	n.d.	[128]
Acutodesmus obliquus<br>*Chlamydomonas reinhardtii*<br>*Nannochloropsis limnetica*	Ibuprofen	288 mg/L (0.288 g/L)<br>622 mg/L (0.622 g/L)<br>965 mg/L (0.965 g/L)	5 h	A 50% toxicity	n.d.	[129]
Navicula sp.	Ibuprofen	≥50 mg/L	2 days	Complete growth suppresion	Inhibition of photosynthesis	[130]
Chlorella vulgaris<br>*Ankestrodesmus falcatus*	Naproxen	40-42 mg/L	24 h	A 50% toxicity	n.d.	[112]
Desmodesmus subspicatus	Naproxen	620 mg/L (0.62 g/L)	n.d.	A 50% inhibition	n.d.	[127]
Pseudokirchneriella subcapitata	Naproxen	44.4 mg/L	72 h	A 50% inhibition	n.d.	[115]
Pseudokirchneriella subcapitata	Naproxen	32 mg/L	96 h	A 50% inhibition	n.d.	[131]
Scenedesmus quadricauda	Naproxen	100 mg/L	24 h	A 100% inhibition	n.d. (24 h)	[132]
Anabaena flosaquae	Naproxen	12.3 mg/L	72 h	A 50% biomass inhibition	n.d.	[133]
Desmodesmus subspicatus	Diclofenac	72 mg/L	-	A 50% inhibition	n.d.	[127]
Desmodesmus subspicatus	Diclofenac	60 mg/L	72 h	A 50% inhibition	n.d.	[134]
Desmodesmus subspicatus	Diclofenac	147.85 mg/L	-	A 50% inhibition in photosynthetic activity	n.d.	[84]
Haematococcus pluvialis	Diclofenac	29-53 mg/L	96 h – 14 days	A 50% inhibition in vegetative cell number	Change in pigment (*Chl*, *Car*) content	[135]
Chlamydomonas reinhardtii	Diclofenac	134 mg/L	24 h	A 50% inhibition	Change in pigment (*Chl b*, *Car*) content	[138]
Pseudokirchneriella subcapitata	Diclofenac	65 mg/L	72 h	A 50% inhibition	n.d.	[136]
Pseudokirchneriella subcapitata	Diclofenac	10 mg/L	96 h	A 50% inhibition	n.d.	[137]
Pseudokirchneriella subcapitata	Diclofenac	21 mg/L	72 h	A 50% inhibition	n.d.	[115]
Pseudokirchneriella subcapitata<br>*Cyclotella meneghiniana*<br>*Synechococcus leopolensis*	Diclofenac	16.3 mg/L<br>19.2 mg/L<br>14.5 mg/L	96 h	A 50% toxicity	n.d.	[82]
Chlorella pyrenoidosa	Diclofenac	60 mg/L - 150 mg/L	8 days	A ≥30% inhibition	The whole cell	[107]

Dunaliella tertiolecta	Diclofenac	185 mg/L	96 h	A 50% toxicity	n.d.	[139]
Pseudokirchneriella subcapitata	Ketoprofen	49 mg/L	72 h	A 50% inhibition	n.d.	[115]
Pseudokirchneriella subcapitata	Ketoprofen	24.6 mg/L	72 h	A 50% inhibition	n.d.	[116]
Pseudokirchneriella subcapitata	Etodolac	102 mg/L	72 h	A 50% inhibition	n.d.	[116]
Desmodesmus subspicatus	Acetylsalicylic acid	106 mg/L	-	A 50% inhibition	n.d.	[127]
Pseudokirchneriella subcapitata	Acetylsalicylic acid	241 mg/L	48 h	A 50% inhibition	n.d.	[140]
Pseudokirchneriella subcapitata	Salicylic acid	23.7 mg/L	72 h	A 50% inhibition	n.d.	[141]
Phaeodactylum tricornutum	Salicylic acid	255 mg/L	72 h	A 50% inhibition	n.d.	[90]
			Fever and pain treatment medicines			
Pseudokirchneriella subcapitata<br>*Scenedesmus dimorphus*<br>*Stichococcus bacillaris*<br>*Chlorella vulgaris*<br>*Chlamydomonas reinhardtii*	Paracetamol (acetaminophen)	>240 mg/L	96 h	A 50% inhibition	n.d.<br>n.d.<br>n.d.<br>Increased car: chl a ratio<br>n.d.	[142]
Chlorella vulgaris	Acetaminophen	88 mg/L	72-96 h	A 50% inhibition	n.d.	[106]
Scenedesmus subspicatus	Acetaminophen	134 mg/L	72 h	A 50% inhibition	n.d.	[143]
Pseudokirchneriella subcapitata	Acetaminophen	88.7 mg/L	72 h	No inhibition	n.d.	[116]
Phaeodactylum tricornutum	Paracetamol	266 mg/L	72 h	A 50% inhibition	n.d.	[90]
			Antidepressants			
Pseudokirchneriella subcapitata	Fluoxetine	0.024-0.039 mg/L (24-39 μg/L)	120 h	A 50% inhibition	n.d.	[145]
Pseudokirchneriella subcapitata	Fluoxetine	0.09 mg/L (90 μg/L)	24 h	A 50% toxicity	n.d.	[146]
Chlorella vulgaris<br>*Ankestrodesmus falcatus*	Fluoxetine	0.036-0.04 mg/L (36-40 μg/L)	24 h	A 50% toxicity	Decrease in SOD and CAT activity	[112]
Acutodesmus obliquus<br>*Chlamydomonas reinhardtii*	Fluoxetine	5 mg/L<br>1.6 mg/L	5 h	A 50% toxicity	n.d.	[129]
Dunaliella tertiolecta	Fluoxetine	0.169 mg/L (169 μg/L)	96 h	A 50% toxicity	n.d.	[139]
Skeletonema pseudocostatum	Fluoxetine	0.018 mg/L (18 μg/L)	72 h	A 50% inhibition	n.d.	[153]
Scenedesmus vacuolatus	Fluoxetine	0.024*(pH=10)*-0.207*(pH=6.5)* mg/L [24*(pH=10)*-207*(pH=6.5)* μg/L]	24 h	A 50% toxicity	n.d.	[152]

Chlorella pyrenoidosa<br>*Chlorella ellipsoidea*<br>*Scenedesmus obliquus*<br>*Scenedesmus quadricauda*<br>*Dunaliella salina*<br>*Dunaliella parva*<br>*Chlamydomonas microsphaera*	Fluoxetine	0.64 mg/L<br>0.77 mg/L<br>0.07 mg/L<br>0.09 mg/L<br>0.09 mg/L<br>0.05 mg/L<br>0.25 mg/L	96 h	A 50% inhibition	n.d.	[151]
Pseudokirchneriella subcapitata	Fluoxetine<br>Sertraline<br>Duloxetine<br>Clomipramine<br>Paroxetine<br>Amitriptyline<br>Fluvoxamine<br>Mianserine<br>Citalopram<br>Venlafaxine<br>Milnacipran	0.2 mg/L<br>0.15 mg/L<br>0.37 mg/L<br>0.46 mg/L<br>0.63 mg/L<br>0.72 mg/L<br>0.98 mg/L<br>2.1 mg/L<br>3.3 mg/L<br>47.5 mg/L<br>61.3 mg/L	72 h	A 50% inhibition	n.d.	[115]
Pseudokirchneriella subcapitata	Fluoxetine<br>Sertraline<br>Fluvoxamine<br>Paroxetine<br>Citalopram	0.027 mg/L<br>0.043 mg/L<br>0.062 mg/L<br>0.14 mg/L<br>1.6 mg/L	48 h	A 50% toxicity	n.d.	[149]
Skeletonema marinoi	Fluoxetine<br>Duloxetine<br>Clomipramine<br>Amitriptyline<br>Sertraline<br>Fluvoxamine<br>Paroxetine<br>Citalopram<br>Venlafaxine	0.048 mg/L (48 μg/L)<br>0.0019 mg/L (1.9 μg/L)<br>0.0033 mg/L (3.3 μg/L)<br>0.0438 mg/L (43.8 μg/L)<br>0.0618 mg/L (61.8 μg/L)<br>0.114 mg/L (114.5 μg/L)<br>0.121 mg/L (121 μg/L)<br>0.5 mg/L (500 μg/L)<br>6.9 mg/L (6900 μg/L)	72 h	A 50% inhibition	n.d.	[148]
Pseudokirchneriella subcapitata<br>*Scenedesmus acutus*<br>*Scenedesmus quadricauda*<br>*Chlorella vulgaris*	Fluoxetine	0.045 mg/L (45 μg/L)<br>0.091 mg/L (91 μg/L)<br>0.213 mg/L (213 μg/L)<br>4.339 mg/L (4339 μg/L)	96 h	A 50% inhibition	n.d.	[150]
Pseudokirchneriella subcapitata<br>*Scenedesmus*		4 mg/L (4003 μg/L)<br>3.62 mg/L				

acutus<br>*Scenedesmus quadricauda*<br>*Chlorella vulgaris*	Fluvoxamine	(3620 μg/L)<br>3.563 mg/L<br>(3563 μg/L)<br>10.208 mg/L<br>(10208 μg/L)	96 h	A 50% inhibition	n.d.	[150]
Pseudokirchneriella subcapitata<br>*Scenedesmus acutus*<br>*Scenedesmus quadricauda*<br>*Chlorella vulgaris*	Sertraline	0.012 mg/L<br>(12 μg/L)<br>0.099 mg/L<br>(99 μg/L)<br>0.317 mg/L<br>(317 μg/L)<br>0.763 mg/L<br>(763 μg/L)	96 h	A 50% inhibition	n.d.	[150]
Scenedesmus vacuolatus	Trimipramine	0.738*(pH=10)*-21.730*(pH=6.5)* mg/L<br>[738*(pH=10)*-21730*(pH=6.5)* μg/L]	24 h	A 50% toxicity	n.d.	[152]
Pseudokirchneriella subcapitata	(±)Sulpiride	99.8 mg/L	72 h	A 50% inhibition	n.d.	[116]
Pseudokirchneriella subcapitata	Norfluoxetine	0.242 mg/L<br>(242 μg/L)	24 h	A 50% inhibition	n.d.	[146]
Scenedesmus vacuolatus	Norfluoxetine	0.066*(pH=10)*-0.531*(pH=6.5)* mg/L<br>[66*(pH=10)*-531*(pH=6.5)* μg/L]	24 h	A 50% toxicity	n.d.	[152]
Chlorella vulgaris<br>*Microcystis aeruginosa*	Sertraline-HCl	0.2 mg/L<br>(200 μg/L)<br>0.2 mg/L<br>(200 μg/L)	7 days	A 57% inhibition<br>A 69% inhibition	n.d.	[154]
Pseudokirchneriella subcapitata	Sertraline-HCl	0.14 mg/L	72 h	A 50% inhibition	n.d.	[155]
		Lipid regulators				
Pseudokirchneriella subcapitata	Gemfibrozil	15 mg/L	72 h	A 50% inhibition	n.d.	[156]
Pseudokirchneriella subcapitata	Gemfibrozil	29.7 mg/L	72 h	A 50% inhibition	n.d.	[136]
Pseudokirchneriella subcapitata	Gemfibrozil	49 mg/L	72 h	A 50% inhibition	n.d.	[157]
Anabaena sp.	Gemfibrozil	4-9 mg/L	1-24 h	A 50% toxicity	n.d.	[158]
Pseudokirchneriella subcapitata	Fenofibrate	19.8 mg/L	72 h	A 50% inhibition	n.d.	[156]
Pseudokirchneriella subcapitata	Bezafibrate	60 mg/L	72 h	No inhibition	n.d.	[156]
Pseudokirchneriella subcapitata	Bezafibrate	100 mg/L	72 h	No inhibition	n.d.	[116]
Pseudokirchneriella subcapitata	Bezafibrate	103 mg/L	72 h	A 50% inhibition	n.d.	[157]
Anabaena sp.	Bezafibrate	7-41 mg/L	1-24 h	A 50% toxicity	n.d.	[158]
Dunaliella tertiolecta	Simvastatin	22.8 mg/L	96 h	A 50% toxicity	n.d.	[139]
Tetraselmis chuii	Clofibrate	39.7 mg/L	96 h	A 50% inhibition	n.d.	[159]
Tetraselmis chuii	Clofibric acid	318 mg/L	96 h	A 50% inhibition	n.d.	[159]

Scenedesmus subspicatus	Clofibric acid	89 mg/L	72 h	A 50% inhibition	n.d.	[143]
Desmodesmus subspicatus	Clofibric acid	115 mg/L	3 days	A 50% inhibition	n.d.	[160]
Chlorella pyrenoidosa	Clofibric acid	100 mg/L - 150 mg/L	8 days	A ~50% inhibition	The whole cell	[107]
Pseudokirchneriella subcapitata	Clofibric acid	75 mg/L	96 h	A 50% toxicity	n.d.	[137]
Pseudokirchneriella subcapitata<br>*Synechococcus leopolensis*	Clofibric acid	94 mg/L<br>40.2 mg/L	96 h	A 50% toxicity	n.d.	[82]
Anabaena sp.	Clofibric acid	30-48 mg/L	1-24 h	A 50% toxicity	n.d.	[158]
Dunaliella tertiolecta	Clofibric acid	1 mg/L	96 h	No inhibitory effect	n.d.	[161]
Dunaliella tertiolecta	Clofibric acid	224 mg/L	96 h	A 50% toxicity	n.d.	[139]
		Anti-neoplastic agents				
Pseudokirchneriella subcapitata	5-fluorouracil<br>Cisplatin<br>Etoposide<br>Imatinib mesylate	0.13 mg/L<br>1.52 mg/L<br>30.4 mg/L<br>2.29 mg/L	72 h	A 50% inhibition	n.d.	[162]
Raphidocelis subcapitata	5-fluorouracil<br>Tamoxifen<br>Imatinib<br>Methotrexate<br>Cyclophosphamide<br>Ifosfamide	0.075 mg/L<br>>0.2 mg/L<br>5 mg/L<br>9.5 mg/L<br>>100 mg/L<br>>100 mg/L	72 h	A 50% toxicity	n.d.	[163]
Pseudokirchneriella subcapitata<br>*Chlorella vulgaris*<br>*Chlamydomonas reinhardtii*	Tamoxifen	0.98 mg/L<br>0.61 mg/L<br>0.47 mg/L	72 h	A 50% inhibition	n.d.	[164]
Scenedesmus obliquus<br>*Desmodesmus quadricauda*<br>*Microcystis aeruginosa*<br>*Raphidocelis subcapitata*	5-fluorouracil	30.5 mg/L<br>20.5 mg/L<br>6 mg/L<br>0.96 mg/L	72 h	A 50% toxicity	n.d.	[103]
Desmodesmus subspicatus	5-fluorouracil<br>Cytarabine<br>Gemcitabine	45-53 mg/L	72 h	A 50% toxicity	n.d.	[165]
Scenedesmus subspicatus	Methotrexate	260 mg/L	72 h	A 50% inhibition	n.d.	[143]
Skeletonema pseudocostatum	Thioguanine	0.014 mg/L<br>(14.2 μg/L)<br>[8.5*10-8 mol/L]	72 h	A 50% inhibition	n.d.	[153]
Synechococcus leopoliensis	Cisplatin<br>5-fluorouracil<br>Imatinib mesylate<br>Etoposide	0.67 mg/L<br>1.2 mg/L<br>5.36 mg/L<br>351 mg/L	72 h	A 50% inhibition<br>No inhibition	n.d.	[162]
Synechococcus leopoliensis	Cyclophosphamide<br>Carboxy-cyclophosphamide	320 mg/L<br>17 mg/L	72 h	No toxicity<br>A 50% toxicity	n.d.	[166]
		Anti-epileptic agents				
Chlamydomonas mexicana				A 23% inhibition		

Chlamydomonas pitschmannii<br>*Micractinium reisseri*<br>*Scenedesmus obliquus*	Carbamazepine	100 mg/L	7 days	A 31% inhibition<br>A 43% inhibition<br>A 60% inhibition	n.d.	[167]
Desmodesmus subspicatus	Carbamazepine	74 mg/L	3 days	A 50% inhibition	n.d.	[160]
Scenedesmus obliquus<br>*Chlorella pyrenoidosa*	Carbamazepine	54(*144h*)-201(*24h*) mg/L<br>33(*144h*)-1339(*24h*) mg/L	24h to 144h	A 50% toxicity<br>A 50% toxicity	n.d.	[168]
Chlorella vulgaris	Carbamazepine	37 mg/L	48 h	A 50% inhibition	n.d.	[169]
Dunaliella tertiolecta	Carbamazepine	53(*24h*)-296(*96h*) mg/L	24h to 96h	A 50% inhibition	Increase in MDA content<br>Increase in Car content<br>Decrease/Increase in Chl content	[170]
Neochloris pseudoalveolaris	Carbamazepine	0.1-1 mg/L	-	A ~29% inhibition	Change in sugar content<br>Decrease in Chl a and Car	[171]
Phaeodactylum tricornutum	Carbamazepine	62 mg/L	72 h	A 50% inhibition	n.d.	[90]
Cyclotella meneghiniana<br>*Synechococcus leopolensis*	Carbamazepine	31.6 mg/L<br>33.6 mg/L	96 h	A 50% toxicity	n.d.	[82]
Pseudokirchneriella subcapitata	Phenytoin	28.3 mg/L	72 h	A 50% inhibition	n.d.	[116]
			Beta-blockers			
Pseudokirchneriella subcapitata	Propranolol	1.86 mg/L	72 h	A 50% inhibition	n.d.	[115]
Pseudokirchneriella subcapitata	Propranolol	0.77 mg/L	72 h	A 50% inhibition	n.d.	[172]
Pseudokirchneriella subcapitata<br>*Cyclotella meneghiniana*<br>*Synechococcus leopolensis*	Propranolol	7.4 mg/L<br>0.24 mg/L<br>0.67 mg/L	96 h	A 50% toxicity	n.d.	[82]
Scenedesmus vacuolatus	Propranolol	0.118*(pH=10)* mg/L-23.6*(pH=6.5)* mg/L	24 h	A 50% toxicity	n.d.	[152]
Scenedesmus vacuolatus	Propranolol	24 mg/L	24 h	A 50% toxicity	n.d.	[173]
Desmodesmus subspicatus	Propranolol	0.63 mg/L	-	A 50% inhibition in photosynthetic activity	n.d.	[84]
Desmodesmus subspicatus	Propranolol	5.8 mg/L	3 days	A 50% inhibition	n.d.	[160]
Acutodesmus obliquus<br>*Chlamydomonas reinhardtii*	Propranolol	19 mg/L<br>3 mg/L	5 h	A 50% toxicity	n.d.	[129]
Chlorella vulgaris	Propranolol	0.259 mg/L<br>2.59 mg/L<br>25.9 mg/L	24 h	F=21%<br>F=39%<br>F=54%	Whole cell	[174]

Skeletonema pseudocostatum	Propranolol	0.236 mg/L (236 μg/L)	72 h	A 50% inhibition	n.d.	[153]
Phaeodactylum tricornutum	Propranolol	0.29 mg/L	72 h	A 50% inhibition	n.d.	[90]
Desmodesmus subspicatus	Metoprolol	7.3 mg/L	3 days	A 50% inhibition	n.d.	[160]
Scenedesmus vacuolatus	Metoprolol	75 mg/L	24 h	A 50% toxicity	n.d.	[173]
Pseudokirchneriella subcapitata	Metoprolol	74 mg/L	72 h	A 50% inhibition	n.d.	[115]
Pseudokirchneriella subcapitata	Atenolol	143 mg/L – 190 mg/L	72 h	A 50% toxicity	n.d.	[175]
Phaeodactylum tricornutum	Atenolol	312 mg/L	72 h	A 50% inhibition	n.d.	[90]
Scenedesmus vacuolatus	Nadolol	>100 mg/L	24 h	A 50% toxicity	n.d.	[173]
			Estrogens			
Raphidocelis subcapitata	Estradiol	3.2 mg/L	96 h	A 15% inhibition	n.d.	[182]
Desmodesmus subspicatus	Ethinylestradiol	12.35 mg/L	-	A 50% inhibition in photosynthetic activity	n.d.	[84]
Pseudokirchneriella subcapitata	Ethinylestradiol	0.01 mg/L<br>0.32 mg/L<br>0.46 mg/L<br>0.8 mg/L	24 h<br>48 h<br>72 h<br>96 h	A 50% toxicity	n.d.	[176]
	Estradiol	0.01 mg/L<br>0.05 mg/L<br>0.3 mg/L<br>0.87 mg/L	24 h<br>48 h<br>72 h<br>96 h	A 50% toxicity	n.d.	
Desmodesmus subspicatus	Ethinylestradiol	0.04 mg/L<br>0.11 mg/L<br>0.48 mg/L<br>0.73 mg/L	24 h<br>48 h<br>72 h<br>96 h	A 50% toxicity	n.d.	[176]
	Estradiol	0.08 mg/L<br>0.42 mg/L<br>0.66 mg/L<br>1.07 mg/L	24 h<br>48 h<br>72 h<br>96 h	A 50% toxicity	n.d.	
Chlorella vulgaris	Ethinylestradiol	122 mg/L<br>111 mg/L<br>70 mg/L	7 days<br>10 days<br>14 days	A 50% inhibition	n.d.	[178]
	Estradiol	242 mg/L<br>192 mg/L<br>105 mg/L	7 days<br>10 days<br>14 days			
Scenedesmus armatus	Ethinylestradiol	144 mg/L<br>118 mg/L<br>74 mg/L	7 days<br>10 days<br>14 days	A 50% inhibition	n.d.	[178]
	Estradiol	522 mg/L<br>368 mg/L<br>207 mg/L	7 days<br>10 days<br>14 days			
Microcystis aeruginosa<br>*Scenedesmus obliquus*<br>*Desmodesmus quadricauda*<br>*Raphidocelis subcapitata*	Ethynylestradiol	1.48 mg/L<br>0.82 mg/L<br>0.29 mg/L<br>0.21 mg/L	72 h	A 50% toxicity	n.d.	[103]

Anabaena variabilis	Ethynylestradiol	100 mg/L<br>71 mg/L<br>83 mg/L	7 days<br>10 days<br>14 days	A 50% toxicity	n.d.	[181]
	Estradiol	2002 mg/L<br>1281 mg/L<br>438 mg/L	7 days<br>10 days<br>14 days			
Desmodesmus communis	Ethynylestradiol	0.08-0.1 mg/L (80-100 μg/L)	72 h	A 50% inhibition	n.d.	[177]
Dunaliella salina	Ethynylestradiol	0.001 mg/L (1 μg/L)	11 days	A 50% inhibition	Decrease in Chl a, Chl b and total Car content. Increase in SOD activity, decrease in CAT and GPx activity. Decrease in PUFA % content.	[179]
Navicula incerta	Ethynylestradiol<br>Estradiol	3.2 mg/L<br>>10 mg/L	96 h	A 50% inhibition	Decrease in protein content and polysaccharide content, increase in lipid content (EE2 at 3 mg/L, E2 at 10 mg/L)<br>For EE2 at 3.5 mg/L: Increase in SOD and GST activity, decrease in POD activity.<br>For E2 at 10 mg/L: Increase in SOD, POD and GST activity.	[180]
			Anesthetic drugs			
Scenedesmus vacuolatus	Lidocaine	12-1491 mg/L (0.05-6.36 mM)	24 h	A 50% toxicity	n.d.	[152]
			Anti-anxiety agents			
Tetraselmis chuii	Diazepam	16.5 mg/L	96 h	A 50% inhibition	n.d.	[159]
			Proton pump inhibitors			
Tetraselmis suecica	Omeprazole	114 mg/L	24 h	A 50% inhibition	ROS generation	[183]
			Antiviral drugs			
Desmodesmus subspicatus	Oseltamivir ethylester	6400 mg/L (6.4 g/L)	24 h	A 50% inhibition in photosynthetic activity	n.d.	[184]
Pseudokirchneriella subcapitata	Oseltamivir ethylester	210 mg/L	24 h	A 50% inhibition	n.d.	[184]
Pseudokirchneriella subcapitata	Oseltamivir ethylester	352 mg/L	96 h	A 50% inhibition	n.d.	[185]
			Antiparasitic drugs			
Scenedesmus vacuolatus	Flubendazole<br>Fenbendazole	>1 mg/L	24 h	Toxic/inhibitory effect	n.d	[186]
Scenedesmus subspicatus	Abamectin	4.4 mg/L	72 h	A 50% toxicity	n.d.	[187]
			Antiallergic agents			
Pseudokirchneriella subcapitata	Diphenhydramine<br>Epinastine	1.24 mg/L<br>3.75 mg/L	72 h	A 50% inhibition	n.d.	[116]
			ACE inhibitors			
Desmodesmus subspicatus	Captopril	168 mg/L	3 days	A 50% inhibition	n.d.	[160]
			Antiarrhythmic agents			
Tetraselmis chuii	Procainamide	104 mg/L	96 h	A 50% reduction	n.d.	[70]
			Quinazolines			

Microcystis aeruginosa	Chlorophenylmethoxyphenyl quinazoline	1.93 mg/L	96 h	A 50% inhibition	n.d.	[189]
		Antiseptic/preservative/disinfectant ingredients				
Pseudokirchneriella subcapitata	Triclosan	0.0005 mg/L (0.53 µg/L)	72 h	A 50% toxicity	n.d.	[190]
Pseudokirchneriella subcapitata	Triclosan	0.0005 mg/L 0.53 µg/L	72 h	A 50% inhibition	n.d.	[81]
Pseudokirchneriella subcapitata	Triclosan	0.025 mg/L (25 µg/L)	72 h	A 50% toxicity	n.d.	[191]
Pseudokirchneriella subcapitata	Triclosan	0.037 mg/L (37 µg/L)	72 h	A 50% inhibition	n.d.	[157]
Raphidocelis subcapitata	Triclosan	0.0078 mg/L (7.8 µg/L)	72 h	A 50% inhibition	n.d.	[192]
Selenastrum capricornutum	Triclosan	0.0047 mg/L (4.7 µg/L)	72 h	A 50% inhibition	n.d.	[193]
Chlorella sp.	Triclosan	1 mg/L	72 h	A 50% inhibition	n.d.	[200]
Chlorella pyrenoidosa<br>*Chlorella ellipsoidea*<br>*Scenedesmus obliquus*<br>*Scenedesmus quadricauda*<br>*Dunaliella salina*<br>*Dunaliella parva*<br>*Chlamydomonas microsphaera*	Triclosan	1 mg/L<br>1.44 mg/L<br>0.2 mg/L<br>0.37 mg/L<br>0.16 mg/L<br>0.04 mg/L<br>1.35 mg/L	96 h	A 50% inhibition	n.d.	[151]
Chlamydomonas reinhardtii	Triclosan	0.4 mg/L	96 h	A 36% inhibition	Whole cell/Increase in MDA content/Change in antioxidant activity	[194]
Chlamydomonas reinhardtii	Triclosan	4 mg/L	24 h	A 50% toxicity	ROS generation	[195]
Scenedesmus subspicatus	Triclosan	0.0014 mg/L (1.4 µg/L)	96 h	A 50% toxicity	n.d.	[197]
Scenedesmus vacuolatus	Triclosan	0.0037 mg/L (3.7 µg/L)	24 h	A 50% inhibition in photosynthetic yield	n.d.	[198]
Desmodesmus sp.<br>*Chlorella pyrenoidosa*<br>*Scenedesmus obliquus*	Triclosan	0.4 mg/L	4 days	A 48% inhibition<br>A 65% inhibition<br>No inhibition	n.d.	[199]
Dunaliella tertiolecta	Triclosan	0.0035 mg/L (3.5 µg/L)	96 h	A 50% toxicity	n.d.	[139]
Tetraselmis suecica	Triclosan	0.8 mg/L	24 h	A 83% inhibition	Whole cell	[196]
Skeletonema pseudocostatum	Triclosan	0.027 mg/L	72 h	A 50% inhibition	n.d.	[153]
Navicula sp.	Triclosan	0.145-0.173 mg/L (145-173 µg/L)	24 h – 72 h	A 50% toxicity	Decreased Ch *a* content	[201]
Microcystis aeruginosa	Triclosan	0.0092 mg/L (9.2 µg/L)	96 h	A 50% inhibition	Whole cell	[203]

Anabaena flosaquae	Triclosan	0.0016 mg/L (1.6 μg/L)	96 h	A 50% toxicity	n.d.	[197]
Pseudokirchneriella subcapitata	Triclocarban	0.0057 mg/L (5.7 μg/L)	72 h	A 50% toxicity	n.d.	[190]
Pseudokirchneriella subcapitata	Triclocarban	0.017 mg/L (17 μg/L)	72 h	A 50% inhibition	n.d.	[81]
Skeletonema pseudocostatum	Cetrimonium bromide	1.64 mg/L	72 h	A 50% inhibition	n.d.	[153]
Scenedesmus obliquus	Climbazole	2 mg/L	12 days	A 50% inhibition	n.d.	[204]
Pseudokirchneriella subcapitata	Benzylparaben<br>i-butylparaben<br>n-butylparaben	1.2 mg/L<br>4 mg/L<br>9.5 mg/L	96 h	A 50% inhibition	n.d.	[205]
Pseudokirchneriella subcapitata	Propylparaben<br>Butylparaben<br>Ethylparaben	0.17 mg/L<br>0.1 mg/L<br>0.4 mg/L	48 h	A 50% inhibition	n.d.	[140]
Pseudokirchneriella subcapitata	Methylparaben	35 mg/L	72 h	A 50% inhibition	n.d.	[191]
Chlorella vulgaris	Trichloroisocyanuric acid	0.313 mg/L	96 h	A 50% inhibition	Uplift of CAT activity	[105]
Chlorella vulgaris	Trichloroisocyanuric acid	0.12 mg/L	72 h	A 50% inhibition	n.d.	[106]

n.d.- no data available to the best of the authors' knowledge.
